# FBCA: Flexible Besiege and Conquer Algorithm for Multi-Layer Perceptron Optimization Problems

**DOI:** 10.3390/biomimetics10110787

**Published:** 2025-11-19

**Authors:** Shuxin Guo, Chenxu Guo, Jianhua Jiang

**Affiliations:** 1 Center for Artificial Intelligence, Jilin University of Finance and Economics, Changchun 130117, China; guoshuxin@jlufe.edu.cn (S.G.); 6241191017@s.jlufe.edu.cn (C.G.); 2 Jilin Province Key Laboratory of Fintech, Jilin University of Finance and Economics, Changchun 130117, China

**Keywords:** metaheuristic algorithm, besiege and conquer algorithm (BCA), perturbation mechanism, swarm intelligence, multi-layer perceptron (MLP)

## Abstract

A Multi-Layer Perceptron (MLP), as the basic structure of neural networks, is an important component of various deep learning models such as CNNs, RNNs, and Transformers. Nevertheless, MLP training faces significant challenges, with a large number of saddle points and local minima in its non-convex optimization space, which can easily lead to gradient vanishing and premature convergence. Compared with traditional heuristic algorithms relying on a population-based parallel search, such as GA, GWO, DE, etc., the Besiege and Conquer Algorithm (BCA) employs a one-spot update strategy that provides a certain level of global optimization capability but exhibits clear limitations in search flexibility. Specifically, it lacks fast detection, fast adaptation, and fast convergence. First, the fixed sinusoidal amplitude limits the accuracy of fast detection in complex regions. Second, the combination of a random location and fixed perturbation range limits the fast adaptation of global convergence. Finally, the lack of a hierarchical adjustment under a single parameter (BCB) hinders the dynamic transition from exploration to exploitation, resulting in slow convergence. To address these limitations, this paper proposes a Flexible Besiege and Conquer Algorithm (FBCA), which improves search flexibility and convergence capability through three new mechanisms: (1) the sine-guided soft asymmetric Gaussian perturbation mechanism enhances local micro-exploration, thereby achieving a fast detection response near the global optimum; (2) the exponentially modulated spiral perturbation mechanism adopts an exponential spiral factor for fast adaptation of global convergence; and (3) the nonlinear cognitive coefficient-driven velocity update mechanism improves the convergence performance, realizing a more balanced exploration–exploitation process. In the IEEE CEC 2017 benchmark function test, FBCA ranked first in the comprehensive comparison with 12 state-of-the-art algorithms, with a win rate of 62% over BCA in 100-dimensional problems. It also achieved the best performance in six MLP optimization problems, showing excellent convergence accuracy and robustness, proving its excellent global optimization ability in complex nonlinear MLP optimization training. It demonstrates its application value and potential in optimizing neural networks and deep learning models.

## 1. Introduction

The Multi-Layer Perceptron (MLP) [1], as an early representative of deep neural networks (DNNs) [2], holds a crucial position in the development of neural networks. The success of MLP has inspired the development of numerous subsequent deep learning models, such as Convolutional Neural Networks (CNNs) [3], Transformers [4], YOLO [5], and DeepSeek [6], significantly advancing the application of artificial intelligence across various fields. Despite its strong performance in tasks such as pattern recognition, classification, and regression, MLP faces several challenges during training. These include the non-convexity and high-dimensional nonlinearity of its weight-bias space, which often causes the model to get stuck in saddle points or local minima [7,8], limiting its generalization ability and performance improvement.

Against this backdrop, Metaheuristic Algorithms (MAs), as global optimization strategies, have gradually become effective tools for addressing these issues [9]. In recent years, many metaheuristic algorithms have been incorporated into MLP training, achieving positive results. For instance, the Grey Wolf Optimizer (GWO) in swarm intelligence has excelled in MLP classification tasks [10], while Particle Swarm Optimization (PSO) has outperformed Stochastic Gradient Descent (SGD) in problems like estimating the vertical dispersion coefficient of a natural flow, demonstrating the effectiveness of metaheuristic methods in MLP weight-bias optimization [11]. Genetic algorithms (GAs) have been used in evolutionary algorithms to optimize MLP hyperparameters and achieve 100% key recovery rate in AES side-channel attacks, which is significantly better than stochastic and Bayesian baselines [12]. In addition, algorithms such as the Slime Mould Algorithm (SMA) [13], Black-winged Kite Algorithm (BKA) [14], and Harris Hawks Optimization (HHO) [15] also showed good optimization performance in different MLP training tasks. Some studies also try to construct hybrid models or multi-objective optimization models, such as MLP-PSODE fused with PSO and differential evolution (DE) for suspended sediment load estimation [16], and the MLP-MOSSA model based on the multi-objective Salp Swarm Algorithm for water evaporation prediction [17]. These studies fully show that the combination of MLP and metaheuristic algorithms has become an important research direction in the field of optimization [18,19].

However, although many optimization algorithms have provided solutions for MLP, most of these algorithms rely on parallel search structures. While they offer some search efficiency, they may not fully leverage strategies that combine exploration around the optimal solution with random position exploration in the process of finding the global optimum. When designing the original Besiege and Conquer Algorithm (BCA) [20,21], this consideration was fully incorporated and transformed into an advantage in the optimization process. BCA balances global optimization ability with local exploitation, and although it has achieved some success in MLP optimization problems, it still has certain limitations: search dynamics can become rigid, it can easily get stuck in local minima, and there is an insufficient balance between global exploration and local exploitation. These shortcomings limit the flexibility of its global optimization capability and constrain its performance in complex nonlinear problems such as MLP weight-bias optimization. To address these shortcomings, this paper proposes an improved Flexible Besiege and Conquer Algorithm (FBCA), with the following key research motivations and contributions summarized.

### 1.1. Motivation

Although the original BCA demonstrates certain optimization capability, its inherent limitations restrict global search efficiency and convergence performance. To enhance its adaptability and robustness in complex optimization scenarios, this study proposes the Flexible Besiege and Conquer Algorithm (FBCA), inspired by three main research motivations.

Motivation 1: BCA possesses structure advantages and special mechanisms over traditional MAs like GA [22] and DE [23]. First, regarding particle generation, BCA introduces a more detailed hierarchical structure: population–army(sub-population)–soldier(particle), whereas traditional algorithms typically employ a simple population–particle. Second, BCA controls the exploration and exploitation through binary gates, using sine and cosine factors to guide single-point besieges and random perturbations, unlike GA and DE, which use a uniform F/CR or crossover rate parameter. This design makes BCA easier to understand, implement, and improve and allows for flexible application to optimization scenarios such as MLP.

Motivation 2: The exploitation phase of BCA primarily relies on a sine-based factor to guide the search direction. Although this mechanism can converge to a certain extent to a better solution, due to the limited search direction, the population is prone to fall into the local optimum and lacks the ability to further explore the solution space in detail. Therefore, it is necessary to consider introducing mechanisms with perturbative and flexible exploration at the exploitation stage to enhance the accuracy of local search and the ability of solution refinement.

Motivation 3: The global search process in BCA depends on sine and cosine factors within a fixed interval [−1, 1]. Although this periodic driving mechanism promotes early-stage diversity, premature convergence may occur if exploitation begins before reaching promising regions. To strengthen global exploration, an adaptive and dynamically regulated position-update mechanism is needed to achieve a more flexible and effective global search.

Motivation 4: In BCA, the transition between exploration and exploitation is governed by a single binary control gate BCB. This fixed control structure restricts the algorithm’s flexibility during phase transitions. Once a branch is selected, soldiers can only follow fixed update rules, preventing dynamic balance at the mechanism level. Hence, a hierarchical and self-adaptive control structure is proposed, refining the new mechanisms within both BCB branches and introducing additional binary gates to achieve a more flexible exploration–exploitation balance in FBCA.

### 1.2. Contributions

The main contributions of this paper are summarized as follows:

- Sine-Guided Soft Asymmetric Gaussian Perturbation Mechanism: an optimization mechanism that integrates Gaussian flexible micro-perturbations under sine factor guidance, enhancing the ability to quickly detect high-precision solutions, reducing the risk of local stagnation.
- Exponentially Modulated Spiral Perturbation Mechanism: a position update mechanism that applies exponential modulation through an adaptive spiral factor to improve population diversity and ensure fast-adaptive global convergence.
- Nonlinear Cognitive Coefficient-Driven Velocity Update Mechanism: drawing on the PSO’s velocity-based update mechanism, the nonlinear cognitive coefficient dynamically regulates the soldier position update, thereby improving fast-convergent performance and achieving a balanced exploration–exploitation trade-off.
- Validation on IEEE CEC 2017 and Six MLP Problems: extensive experiments on the IEEE CEC 2017 benchmark set (30D, 50D, and 100D) demonstrate FBCA’s excellence in numerical accuracy, convergence behavior, stability, and the Wilcoxon rank sum test. Notably, FBCA shows outstanding performance in high-dimensional composite function optimization. Moreover, in six MLP optimization problems, FBCA achieves an order-of-magnitude lead in the mean results of MSE on XOR and Heart datasets, surpassing the original BCA and other state-of-the-art algorithms such as SMA in function approximation problems.

## 2. Related Work

### 2.1. BCA: Besiege and Conquer Algorithm

This section reviews the BCA [21], which explicitly divides the optimization process into exploration and exploitation, controlled by the parameter BCB. The exploitation phase focuses on generating new soldiers around the best army using a sine-based factor, while the exploration phase updates soldier positions around random armies through a cosine-based factor. The BCA population is randomly initialized within the defined upper and lower bounds. It then alternates between exploration and exploitation based on the BCB parameter to estimate the optimal solution for continuous optimization problems. The detailed pseudocode is presented in Algorithm 1.

**Algorithm 1** The pseudocode of the BCA
  1:**Input:** Population Size: N, Problem Dimension: D, Max Iteration: Max_Gen, The Number Of Soldiers: nSoldiers  2:**Output:** Obtained best solution  3:Initialize the BCB parameters  4:Initialize the solutions’ positions randomly  5:**while** t ← 1 to Max_Gen **do**  6:     **for** i ← 1 to nArmies **do**  7:           **for** j ← 1 to nSoldiers **do**  8:                 **for** d←1 to D **do**  9:                       **if** rand < BCB **then**10:                             Update the position by Equation (1)11:                             Update the position that exceeds the search boundaries By Equation (3)12:                       **else**13:                             Update the position by Equation (2)14:                             Update the position that exceeds the search boundaries By Equation (4)15:                       **end if**16:                 **end for**17:           **end for**18:     **end for**19:     Update gBest and gBestPos.20:
**end while**



When rand < BCB, it is defined as BCA exploitation, and when rand ≥ BCB it is defined as BCA exploration. The BCA divides the entire population into multiple armies, each consisting of a fixed number of soldiers, which can be regarded as the descendants of their respective armies. When a soldier achieves a better fitness value than its current army, it becomes the leader of that army. During the exploitation phase, the update rule for soldiers within each army is defined by Equation (1), while in the exploration phase, it follows Equation (2). The parameters α and β are random values within the range [0, 2π].

(1) $$S_{j,d}^{t+1}=B_{d}^{t}+\left|A_{r,d}^{t}-A_{i,d}^{t}\right|\times sin\left(\alpha \right)\;\;rand<BCB$$

(2) $$S_{j,d}^{t+1}=A_{r,d}^{t}+\left|A_{r,d}^{t}-A_{i,d}^{t}\right|\times cos\left(\beta \right)\;\;rand\geq BCB$$

Meanwhile, BCA also accounts for soldier position updates that exceed the search boundaries during exploration and exploitation. If the soldier crosses the border in algorithm exploitation will use Equation (3) for correction processing, if the soldier crosses the border in algorithm exploration, it will use Equation (4) for correction processing., where $S_{j,d}^{t+1}$ is the $j_{th}$ soldier of the $d_{th}$ dimension of the t+1 iteration, $B_{d}^{t}$ is the best army of the current $t_{th}$ iteration, $A_{i,d}^{t}$ is the $i_{th}$ army of the $d_{th}$ dimension of the *t* iteration, $A_{r,d}^{t}$ is the random army of the $d_{th}$ dimension of the *t* iteration, and BCB is set to a fixed value of 0.8.

(3) $$S_{j,d}^{t+1}=BCB_{d}^{t}\times B_{d}^{t}+\left(1-BCB_{d}^{t}\right)\times A_{i,d}^{t}$$

(4) $$S_{j,d}^{t+1}=lb+\left(ub-lb\right)\times rand$$

### 2.2. PSO: Particle Swarm Optimization

The particle swarm optimization (PSO) algorithm [24], proposed by Kennedy and Eberhart in 1995, is a swarm intelligence optimization algorithm that simulates the collaborative behavior of bird or fish populations in a search space. The optimization process is as follows: firstly, the initial particle swarm is randomly generated in the search range according to the upper and lower bounds of the variables, and each particle is assigned a position and velocity; subsequently, the particle state is updated through iteration, and the fitness function is used to determine the quality of the solution and guide the search direction.

(5) $$x_{i}\left(t+1\right)=x_{i}\left(t\right)+v_{i}\left(t+1\right)$$

(6) $$v_{i}\left(t+1\right)=w\times v_{i}\left(t\right)+c1\times r1\times \left(p_{i}\left(t\right)-x_{i}\left(t\right)\right)+c2\times r2\times \left(g\left(t\right)-x_{i}\left(t\right)\right)$$

For the $i_{th}$ particle, its position and velocity update equations are defined in Equations (5) and (6): where $x_{i}\left(t\right)$ and $v_{i}\left(t\right)$ denote the current position and velocity of the particle at the tth iteration, respectively; *w* is the inertia weight to regulate the motion continuity; $c_{1}$ and $c_{2}$ represent the cognitive and social coefficients, respectively, to balance the individual learning and group learning; $r_{1}$, $r_{2}\in \left[0,1\right]$ are random numbers; $p_{i}\left(t\right)$ denotes the particle’s own historical optimal position, and g(t) is the global optimal position of the group.

(7) $$\left\{\begin{array}{cc} w\times v_{i}\left(t\right), & \mathrm{inertia}\;\mathrm{part}\\ c1\times r1\times (p_{i}\left(t\right)-x_{i}\left(t\right)), & \mathrm{cognitive}\;\mathrm{part}\\ c2\times r2\times (g\left(t\right)-x_{i}\left(t\right)), & \mathrm{social}\;\mathrm{part} \end{array}\right.$$

As shown in Equation (7), the particle’s velocity update process can be decomposed into three parts. The inertial part maintains the particle motion trend to expand the search range; the cognitive part guides the particle back to its individual optimal region; and the social part drives the particle to approach the global optimal of the group. The interaction of the three components enables the PSO to achieve a dynamic balance between global exploration and local exploitation, which results in a better global optimization capability and convergence nature.

### 2.3. MLP: Multi-Layer Perceptron

**Figure 1:**
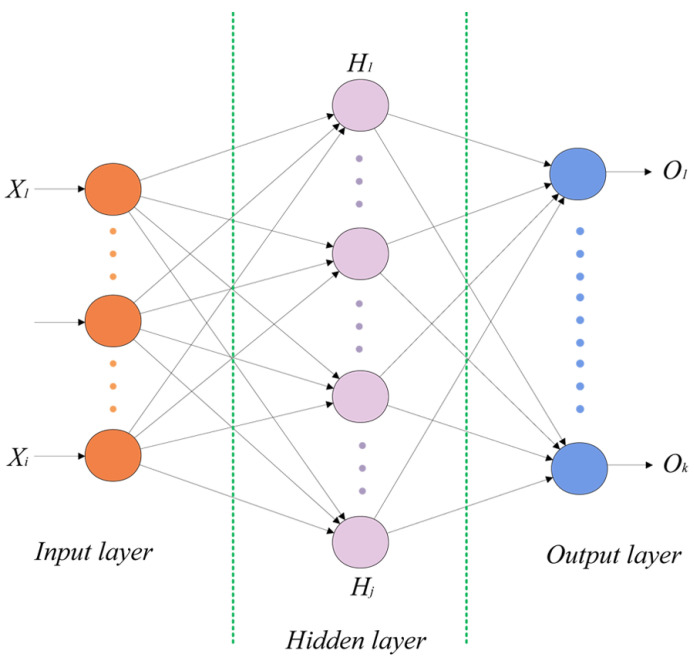
A simple MLP neural network model.

The MLP is a typical feed-forward artificial neural network model [25] consisting of at least three layers of nodes: an input layer, a hidden layer, and an output layer. The example model contains a hidden layer as shown in Figure 1. The orange nodes denote the input layer neurons, the number of which depends on the dimensions of the input features, denoted as $X_{1}$ to $X_{i}$, and the purple nodes denote the hidden layer neurons, denoted as $H_{1}$ to $H_{j}$. The number of layers and the number of nodes in the hidden layer are not fixed, and they can be adjusted according to the specific task requirements and experimental settings. The blue nodes denote the output layer neurons, denoted as $O_{1}$ to $O_{k}$, and their number is usually determined by the training exploitation type.

In the training process of MLP, Mean Squared Error (MSE) is often used as a performance evaluation metric, which is calculated as shown in Equation (8), where *m* denotes the number of output units, $d_{i}^{k}$ is the target output value of the $k_{th}$ training sample in the $i_{th}$ output unit, $o_{i}^{k}$ is the actual output value of the $k_{th}$ training sample at the time of input of that output unit, and *s* denotes the number of training samples in the dataset.

(8) $$\bar{MSE}=\sum_{k=1}^{s}\frac{\sum_{i=1}^{m}{\left(o_{i}^{k}-d_{i}^{k}\right)}^{2}}{s}$$

## 3. Methods

### 3.1. Sine-Guided Soft Asymmetric Gaussian Perturbation Mechanism

The original BCA algorithm was only developed by a sine factor, and its single mechanism can easily lead to the population falling into the local optimum, thus limiting the accuracy of the solution. Therefore, this section proposes the Sine-Guided Soft Asymmetric Gaussian Perturbation Mechanism. Based on the original sine factor, a small Gaussian perturbation [26] is adopted as a correction item to break the original symmetry structure without changing the main search direction, so as to avoid the algorithm falling into the trap of accurate numerical values and improve the probability of the population jumping out of the local optimum.

(9) $$S_{j,d}^{t+1}=B_{d}^{t}+\left|A_{r,d}^{t}-A_{i,d}^{t}\right|\times sin\left(\alpha \right)+N\left(0,0.1^{2}\right)$$

**Figure 2:**
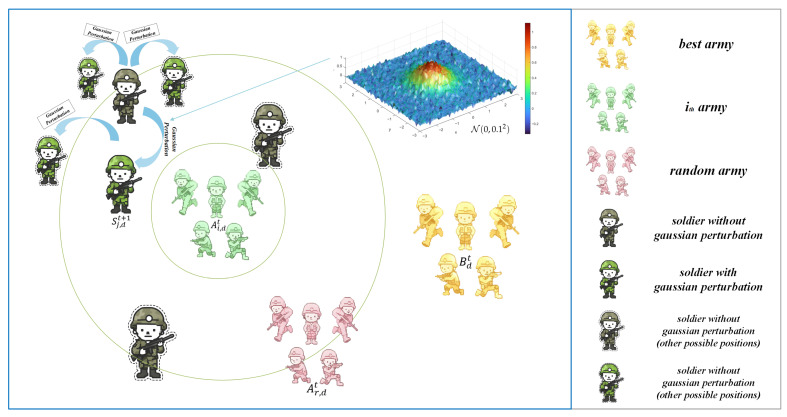
Schematic of the soldier position update mechanism with soft Gaussian perturbation.

As shown in Equation (9), compared with the original BCA exploitation mechanism, this mechanism adopts an additional Gaussian perturbation term of $N(0,0.1^{2})$. The position change in the search space is shown in Figure 2, and the clear comparison between the soldier with Gaussian perturbation and the soldier without Gaussian perturbation reflects the position of the original BCA exploitation mechanism and the soldiers generated by the sine-guided soft asymmetric Gaussian perturbation mechanism, respectively. The original BCA only relies on the position generated by the sine guidance, which may cause the algorithm to miss more refined solutions. By introducing a small Gaussian perturbation, the mechanism introduces slight oscillations while maintaining stability in the original direction, allowing the population to explore potential optimal solutions in adjacent areas, thereby effectively preventing the population from falling into local optimum and further improving the overall convergence accuracy.

### 3.2. Exponentially Modulated Spiral Perturbation Mechanism

The original BCA algorithm has a very fixed exploitation limit, controlled by a single BCB binary gate. However, if the population enters the exploitation phase without fully completing preliminary exploration, convergence often stalls, limiting further improvement in algorithm performance. Therefore, this section details the Exponentially Modulated Spiral Perturbation Mechanism. This mechanism adopts an adaptive spiral perturbation factor [27] to moderately enhance the population’s exploration capabilities, thereby increasing its distribution diversity in the search space, ensuring that the population can escape local optima and prevent premature convergence.

(10) $$Spiral\_Factor=e^{bl}\times cos\left(2\pi l\right)$$

As shown in Equation (10), the main improvement of this paper compared to the original BCA mechanism is that the original cosine coefficient is replaced by a spiral perturbation factor Spiral_factor, the mathematical definition of which is shown in Equation (11). This factor is not a linear spiral, but is an improvement based on the logarithmic spiral. Unlike traditional algorithms that modulate only the exponential parameter *l* [28], the spiral factor proposed in this paper dynamically updates both parameters *b* and *l* simultaneously during the exponential modulation stage to enhance the flexibility and global exploration capability of the search process. The specific update method of parameters *b* and *l* is shown in Equation (12).

(11) $$S_{j,d}^{t+1}=A_{i,d}^{t}+Spiral\_Factor\times \left|A_{r,d}^{t}-A_{i,d}^{t}\right|$$

**Figure 3:**
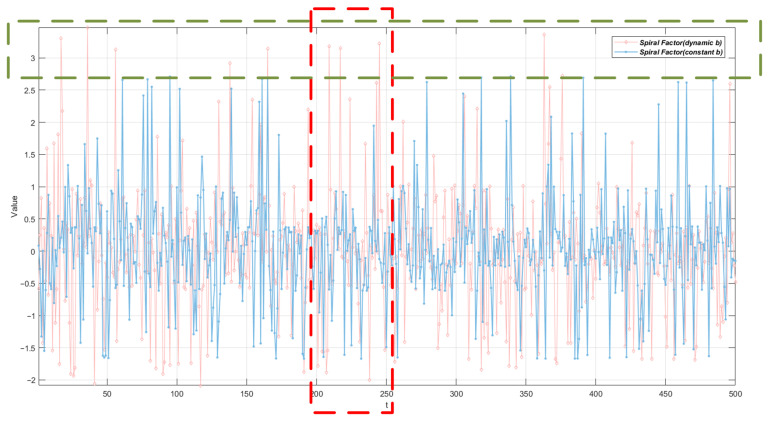
Comparison of spiral factors with constant *b* and dynamic *b*.

As shown in Figure 3, we compared the changing trends of two spiral factors: one is a fixed spiral factor with *b* = 1 [28], and the other is a spiral factor with *b* dynamically updated according to Equation (12). It can be observed in the green dashed box in the figure that the spiral factor with dynamic *b* exhibits a higher peak amplitude, which means that it provides a wider exploration space for the population during the search process. Furthermore, from the variation curve of the 200 to 250 iterations in the red dashed box in the figure, it can be seen that the spiral factor with dynamic parameter *b* can rapidly jump from a value close to 0 to a peak value of approximately 3 within a very short iteration interval. This characteristic fully demonstrates that the proposed exponentially modulated spiral factor has a stronger ability to change instantaneously and escape local extrema, thereby significantly improving the global search performance of the algorithm.

**Figure 4:**
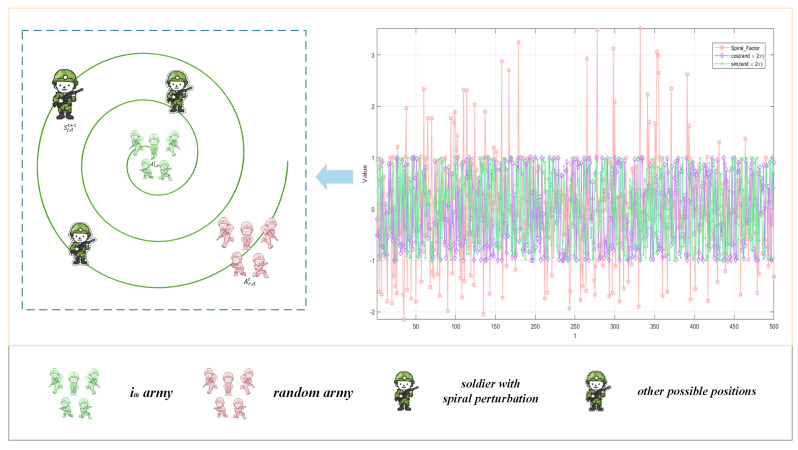
Variation in Spiral_Factor and the soldier position updating with spiral perturbation.

Figure 4 is a schematic diagram of the mechanism, which shows the value changes of Spiral_Factor in 500 iterations and the position distribution of soldiers under spiral perturbation. It can be seen that the value of Spiral_Factor is mainly concentrated in the range of [−2, 3], while the value range of sin(α) and cos(β) used in the original BCA is [−1, 1]. By comparison, it can be found that the value range of Spiral_Factor is approximately twice or more than that of the original BCA sine and cosine factors.

(12) $$\left\{\begin{array}{cc} l=1-(\frac{t}{Max\_Gen}+2)\times rand & \;\\ b=1+0.5\times rand\times \left(1-{\left(t/Max\_Gen\right)}^{2}\right) & \; \end{array}\right.$$

In fact, the core of the improved Spiral Perturbation Mechanism lies in its introduction of an exponentially modulated adaptive spiral perturbation factor. This factor exhibits exponential variation, allowing the Spiral_Factor to rapidly increase from a small value near 0 to 2 or even 3, thereby prompting the population to conduct large-step global exploration and preventing premature convergence. The iterative update method for the parameters b and l in Spiral_Factor is shown in Equation (12), where b is in the range [1, 1.5] and l is a linearly decreasing variable with a range of [−2, 1].

### 3.3. Nonlinear Cognitive Coefficient-Driven Velocity Update Mechanism

The exploration mechanism of the original BCA is driven by the cosine factor, but the long-term use of fixed cosine exploration may lead to the population missing the optimal solution, which makes it difficult to achieve a good balance between exploration and exploitation of the original BCA. To this end, this section adopts the Nonlinear Cognitive Coefficient-Driven Velocity Update Mechanism. By introducing and improving the concept of speed in PSO, this mechanism constructs a method based on random army position [29,30] reference and combined with a dynamically changing cognitive coefficient to drive the soldier position update so that the algorithm can better balance the exploration and exploitation process.

(13) $$S_{j,d}^{t+1}=A_{i,d}^{t}+v_{i,d}^{t}$$

As shown in Equation (13), compared to the original BCA, this mechanism removes the position update term driven by the cosine factor and instead drives the position update with the velocity term $v_{i,d}^{t}$. The specific calculation method of velocity is shown in Equation (14). The velocity formula in Equation (14) refers to the velocity update concept of PSO but is not identical. It mainly improves the cognitive part of PSO while retaining the inertial and social components.

(14) $$v_{i,d}^{t+1}=w\times v_{i,d}^{t}+c1^{\prime }\times r1\times \left(A_{r,d}^{t}-A_{i,d}^{t}\right)+c2\times r2\times \left(B_{d}^{t}-A_{i,d}^{t}\right)$$

**Figure 5:**
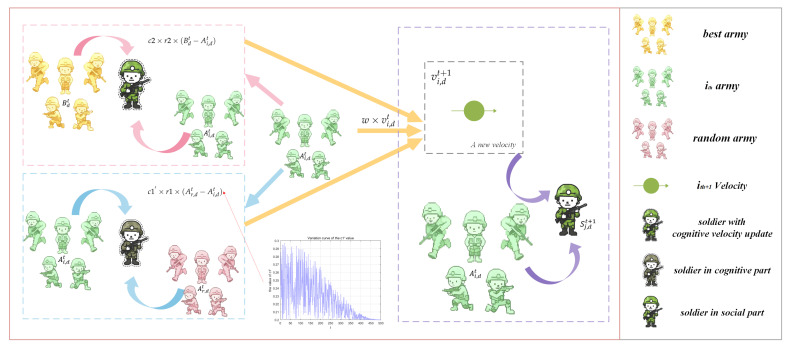
Schematic of nonlinear cognitive coefficient-driven velocity update mechanism.

The reason for retaining the inertial and social components is that they can provide a directional basis for the search and maintain global convergence while considering the global optimal solution. The improvement of the cognitive part of PSO is mainly reflected in the design of the cognitive coefficient $c_{1}$ and its reference position. Specifically, the mechanism replaces the individual optimal position $p_{i}\left(t\right)$ in the traditional PSO with a random army position, thereby enhancing the exploration ability of the population. The setting of the cognitive coefficient $c1^{\prime }$ is shown in Equation (15), and its value shows a nonlinear decreasing trend, ranging from 0.2 to 0.3. The change pattern of $c1^{\prime }$ can be clearly seen from Figure 5.

(15) $$c1^{\prime }=0.2+0.1\times rand\times {\left(1-(t/Max\_Gen)\right)}^{\frac{2t}{Max\_Gen}}$$

The entire Nonlinear Cognitive Coefficient-Driven Velocity Update Mechanism is triggered by a binary gate probability, whose probability expression is shown in Equation (16) and has a value range of [0.1, 0.15]. The purpose of the design of this binary gate is to adopt a small probability PSO velocity update method, so that when the algorithm enters the exploration branch, it can not only maintain the overall exploration capability but also generate “fine search seeds” at a specific moment. This ensures that when the population shows an early convergence trend, some individuals can still quickly approach the best optimal solution through the velocity accumulation effect of PSO. The introduction of this mechanism significantly enhances the exploration–exploitation balance of the algorithm, making the convergence process more stable and efficient.

(16) $$p_{2}=0.1+0.05\times rand\times \sqrt[{10}]{{\left(1-\frac{t}{Max\_Gen}\right)}^{\frac{t}{Max\_Gen}}}$$

### 3.4. FBCA: Flexible Besiege and Conquer Algorithm

To achieve superior algorithmic performance, the Flexible Besiege and Conquer Algorithm (FBCA) was proposed by combining the three aforementioned innovative mechanisms. This section, using flowcharts and algorithm pseudocode, will detail how these three new mechanisms are integrated to form FBCA and how FBCA’s flexibility is demonstrated. Furthermore, the comprehensive improvement effect of FBCA in escaping local optimum, improving convergence capacity, and balancing exploration and exploitation will be illustrated.

(17) $$\mathrm{When}\;r_{1}<BCB:\;S_{j,d}^{t+1}=\left\{\begin{array}{cc} B_{d}^{t}+\left|A_{r,d}^{t}-A_{i,d}^{t}\right|\times sin\left(\alpha \right)+N\left(0,0.1^{2}\right), & \;\mathrm{if}\;\;r_{2}\;<\;p_{1}\\ A_{i,d}^{t}+Spiral\_Factor\times \left|A_{r,d}^{t}-A_{i,d}^{t}\right|, & \;\mathrm{else}\; \end{array}\right.$$

(18) $$\mathrm{When}\;r_{1}\geq BCB:\;S_{j,d}^{t+1}=\left\{\begin{array}{cc} A_{i,d}^{t}+v_{i,d}^{t+1}, & \;\mathrm{if}\;\;r_{3}\;<\;p_{2}\\ A_{r,d}^{t}+\left|A_{r,d}^{t}-A_{i,d}^{t}\right|\times cos\left(\beta \right), & \;\mathrm{else}\; \end{array}\right.$$

First, the overall mathematical expression of FBCA can be seen from Equations (17) and (18), where $r_{1}$, $r_{2}$ and $r_{3}$ are different random values from 0 to 1. Its main improvement lies in further refining the two branches controlled by BCB in the original BCA, introducing probability $p_{1}$ in the original exploitation branch and probability $p_{2}$ in the original exploration branch. When $r_{1}<BCB$, a branch of $r_{2}<p_{1}$ will be triggered; when $r_{1}\geq BCB$, a branch of $r_{3}<p_{2}$ will be triggered. For the handling of the soldier positions that exceed the search boundaries, FBCA still follows the original BCA method: if an out-of-bounds error occurs when $r_{1}<\;BCB$, it is updated according to Equation (3); if an out-of-bounds error occurs when $r_{1}\geq$ BCB, it is updated according to Equation (4).

**Figure 6:**
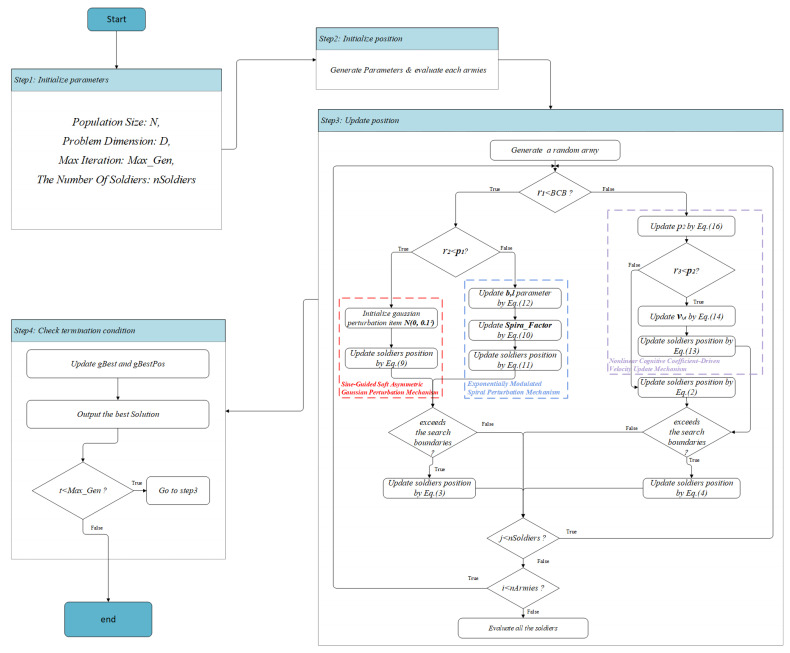
Flow chart of the FBCA.

The control relationship between the two probabilities on the specific mechanism is shown in Figure 6. This flowchart clearly shows the operation logic of FBCA. Among them, $p_{1}$ is the alternating control probability of the Gaussian perturbation mechanism and spiral perturbation mechanism, and its value is fixed at 0.5. When $r_{2}<p_{1}$, the algorithm enters the Gaussian perturbation mechanism stage. First, the Gaussian perturbation term $N(0,0.1^{2})$ is initialized. Then, under the guidance of the sine factor, the perturbation term N is introduced as a correction term into the update of the soldier’s position. When $r_{2}\geq p_{1}$, the spiral perturbation mechanism branch is entered. First, the exponential parameters *b* and *l* of the spiral factor are updated according to Equation (12). Then, the spiral factor is calculated using Equation (10). Finally, it is introduced into Equation (11) to complete the update of the soldier’s position.

With the participation of probability $p_{2}$, the original exploration branch can be divided into two stages: one is to retain the cosine-driven exploration mechanism of the original BCA, and the other is to introduce a cognitive coefficient-driven velocity update mechanism. Before judging whether $r_{3}$ is less than $p_{2}$, the value of $p_{2}$ will be updated according to Equation (16); if $r_{3}<p_{2}$, the cognitive coefficient-driven velocity update mechanism is triggered, and the velocity term with nonlinear coefficient $c_{1}^{\prime }$ is updated through Equation (14), and the velocity term is introduced into Equation (13) to update the soldier position; if $r_{3}\geq p_{2}$, the cosine-driven exploration mechanism is used to update the soldier position.

The pseudocode of FBCA is shown in Algorithm 2. As can be seen from the algorithm flow, FBCA will trigger and experience three new mechanisms with probability during each iteration. From the perspective of creation 1, the adopted Gaussian perturbation term N is a very small asymmetric perturbation, which makes FBCA have slight flexibility and local exploration ability on the basis of maintaining sine-guided exploitation. From the perspective of creation 2, the spiral perturbation factor under exponential modulation is an adaptive change mechanism, and its exponential characteristics drive the population to carry out local fine exploitation, but also realize large-step global exploration, so as to effectively avoid falling into local optimum. From the perspective of creation 3, the nonlinear cognitive coefficient-driven speed update mechanism dynamically generates the position of the soldier so that the algorithm can quickly converge when excellent individuals appear, thereby improving the global convergence performance. In summary, compared with the original BCA, FBCA has adaptive regulation characteristics, which can flexibly switch between exploration and exploitation mechanisms in different iteration stages to achieve a more balanced and efficient global optimized search.

**Algorithm 2** The pseudocode of the FBCA
  1:**Input:** Population Size: N, Problem Dimension: D, Max Iteration: Max_Gen, The Number Of Soldiers: nSoldiers  2:**Output:** Obtained best solution  3:Initialize the solutions’ positions randomly  4:Initialize the velocities and BCB parameters  5:**while** t ← 1 to Max_Gen **do**  6:     **for** i ← 1 to nArmies **do**  7:           **for** j ← 1 to nSoldiers **do**  8:                 **for** d←1 to D **do**  9:                       **if** rand < BCB **then**10:                            **if** rand < $p_{1}$ **then**11:                                 **Creation1: Sine-Guided Soft Asymmetric Gaussian Perturbation**12:                                 initial Gaussian perturbation item N13:                                 Update the soldier position by Equation (9)14:                                 **End Creation1**15:                            **else**16:                                 **Creation2: Exponentially Modulated Spiral Perturbation**17:                                 Update Spiral_Factor by Equation (10)18:                                 Update the soldier position by Equation (11)19:                                 **End Creation2**20:                            **end if**21:                            Update the soldier position that exceeds the search boundaries By Equation (3)22:                       **else**23:                            Update $p_{2}$ by Equation (16)24:                            **if** rand < $p_{2}$ **then**25:                                 **Creation3: Nonlinear Cognitive Velocity Update**26:                                 Update $v_{i,d}$ by Equation (14)27:                                 Update the position by Equation (13)28:                                 **End Creation3**29:                            **else**30:                                 Update the position by Equation (2)31:                            **end if**32:                            Update the soldier position that exceeds the search boundaries By Equation (4)33:                       **end if**34:                 **end for**35:           **end for**36:     **end for**37:     Update gBest and gBestPos.38:
**end while**



### 3.5. Analyzing the Computational Complexity of FBCA

To evaluate the computational complexity of FBCA, we define the following parameters: number of iterations *T*, problem dimension *D*, population size *N*, number of soldiers in each army nSoldiers, and function evaluation cost *c*. The overall computational complexity of FBCA mainly consists of the following parts: problem definition, initialization, soldier position update, and population evaluation.

First, the computational complexity of the problem definition phase is O(1). In the initialization phase, the algorithm needs to initialize soldier positions for $\frac{N}{nSoldier}$ armies in each dimension, thus the complexity is O($\frac{N}{nSoldier}\times D$). In subsequent iterations, the main computational overhead is concentrated in the soldier update and population evaluation phases. The impact of the introduction of the three new mechanisms on the complexity of a single iteration is as follows:

- Sine-Guided Soft Asymmetric Gaussian Perturbation Mechanism: Each dimension needs to perform a sine operation and Gaussian perturbation correction term $N(0,0.1^{2})$ once. The complexity of a single operation is O(1), so the complexity of a single soldier in a single dimension is O(1).
- Exponentially Modulated Spiral Perturbation Mechanism: including the calculation of exponential function (exp), cosine function (cos) and linear parameters *b* and *l*, the single-dimensional operation complexity is also O(1).
- Nonlinear Cognitive Coefficient-Driven Velocity Update Mechanism: It involves the calculation of the nonlinear cognitive coefficient ($c_{1}^{\prime }$) and the solution of speed update formula. The single-dimensional computational complexity is also O(1).

Because each generation of soldiers selects and executes only one of the three mechanisms, each mechanism maintains O(1) one-dimensional complexity. Therefore, the generation complexity of a single soldier is O(*D*). Considering all soldiers and the number of iterations, the overall complexity of the soldier update stage can be expressed as O(nSoldiers×T×D). In the evaluation stage, the algorithm needs to evaluate the fitness of all armies and select the best army in each iteration. At initialization, a total of $\frac{N}{nSoldier}$ armies are generated, and the evaluation cost of each army is *c*. Therefore, the overall evaluation complexity is O($T\times \frac{N}{nSoldier}$×c).

(19) $$\begin{array}{cc} O(FBCA) & =O(problem\;\;definition)+O(initialization)+O(soldier\;\;update)+O(population\;\;evaluation)\\ \; & =O\left(1\right)+O\left(\frac{N}{nSoldier}\right)+O\left(nSoldiers\times T\times D\right)+O\left(T\times \frac{N}{nSoldier}\times c\right)\\ \; & =O(1+\frac{N}{nSoldier}+nSoldiers\times T\times D+T\times \frac{N}{nSoldier}\times c) \end{array}$$

Therefore, the overall complexity metric of FBCA is consistent with the baseline algorithm BCA. The three new mechanisms only introduce constant-level additional computations during the soldier update stage, without increasing the overall complexity cost. This indicates that FBCA successfully improves algorithm performance while maintaining computational efficiency. In summary, the overall computational complexity of FBCA is shown in Equation (19).

## 4. Experiments and Analysis

In this section, we evaluate the performance of FBCA using the IEEE CEC 2017 benchmark function. First, Section 4.1 describes the experimental environment, dataset, and experimental parameter settings. Subsequently, Section 4.2, Section 4.3, Section 4.4, Section 4.5 systematically compare FBCA with 12 other state-of-the-art algorithms and provide a comprehensive performance evaluation of the test results. Section 4.2 conducts qualitative analysis, Section 4.3 conducts quantitative evaluation, Section 4.4 completes statistical testing, and Section 4.5 analyzes the stability of the algorithm. The participating comparison algorithms include the original BCA, the classical algorithms PSO, GA, SCA, the emerging algorithms HOA, COA, PO, HHO, and the high-performance algorithms SMA, CPO, BKA, etc. Section 4.6 performs parameter sensitivity analysis, ablation experiments, and comparison with state-of-the-art (SOTA) algorithms.

### 4.1. Experiment Settings

Environment: The hardware environment used in this study is a computer configured with Intel (R) Core (TM) i7-11800H CPU 2.30 GHz. The code writing was done in the Matlab R2024a environment under the Windows 10 operating system.

Datasets: We used the IEEE CEC 2017 benchmark function set to verify the effectiveness of our algorithm. This function set covers unimodal functions (F1–F2), multimodal functions (F3–F9), hybrid functions (F10–F19), and composite functions (F20–F29). Its global optimal values are known, making it a standard tool for evaluating algorithm performance.

Experiment parameters: To ensure fair comparison between algorithms, we uniformly set the population size to 30 and the maximum number of iterations to 500 in the benchmark function experiments with different dimensions (30D, 50D, and 100D). This parameter setting is intended to ensure the reliability and validity of the experimental results. The detailed parameter configurations of the compared algorithms are shown in Table 1.

### 4.2. Qualitative Analysis

**Figure 7:**
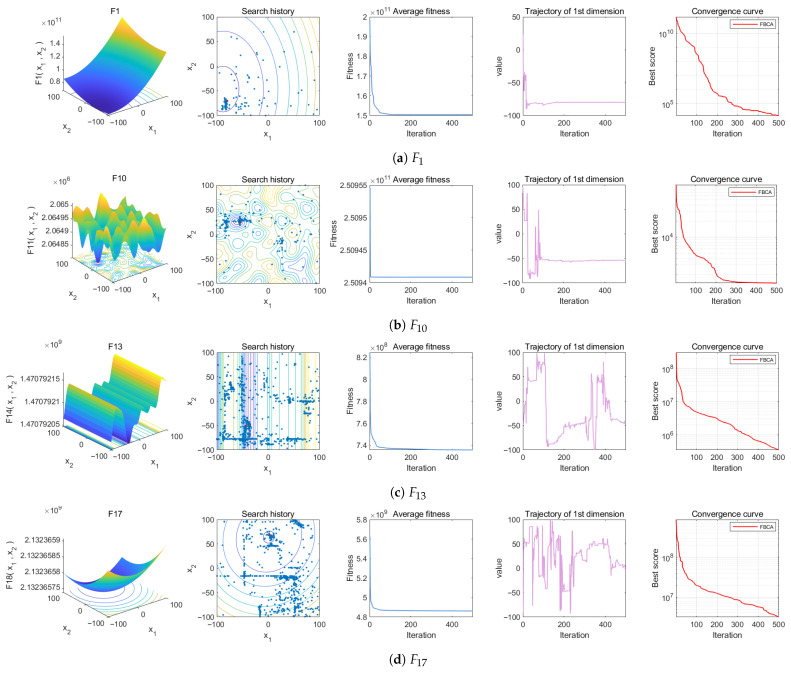
Qualitative analysis experiment of FBCA.

In order to validate and evaluate the performance of FBCA, this section selects the unimodal function F1 to examine the exploitation ability of the algorithm, and the multimodal functions F10, F13, and F17 to evaluate the performance of the algorithm in terms of the exploration–exploitation balance [41]. The search space shape is shown in the first column of Figure 7.

In the search history diagram, the blue points represent the distribution of particles in the population, while contour lines are the projections of the three-dimensional search space onto a two-dimensional plane, reflecting different values of the objective function. The color of the contour lines changes from yellow to green, then to blue and purple, corresponding to the improvement process of fitness; the darker the color, the higher the quality of the solution. Blue points represent the locations searched by particles, while red points represent the current location of the global optimum. Observing the population distribution reveals that, in most cases, blue points are concentrated near red points, indicating that the population is gradually approaching the optimum, fully demonstrating the fine-grained search capability of FBCA during the exploitation phase, as shown in Figure 7a. At the same time, apart from the dense area near the red points, a certain degree of clustering can still be observed in other locations, indicating that FBCA can still maintain good population diversity, as shown in Figure 7c.

In the average fitness graph and the convergence curve graph, both the average fitness graph and the convergence curve graph reflect the fitness changes in the algorithm during the search process. Average fitness represents the average fitness value of all particles in the population, while the convergence curve represents the fitness trend of the optimal particle. As shown in Figure 7, the algorithm follows a search pattern of exploration followed by exploitation in all four test functions. But the convergence of the two curves is different. Average fitness, as a macroscopic representation of overall fitness, converges quickly in the initial stage due to the large differences between particles during initialization. As iterations progress, FBCA mainly performs local searches around the current optimal soldier particle, so the overall fitness value stabilizes after a sharp decrease. The convergence curve, on the other hand, shows a more detailed view of the fitness changes of the optimal particle, exhibiting a monotonically decreasing trend, indicating that FBCA continuously optimizes the quality of the solution. In summary, combined with the search history plot, it can be seen that FBCA not only accurately locates the optimal solution but also demonstrates good global convergence characteristics.

The trajectory history graph shows the trajectory of the optimal solution for the particle during the iteration process. The horizontal axis represents the number of iterations, and the vertical axis represents the change in the value of the optimal particle in the first dimension. The graph reflects the dynamic changes in the optimal solution during 500 iterations. The large fluctuations in the initial stage of FBCA indicate its ability to quickly explore the global region; as iterations progress, the algorithm exhibits differentiated search and convergence characteristics on different functions. For example, in Figure 7b, the algorithm completes extensive exploration and quickly locates the center of the multimodal region within about 150 iterations, and then approaches the optimal solution through fine exploitation; Figure 7d shows that exploration is the main activity during iterations 0 to 200, continuous exploitation is the stage from 200 to 400, and finally, it converges to the global optimum during iterations 400 to 500. This demonstrates that FBCA can adaptively and dynamically adjust the exploration and exploitation mechanisms to achieve an effective balance of exploration–exploitation.

### 4.3. Quantitative Analysis

**Figure 8:**
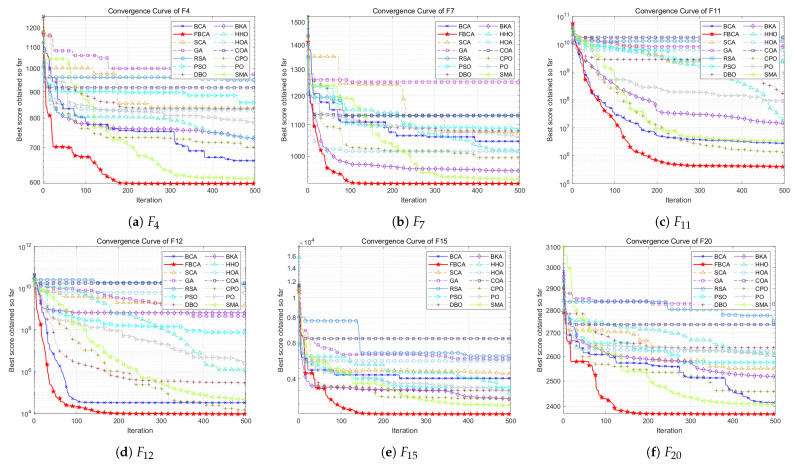
Convergence curve of the FBCA and its comparative algorithms with 30D.

**Figure 9:**
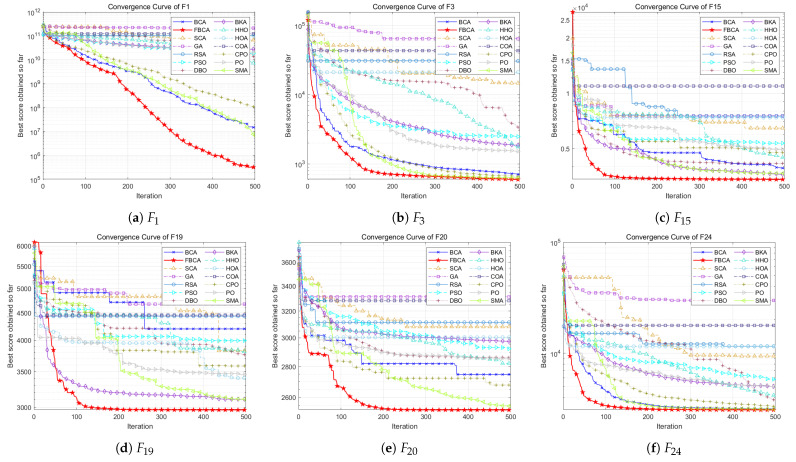
Convergence curve of the FBCA and its comparative algorithms with 50D.

**Figure 10:**
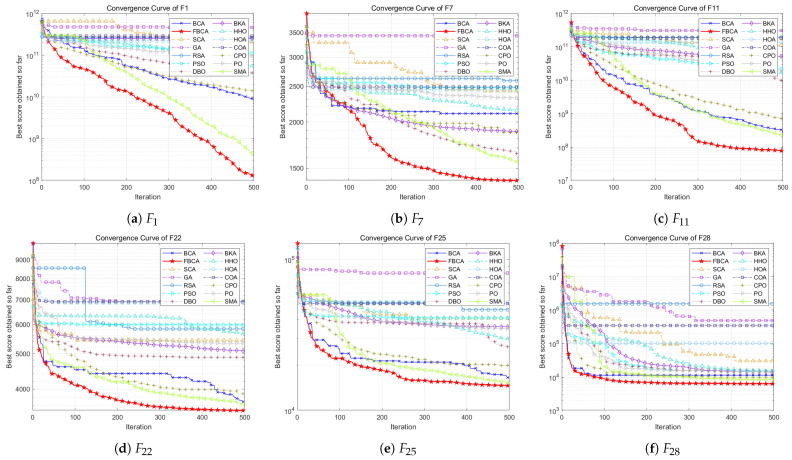
Convergence curve of the FBCA and its comparative algorithms with 100D.

**Figure 11:**
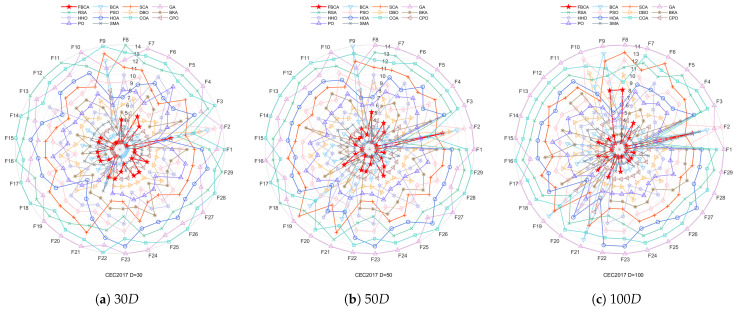
Radar chart of FBCA and other algorithms’ rankings.

This section shows the convergence curves in different dimensions, as shown in Figure 8, Figure 9 and Figure 10. Table 2 summarizes the comprehensive rankings of each algorithm in different dimensions. As shown in Table 3, we rank the algorithms on each test function one by one, their detailed mean and standard deviation data are listed in Table A1, Table A2, Table A3, and the best results of each function are marked with a gray background. At the same time, Figure 11 visualizes and compares the algorithm performance through radar maps. Finally, the experimental results show that FBCA has excellent performance in terms of convergence accuracy, avoidance of premature convergence, convergence speed, and exploration–exploitation balance ability.

In terms of convergence speed and convergence accuracy, Figure 8, Figure 9 and Figure 10 show that FBCA finds an optimal solution in almost every iteration from 0 to 500, demonstrating its excellent convergence speed. Comparing the final result of the $500_{th}$ iteration, as shown in Figure 9d, we can see that FBCA’s solution is optimal, and its convergence accuracy is significantly improved compared to BCA. Figure 9a,b also show that the optimal solution found is significantly superior to that of other algorithms, demonstrating that FBCA has excellent convergence accuracy.

In terms of avoiding premature convergence, as shown in Figure 8e and Figure 9e, the curve slope is generally small during the period from 0 to 100 iterations, that is, the downward trend is not obvious, but the curve suddenly drops rapidly when it tends to equilibrium, and a better solution is found. This shows that FBCA’s excellent exploration ability can get rid of the extreme value trap in time when the algorithm falls into the local optimum, thereby effectively avoiding premature convergence.

The exploration–exploitation balance is a crucial metric for evaluating an algorithm’s overall performance. From this perspective, the detailed and comprehensive rankings in Table 2 and Table 3 can be combined to assess FBCA’s exploration–exploitation capabilities. Composite functions are among the most complex types of functions in the IEEE CEC 2017 test suite, and their multimodal extreme value traps are well-suited for evaluating FBCA’s exploration–exploitation balance. Table 3 shows that FBCA’s winning the first place gradually increases in three dimensions. In the 100-dimensional problem, 6 out of 10 combination functions won first place, which fully indicates that FBCA has significantly improved its exploration–exploitation capabilities.

Further combined with Table 2, it can be seen that FBCA ranks first in the comprehensive ranking of all three dimensions. The radar map in Figure 11 also visually shows that FBCA is in the lead, with the smallest closed graphic area. This demonstrates FBCA’s excellent combined ability to balance exploration and exploitation and its excellent performance on the IEEE CEC 2017 test suite.

### 4.4. Statistical Testing

In this section, the Wilcoxon rank sum test was employed to evaluate the statistical significance of the algorithmic performance [42]. Specifically, for each benchmark function and dimensional setting, FBCA and the comparative algorithms were each executed 30 independent times, and the differences in their errors were ranked by sign. When the sum of positive ranks was significantly greater than that of the negative ranks (*p* < 0.05), the result was marked as “+”, indicating that FBCA performed significantly better than the compared algorithm. Conversely, a “–” sign indicated that FBCA performed significantly worse, while “=” denoted no statistically significant difference. The “(+/=/−)” column in Table 4 reports the win–tie–loss outcomes of this test under 30D, 50D, and 100D dimensions.

Experimental results demonstrate that FBCA significantly outperforms most compared algorithms. Across three dimensions, FBCA achieves a net win of more than 80 compared with most algorithms, demonstrating its robust performance across a wide range of problem sizes. Further comparisons with the original BCA and the high-performance algorithms SMA and CPO reveal that FBCA’s win rate steadily increases with increasing dimensionality. In particular, at 100 dimensions, FBCA achieves a significant win rate of 62% and 68% compared to BCA and CPO, respectively, demonstrating its significantly enhanced optimization capabilities for complex, high-dimensional problems.

### 4.5. Stability Analysis

**Figure 12:**
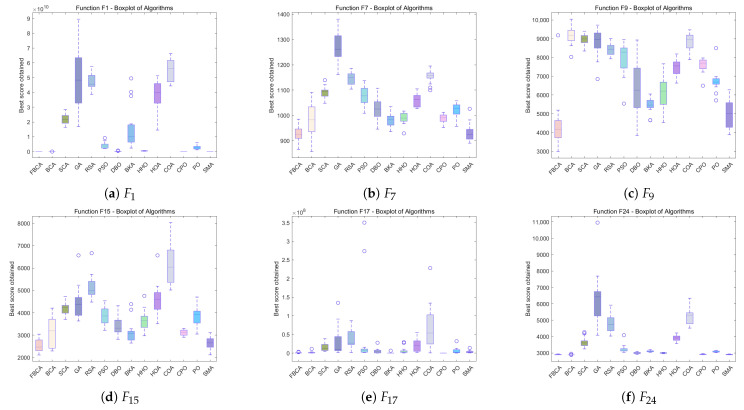
Boxplots of the FBCA and its comparative algorithms with 30D.

**Figure 13:**
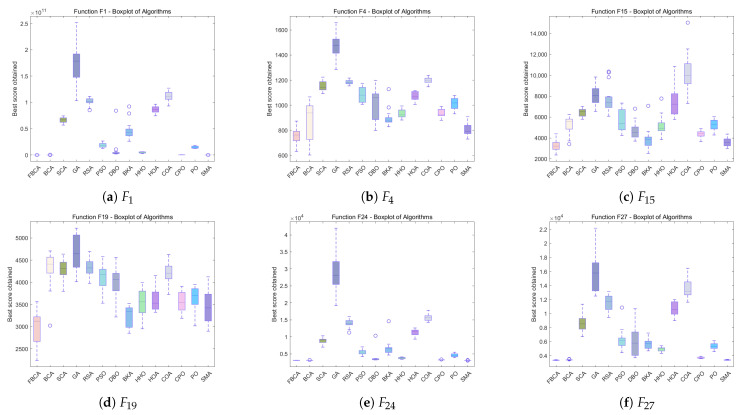
Boxplots of the FBCA and its comparative algorithms with 50D.

**Figure 14:**
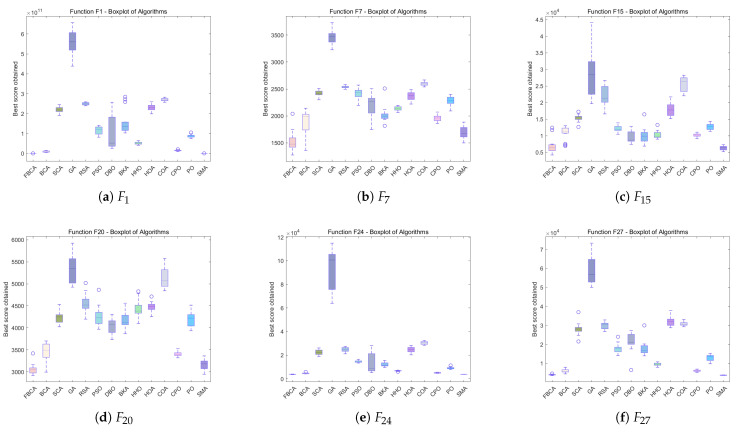
Boxplots of the FBCA and its comparative algorithms with 100D.

Figure 12, Figure 13 and Figure 14 show the boxplot results for all algorithms across various dimensions. The horizontal axis represents the compared algorithms, and the vertical axis represents the evaluation results of each algorithm on the test function. Each algorithm’s corresponding boxplot includes statistical features such as the median, quartiles, maximum, minimum, and outliers, comprehensively reflecting the distribution characteristics of the algorithm’s performance [43]. As can be seen from the figures, FBCA demonstrates excellent performance in both result stability and robustness.

From the perspective of the robustness of FBCA, as shown in Figure 12, Figure 13 and Figure 14, it can be observed that the overall results of FBCA are generally at a lower level than other algorithms on the three test functions of F1, F15 and F24, and the upper and lower boundary spacing of the box plot is small, indicating that the result fluctuation is small and the stability is high. Especially on the F1 function, the value of results are almost stable around 0, as shown in Figure 12a and Figure 13a, which further verify that FBCA has good robustness in different dimensions. In addition, FBCA achieves excellent performance results in all three dimensions and four types of functions covered by the same dimension. This shows that the algorithm not only has cross-dimensional robustness but also shows good cross-function robustness.

From the perspective of the stability performance of FBCA, with the increase in dimensions, the optimization results of FBCA are more significant when dealing with hybrid functions and composite functions with higher diversity and complexity, as shown in Figure 13e and Figure 14f. This shows that FBCA has the ability to jump out of the local optimal and find a better global solution. At the same time, it can still maintain high stability on these complex and diverse functions, indicating that the algorithm not only has good stability, but also verifies that the exploration and exploitation strategy proposed in this paper has been effectively played, so that the balance between exploration and exploitation of FBCA has been improved.

### 4.6. Parameter Sensitivity and Mechanism Validation

In order to verify the rationality of FBCA, this section presents the parameter sensitivity analysis, ablation experiments, and performance comparisons with SOTA algorithms. As is well known, the IEEE CEC 2017 test suites become more difficult to optimize as the dimensions increase. Therefore, this paper selects 29 functions of dimension 100 from these test suites as evaluation benchmarks. In terms of experimental design, Section 4.6.1 conducts sensitivity analysis on changes in the probability parameter $p_{1}$; Section 4.6.2 examines the impact of different Gaussian perturbation terms N on algorithm performance; Section 4.6.3 conducts ablation experiments by replacing the spiral perturbation mechanism; Section 4.6.4 compares the performance differences between the original PSO velocity update method and the cognitively driven velocity update strategy in FBCA. Finally, Section 4.6.5 provides a comprehensive performance comparison with SaDE [44], L-SHADE [45], and L-SHADE_EpSin [46] algorithms.

#### 4.6.1. Influence of the Probability Parameter $p_{1}$

**Figure 15:**
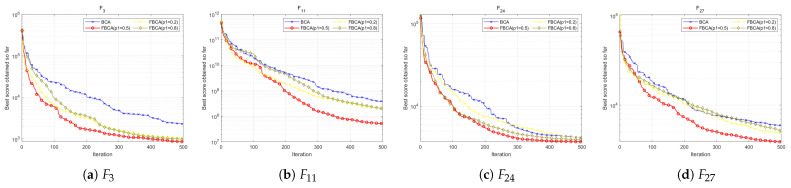
Convergence curve of the BCA, FBCA ($p_{1}$ = 0.2), FBCA ($p_{1}$ = 0.5) and FBCA ($p_{1}$ = 0.8).

To verify the rationality of the parameter $p_{1}$ setting, we conducted parameter sensitivity analysis for different values of $p_{1}$. Algorithms involved in the comparison included BCA, FBCA ($p_{1}$ = 0.2), FBCA ($p_{1}$ = 0.5), and FBCA ($p_{1}$ = 0.8). As shown in the Table 5, when $p_{1}$ = 0.5, the algorithm performs best out of 29 test functions, ranking first. Further analysis revealed that FBCA with $p_{1}$ = 0.2 performs exceptionally well on composite functions, achieving first place in 6 out of 10 such functions; while FBCA with $p_{1}$ = 0.8 exhibits better convergence performance on unimodal functions. Comparatively, FBCA with $p_{1}$ = 0.5 demonstrates stronger versatility and adaptability. The results show that FBCA ($p_{1}$ = 0.5) achieves optimal results on three multimodal functions, two mixed functions, and four combined functions. Figure 15 shows the convergence curves on the multimodal function $F_{3}$, the hybrid function $F_{11}$, and the composite functions $F_{24}$ and $F_{27}$. Overall, setting $p_{1}$ = 0.5 enables FBCA to demonstrate excellent comprehensive adaptability in various types of optimization functions.

#### 4.6.2. Effect of Gaussian Perturbation Variance

**Figure 16:**
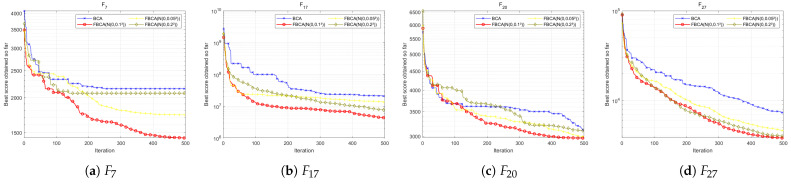
Convergence curve of the FBCA with variations in the Gaussian item N.

To further verify the rationality of the Gaussian perturbation term parameter N, we compared FBCA versions using $N(0,0.05^{2})$, $N(0,0.1^{2})$ and $N(0,0.2^{2})$. As shown in the Table 6, the $N(0,0.1^{2})$ configuration exhibits the best overall performance, especially in hybrid and composite functions containing numerous local extremum traps. As shown in Figure 16b, the FBCA with the $N(0,0.1^{2})$ configuration converges significantly faster and with higher accuracy. Although the FBCA($N(0,0.05^{2})$) and FBCA($N(0,0.2^{2})$) also achieved some results on different functions, they did not show significant optimal characteristics in any type of function. In summary, when the Gaussian perturbation term is set to $N(0,0.1^{2})$, FBCA not only achieves the best overall optimization performance but also demonstrates good adaptability to complex functions.

#### 4.6.3. Comparison of Spiral Perturbation Mechanisms

To verify the effectiveness of the proposed exponentially modulated spiral perturbation mechanism, we designed ablation experiments for four spiral mechanisms, named $Spiral_{1}$ to $Spiral_{4}$. $Spiral_{1}$ and $Spiral_{2}$ are two variants of the logarithmic spiral: $Spiral_{1}$ corresponds to the proposed mechanism with a dynamic exponential parameter *b*, while $Spiral_{2}$ has a fixed *b* = 1; $Spiral_{3}$ is the Archimedean Spiral, and $Spiral_{4}$ is the Rose Spiral.

(20) $$\left\{\begin{array}{cc} \psi =rand\cdot 4\pi & \\ linear\_term=c+d_{arch}\cdot \psi & \\ Arch\_factor=linear\_term\cdot \cos(\psi ) \end{array}\right.$$

The mathematical formula for the Archimedean Spiral is defined in Equation (20), where *c* = 0.5 and $d_{arch}$ = 0.3. The mathematical formula for Rose Spiral is defined in Equation (21), where $e_{rose}$ = 1.2, *n* = 3. In Equations (20) and (21), rand is a random value that takes values in the range [0, 1].

(21) $$\left\{\begin{array}{cc} k\left(t\right)=1+\frac{0.3t}{Max\_Gen} & \\ \xi =rand\cdot 2\pi \cdot k(t) & \\ Rose\_factor=e_{rose}\cdot \cos\left(n\cdot \xi \right)\cdot \cos\left(\xi \right) \end{array}\right.$$

**Figure 17:**
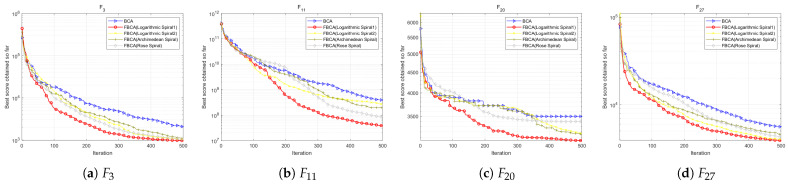
Convergence curve of the FBCA with various spiral mechanisms.

As shown in the Table 7, the proposed $Spiral_{1}$ has the best optimization performance, with an average ranking of 2.28, leading the second-place mechanism by 0.31. As shown in Figure 17b,c, the FBCA (red curve) using exponentially modulated spiral perturbation significantly outperforms other mechanisms in global convergence. In detail, the Archimedean Spiral has a certain advantage in the composite function, while the Rose spiral exhibits relatively stable performance and lacks obvious characteristics. Comparing the results of the two logarithmic spirals, it can be seen that although $Spiral_{1}$ did not achieve the optimal solution on unimodal functions, it outperformed $Spiral_{2}$ on the other three types of functions, especially showing a more outstanding optimization ability on hybrid functions.

#### 4.6.4. Validation of the Velocity Update Mechanism

**Figure 18:**
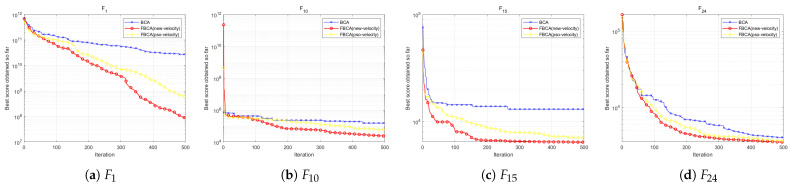
Convergence curve of the BCA, FBCA (new-velocity) and FBCA (pso-velocity).

To verify the difference between the cognitive-driven velocity update mechanism and the original PSO velocity update mechanism, we tested FBCA under both mechanisms, named FBCA (pso-velocity) and FBCA (new-velocity), respectively. In the Table 8, $velocity_{1}$ is FBCA (new-velocity) and $velocity_{2}$ is FBCA (pso-velocity). Experimental results show that the improved velocity update strategy significantly enhances the quality of solution. As shown in Figure 18, FBCA (new-velocity) achieves better results on functions $F_{1}$, $F_{10}$, $F_{15}$, and $F_{24}$, demonstrating stronger robustness. Particularly in the $F_{1}$ function of Figure 18a, the convergence curve of FBCA (new-velocity) is significantly better than that of FBCA (pso-velocity). Overall, FBCA (new-velocity) achieved first place in 15 out of 29 test functions, while FBCA (pso-velocity) only performed the best in seven functions, validating the effectiveness of the improved mechanism.

#### 4.6.5. Comparison with SOTA Optimizers

**Figure 19:**
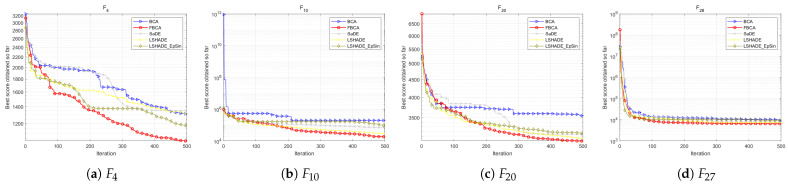
Convergence curve of the FBCA and other SOTA optimizers.

To verify the leading performance of our proposed FBCA, we compared it with SaDE, L-SHADE, and L-SHADE_EpSin cutting-edge algorithms. Looking at the rankings of the algorithms across the four function types, SaDE showed no significant advantage. L-SHADE_EpSin excels at optimizing hybrid functions, achieving a strong 12 first-place results. However, L-SHADE also has some limitations. While FBCA only achieved seven first-place results, further analysis reveals its relative superiority. For example, in Figure 19a, L-SHADE exhibits significantly weaker optimization capabilities on F4, while FBCA easily outperforms L-SHADE, SaDE, and L-SHADE_EpSin, achieving first place. Furthermore, although FBCA did not achieve first place on $F_{13}$, $F_{16}$, and $F_{22}$, its second-place ranking still significantly surpassed the fourth-place L-SHADE. Therefore, as shown in the Table 9, FBCA ultimately achieved the overall first-place ranking.

## 5. MLP Optimization Problems

In this section, we evaluate and validate the feasibility of the FBCA algorithm using six MLP optimization problems, including three classification problems and three function approximation problems. First, Section 5.1 introduces how to train an MLP using FBCA. Subsequently, Section 5.2, Section 5.3, Section 5.4 present experimental results and analysis for the three classification problems: MLP_XOR, MLP_Iris and MLP_Heart. Section 5.5, Section 5.6Section 5.7 demonstrate the optimization performance for the three function approximation problems: MLP_Sigmoid, MLP_Cosine and MLP_Sine. Section 5.8 compares the experimental results of FBCA and gradient-based optimizers. Section 5.9 discusses the performance of FBCA on MLP optimization problems, limitations, and future work.

### 5.1. Training MLPs Using FBCA

**Figure 20:**
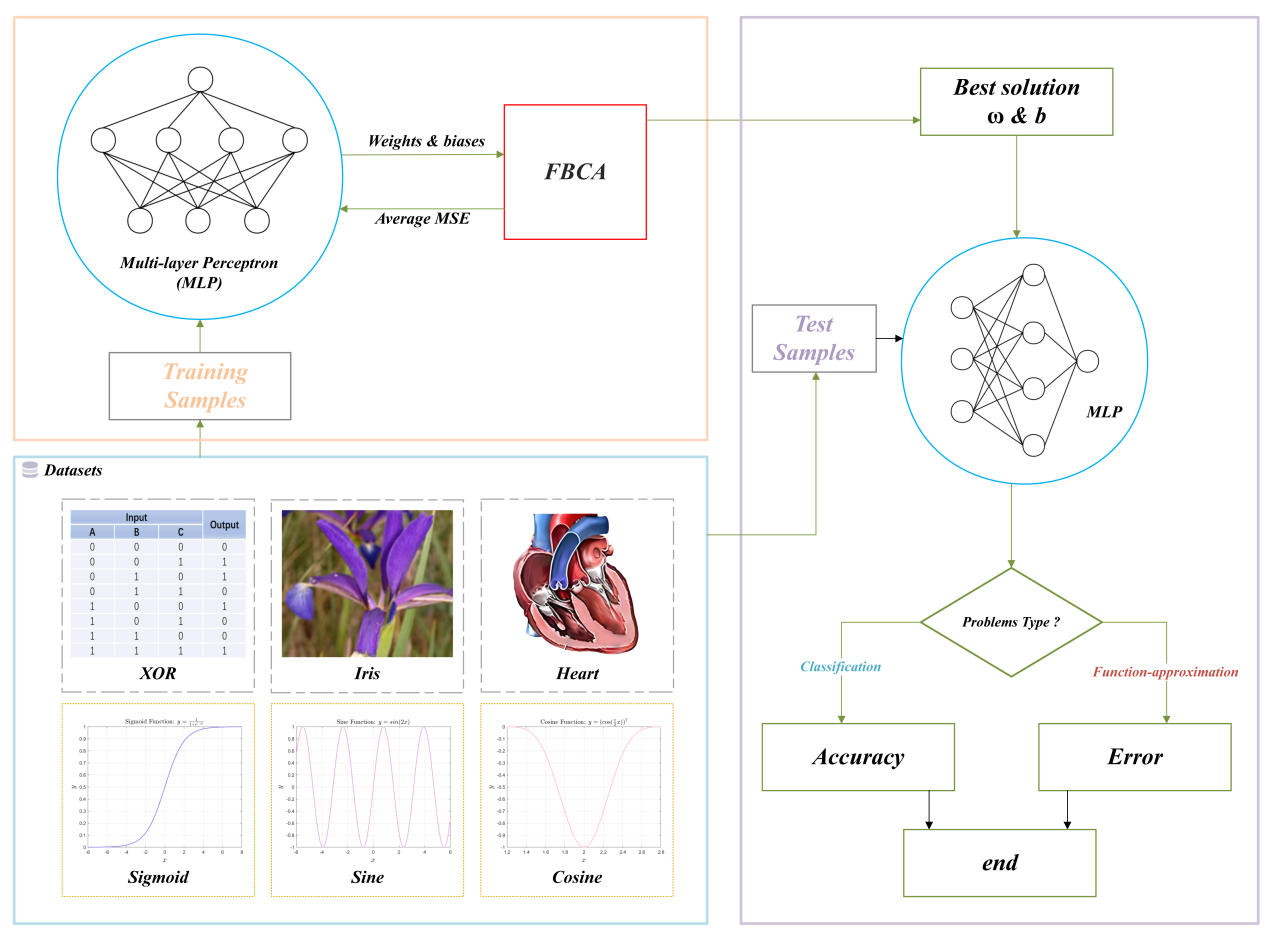
The FBCA−MLP training model.

Figure 20 shows an optimization model that combines FBCA with MLP, referred to as FBCA-MLP. In this model, FBCA is used to optimize the weight and bias parameters of the MLP to minimize the mean squared error (MSE) on the training dataset.

Specifically, the training process begins by preparing training samples and test samples. The training samples are then fed into the FBCA−MLP model, where iterative training searches for optimal weight and bias parameters. Once the optimal parameters are obtained, they are applied to the MLP and evaluated on the test samples. Depending on the problem type, the model’s accuracy or test error is ultimately output.

Before applying FBCA to the MLP optimization problem, we have detailed definitions of the relevant parameters required for training, as shown in Table 10 and Table 11. The table clearly shows the number of training samples, test samples, MLP structure, and problem dimensions for two different types of problems.

According to the No Free Lunch Theorem (NFL) [47], there is no universal algorithm that can perform best on all problems, which has become an academic consensus. Accordingly, this paper compares the experimental results of eight cutting-edge algorithms, including FBCA and SMA, Guided Learning Strategy (GLS) [48], and the Osprey Optimization Algorithm (OOA) [49], on three MLP classification optimization problems and three function approximation problems, to verify their respective areas of strength [50].

### 5.2. MLP_XOR Problem

This study uses a three-bit XOR dataset [51], with input features as three-bit binary numbers and output results as the parity check values of these three features. The comparative results in Table 12 show that the classification accuracy of FBCA not only reached 100%, but also, from the mean results of MSE, the optimization results of FBCA are in the order of $10^{-6}$. While other compared algorithms only reached $10^{-1}$∼$10^{-3}$, the optimization performance was improved by about three to five orders of magnitude. This indicates that FBCA demonstrates strong exploration capabilities in MLP optimization.

### 5.3. MLP_Iris Problem

The Iris dataset is for a three-class classification problem [52,53], with its four input features being sepal length, sepal width, petal length, and petal width, and the output results corresponding to the three iris flower categories. As shown in Table 13, the mean MSE of FBCA performs the best. Meanwhile, the MLP model optimized by FBCA also ranks first in the best accuracy in prediction, indicating that compared to other algorithms, FBCA has superior global convergence ability.

### 5.4. MLP_Heart Problem

Compared with the previous two classification problems, the dimension of the Heart dataset [54,55] has increased from 10 to 1000, an increase of about 10^2^, and the number of input features has also increased from 3 or 4 to 22, making FBCA face higher-dimensional and more complex optimization challenges. From the results of Table 14, it can be seen that the mean of MSE of other algorithms is in the order of $10^{-1}$, while the mean of FBCA reaches $10^{-2}$, showing a new breakthrough in the order of magnitude. At the same time, its accuracy rate is also the first, indicating that FBCA can balance exploration and exploitation to find a better solution.

### 5.5. MLP_Sigmoid Problem

The Sigmoid dataset is the simplest function approximation problem in this article. Its specific expression is listed in Table 11, along with the mathematical expressions for other function approximation problems [50]. Unlike classification problems, function approximation problems are evaluated not by accuracy but by test error. Table 15 shows that FBCA achieves the best performance in both mean and test error. In particular, the test error result shows a 0.8 reduction for FBCA compared to BCA, demonstrating a significant improvement in FBCA’s optimization performance for the Sigmoid problem.

### 5.6. MLP_Cosine Problem

The optimization difficulty for the Cosine dataset is higher than that for the Sigmoid problem. As shown in Table 16, FBCA maintains the best performance among the compared algorithms, demonstrating its ability to achieve higher-precision solutions during exploitation. Furthermore, FBCA achieves the lowest test error, surpassing not only BCA but also GLS, SMA, and HHO, all of which have lower test errors than BCA. This result further highlights the significant improvements and advantages of FBCA in function approximation.

### 5.7. MLP_Sine Problem

The Sine dataset is the most complex function approximation problem in this paper. However, the MLP optimization model based on FBCA maintains stable performance and significantly improves performance. As shown in Table 17, FBCA achieves the best mean MSE metric and the lowest test error. This demonstrates that FBCA effectively avoids falling into local optima and finds more suitable weight and bias parameters for MLP training.

### 5.8. Comparison with Gradient-Based Optimizers

In the experiments in Section 5.2, Section 5.3, Section 5.4, Section 5.5, Section 5.6, Section 5.7, we employed various metaheuristic algorithms as optimizers to adjust the weights and biases of the MLP. To further verify the effectiveness and persuasiveness of this optimization approach, this section selects a representative dataset each from the classification problems and the function approximation problems, and compares it with four traditional gradient-based optimization algorithms [56]. These four optimization algorithms are SGD [57], Adam [58], RMSprop [59], and Adagrad [60].

In the classification problem, we chose the MLP_Heart dataset for our experiments. This dataset has a high dimension, and the MLP model structure is relatively complex, making it a challenging task to optimize. In the function approximation problem, we selected MLP_Sine, which is one of the most difficult test functions to converge among similar problems.

As shown in the Table 18, the proposed FBCA can more effectively adjust weights and biases during MLP training, enabling the model to achieve better generalization performance. Compared with four traditional gradient optimizers, FBCA achieves the highest classification accuracy and the smallest test error on both problems, verifying its significant role in improving MLP performance within a non-gradient optimization framework.

### 5.9. Discussion

To address the issue of nonlinear MLPs easily getting trapped in saddle points and local optima in nonconvex optimization, we not only systematically evaluated the optimization capability of FBCA using the IEEE CEC 2017 benchmark function but also validated it using six MLP optimization problems. FBCA’s hierarchical structure, perturbation updates, and velocity-driven search fully leverage its global optimization capabilities, enabling it to achieve more stable convergence and lower errors in small-to-medium-scale MLP tasks, demonstrating its effectiveness in nonconvex optimization scenarios for nonlinear MLPs. This study systematically verifies the role of the improved FBCA mechanism in enhancing search balance and convergence performance through parameter sensitivity analysis, ablation experiments, and complexity comparison. These results collectively support FBCA’s cutting-edge advantage in six MLP optimization problems. Moreover, FBCA not only achieved first place in metaheuristic algorithms, but also demonstrated a representative lead compared to traditional metaheuristic algorithms and mainstream gradient optimizers. It showed outstanding performance in the most complex MLP_Heart classification problem and MLP_Sine problem. Not only did it outperform Adam and RMSprop by more than 10% in MLP_Heart accuracy but it also reduced the test error by more than 10 compared to SGD and Adagrad in MLP_Sine.

While this paper mentions the potential applicability of FBCA in deep networks such as CNNs and Transformers, it does not provide corresponding empirical analysis and experimental verification. This limitation stems primarily from the fact that this study focuses on the weight and bias optimization of MLPs without directly comparing the optimization performance of other deep learning. However, as a global optimization framework, FBCA possesses scalability for adapting to different network depths due to its hierarchical population–army–soldier particle generation and global optimization mechanism. For CNNs, FBCA can effectively model the convolutional kernel structure through block-level parameter mapping and weight sharing constraints. Specifically, each convolutional kernel can be regarded as an army, with multiple soldiers within it performing searches in local parameter subspaces; during the search process, the updated sub-blocks of the soldiers can synchronously act on the shared weight structure, thereby achieving the overall optimization of the convolutional kernel. For Transformer architectures, FBCA can be combined with its multi-head self-attention mechanism to design a parallel evolutionary process. Each attention head can be regarded as a relatively independent optimization subspace, corresponding to an independent army in FBCA. The overall fitness is determined by the joint performance of all attention heads, ensuring global coordination and consistency in the distribution of multi-head attention. In summary, FBCA provides a new theoretical perspective and research direction for the generalization of metaheuristic algorithms in complex deep networks such as CNNs and Transformers, laying the foundation for future optimization methods in structured parameter spaces.

Meanwhile, due to computational limitations, this study did not test large-scale datasets and deep MLP architectures; therefore, its performance and scalability in these scenarios remain to be verified. Future work will focus on the performance of FBCA in high-dimensional neural network parameter spaces, and evaluate its hybrid optimization strategy combined with gradient methods, further exploring its application potential in complex deep learning models.

## 6. Conclusions and Future Work

The proposed FBCA integrates soft Gaussian perturbation asymmetry, adaptively modulated spiral perturbation factors, and dynamically decreasing nonlinear cognitive coefficients, enabling the algorithm to achieve rapid detection, fast adaptation, and fast convergence. This demonstrates the algorithm’s flexibility and dynamic control during the search process. Its outstanding performance on the IEEE CEC 2017 benchmark suite, particularly in high-dimensional and complex composite optimization problems, demonstrates that FBCA not only maintains stable convergence speed and high optimization accuracy but also achieves notable improvements in six MLP optimization problems. These results validate the algorithm’s robustness and generalization ability in high-dimensional nonconvex optimization scenarios. More importantly, FBCA flexibly controls the proposed mechanism through refined binary gates, achieving an adaptive exploration–exploitation balance search, demonstrating a novel design approach for swarm intelligence algorithms that emphasizes structural flexibility and search balance.

Although FBCA demonstrates strong performance across multiple problems, several promising directions remain for future research. Theoretically, developing a multi-objective extension of FBCA could enhance its adaptability to problems involving multiple or conflicting constraints. Additionally, integrating strategic military decision-making models may inspire interdisciplinary advancements in swarm-based intelligent optimization. The population initialization strategy also warrants further exploration—approaches such as latin hypercube sampling, good-point sets, or customized initialization schemes could improve search space coverage and convergence behavior. At the algorithmic structural level, future work could focus on novel exploration–exploitation balance mechanisms to address the global convergence limitations of the original BCA. Finally, from an application perspective, FBCA’s performance in MLP optimization lays the foundation for its broader neural network field, and its application potential in complex intelligent tasks such as medical image analysis [61], financial timing prediction [62], and natural language emotion recognition [63] can be further explored in the future.

Finally, we have made BCA-related research materials publicly available at www.jianhuajiang.com, accessed on 1 January 2025, and we welcome researchers interested in exploring and studying the theoretical innovations and migration applications of BCA and FBCA.

## Figures and Tables

**Table 1:** Parameter settings for each algorithm.

Algorithms	Parameters	Values	Reference
SCA	*a*	2	[31]
	*r*	Linearly decreased from a to 0	
GA	CrossPercent	70%	[22]
	MutatPercent	20%	
	ElitPercent	10%	
RSA	Evolutionary sense	randomly decreasing values between 2 and −2	[32]
	Sensitive parameter	β = 0.005	
	Sensitive parameter	α = 0.1	
PSO	Cognitive component	2	[24]
	Social component	2	
DBO	k and λ	0.1	[33]
	b	0.3	
	S	0.5	
BKA	*p*	0.9	[34]
	*r*	range from [0, 1]	
HHO	$E_{0}$	range from [−1, 1]	[35]
	β	1.5π	
HOA	Angle of inclination of the trail	range from [0, 50°]	[36]
	Sweep Factor (SF) of the hiker	range from [1, 3]	
COA	*I*	1 or 2	[37]
CPO	α	0.1	[38]
	$T_{f}$	0.5	
	*T*	2	
PO	*p*	[0, 1]	[39]
SMA	$v_{c}$	Linearly decreased from 1 to 0	[40]

**Table 2:** The overall ranking results of FA-BCA and other algorithms.

	Index	FBCA	BCA	SCA	GA	RSA	PSO	DBO	BKA	HHO	HOA	COA	CPO	PO	SMA
D = 30	Average ranking	**2.72**	3.10	9.72	12.93	12.28	8.90	5.79	6.24	7.34	10.10	13.10	2.79	7.14	2.83
	Total ranking	1	4	10	13	12	9	5	6	8	11	14	2	8	3
D = 50	Average ranking	**2.45**	3.86	9.97	13.31	11.86	8.93	6.17	6	6.52	10.10	12.79	3.41	7.07	2.55
	Total ranking	1	4	10	14	12	9	6	5	7	11	13	3	8	2
D = 100	Average ranking	**2.52**	4.55	10.10	13.90	11.17	9.24	6.24	6.03	5.93	9.79	12.17	4	6.69	2.66
	Total ranking	1	4	11	14	12	9	7	6	5	10	13	3	8	2

The bold part indicates the optimal result.

**Table 3:** The detailed ranking results of all algorithms on CEC2017 test functions.

F	FBCA			BCA			SCA			GA			RSA			PSO			DBO			BKA			HHO			HOA			COA			CPO			PO			SMA		
	30	50	100	30	50	100	30	50	100	30	50	100	30	50	100	30	50	100	30	50	100	30	50	100	30	50	100	30	50	100	30	50	100	30	50	100	30	50	100	30	50	100
F1	2	1	1	1	3	3	10	10	10	13	14	14	12	12	12	8	8	8	5	6	6	9	9	9	6	5	5	11	11	11	14	13	13	4	4	4	7	7	7	3	2	2
F2	7	10	10	12	12	12	9	9	9	14	14	13	8	4	3	13	13	14	11	11	8	2	1	1	3	3	6	6	2	2	10	8	4	4	5	7	5	7	5	1	6	11
F3	1	1	1	2	2	3	10	10	10	12	14	14	13	12	12	8	8	8	5	5	7	9	9	9	6	6	5	11	11	11	14	13	13	4	4	4	7	7	6	3	3	2
F4	1	1	1	3	3	4	11	11	11	14	14	14	13	12	12	10	10	10	6	7	7	5	4	3	7	6	6	9	9	9	12	13	13	4	5	5	8	8	8	2	2	2
F5	4	3	2	1	1	1	8	10	11	14	14	14	13	12	13	6	7	10	5	5	6	7	6	5	10	8	7	9	11	9	12	13	12	2	2	3	11	9	8	3	4	4
F6	5	4	4	3	3	3	9	11	13	14	14	14	12	12	11	6	6	8	4	5	5	8	7	6	11	10	10	10	9	9	13	13	12	2	2	1	7	8	7	1	1	2
F7	1	1	1	3	3	3	11	11	11	14	14	14	12	13	12	10	9	10	7	7	7	4	6	5	6	5	6	9	10	9	13	12	13	5	4	4	8	8	8	2	2	2
F8	4	5	8	2	1	3	11	11	13	12	14	14	14	13	11	9	8	12	6	9	10	5	4	2	10	10	7	7	7	6	13	12	9	1	2	4	8	6	5	3	3	1
F9	1	2	8	12	14	13	13	12	12	11	11	14	10	10	10	9	9	9	5	5	6	3	3	2	4	4	3	7	7	5	14	13	11	8	8	7	6	6	4	2	1	1
F10	1	3	2	2	4	9	9	9	7	14	14	14	12	12	10	10	11	13	7	6	11	6	7	3	5	5	5	11	10	8	13	13	12	3	2	4	8	8	6	4	1	1
F11	1	2	1	2	1	3	10	10	10	12	12	14	14	13	12	9	8	8	5	5	5	8	9	9	6	6	6	11	11	11	13	14	13	3	3	4	7	7	7	4	4	2
F12	1	2	2	2	1	1	10	10	10	12	12	14	14	13	12	9	9	8	6	6	6	8	8	9	5	5	5	11	11	11	13	14	13	3	3	3	7	7	7	4	4	4
F13	3	3	1	5	4	5	8	9	11	13	13	14	14	12	12	10	10	9	6	6	7	2	2	2	11	8	6	9	11	10	12	14	13	1	1	3	7	7	8	4	5	4
F14	3	2	3	2	1	1	9	10	10	12	13	14	13	12	12	10	8	9	5	7	6	6	9	8	7	5	5	11	11	11	14	14	13	1	3	2	8	6	7	4	4	4
F15	1	1	1	5	5	7	10	10	10	11	12	14	13	13	12	9	9	8	6	6	3	4	3	6	8	7	5	12	11	11	14	14	13	3	4	4	7	8	9	2	2	2
F16	3	1	1	2	6	4	10	11	9	12	14	12	14	13	13	6	9	8	8	8	6	5	4	10	7	5	5	11	10	11	13	12	14	1	3	3	9	7	7	4	2	2
F17	3	2	3	5	5	6	9	10	11	13	12	14	12	13	12	10	8	9	6	7	8	2	3	2	7	6	4	11	11	10	14	14	13	1	1	1	8	9	7	4	4	5
F18	3	4	2	2	1	1	11	10	10	12	13	14	13	14	12	9	9	8	6	6	6	7	7	9	5	5	5	10	11	11	14	12	13	1	2	3	8	8	7	4	3	4
F19	2	1	4	4	11	13	11	13	12	14	14	14	13	12	9	10	9	10	8	8	7	5	2	2	9	5	3	6	7	6	12	10	11	1	4	8	7	6	5	3	3	1
F20	1	1	1	3	3	4	11	9	7	14	14	14	12	12	12	10	8	9	6	5	5	7	6	6	9	11	10	8	10	11	13	13	13	4	4	3	5	7	8	2	2	2
F21	4	3	4	5	10	13	12	12	11	14	14	14	11	13	10	9	8	8	3	6	5	7	2	2	8	5	3	10	9	7	13	11	12	1	4	9	2	7	6	6	1	1
F22	4	3	1	2	2	2	7	7	8	13	14	14	11	10	9	10	11	11	5	5	5	8	8	7	9	9	10	12	12	13	14	13	12	3	4	4	6	6	6	1	1	3
F23	3	2	3	2	4	2	7	7	8	14	14	14	9	9	10	11	11	11	5	5	5	8	8	7	10	10	9	13	13	13	12	12	12	4	3	4	6	6	6	1	1	1
F24	2	1	1	1	3	3	10	10	10	14	14	14	12	12	12	9	8	9	5	6	7	8	9	8	6	5	5	11	11	11	13	13	13	4	4	4	7	7	6	3	2	2
F25	4	4	1	1	2	3	8	10	10	13	14	14	12	12	12	6	9	9	5	5	5	10	8	8	9	7	6	11	11	11	14	13	13	3	3	4	7	6	7	2	1	2
F26	4	3	1	1	1	2	8	8	10	13	12	14	11	11	11	9	9	9	5	5	5	6	7	7	10	10	8	12	13	12	14	14	13	3	4	4	7	6	6	2	2	3
F27	4	2	2	3	3	4	10	10	10	14	14	14	12	12	11	9	8	8	7	7	7	8	9	9	5	5	5	11	11	13	13	13	12	1	4	3	6	6	6	2	1	1
F28	4	1	1	1	2	3	10	9	9	12	12	14	13	13	12	6	10	7	5	5	5	7	8	10	8	6	6	11	11	11	14	14	13	2	4	4	9	7	8	3	3	2
F29	2	2	2	1	1	1	10	10	10	11	12	14	14	13	13	8	9	8	5	5	5	7	6	9	6	7	6	12	11	11	13	14	12	4	3	3	9	8	7	3	4	4

**Table 4:** Results of the Wilcoxon rank sum test of FBCA and other algorithms.

Algorithms	D = 30	D = 50	D = 100	Total
	(+/=/−)	(+/=/−)	(+/=/−)	(+/=/−)
FBCA vs. BCA	7/16/6	13/8/8	18/6/5	38/30/19
FBCA vs. SCA	28/1/0	28/1/0	28/0/1	84/2/1
FBCA vs. GA	29/0/0	29/0/0	29/0/0	87/0/0
FBCA vs. RSA	28/1/0	28/0/1	27/1/1	83/2/2
FBCA vs. PSO	29/0/0	29/0/0	27/2/0	85/2/0
FBCA vs. DBO	25/3/1	27/2/0	26/2/1	78/7/2
FBCA vs. BKA	26/0/3	23/3/3	22/1/6	71/4/12
FBCA vs. HHO	28/0/1	28/0/1	24/2/3	80/2/5
FBCA vs. HOA	28/0/1	28/0/1	24/3/2	80/3/4
FBCA vs. COA	28/1/0	28/0/1	28/0/1	84/1/2
FBCA vs. CPO	12/6/11	17/6/6	20/5/4	49/17/21
FBCA vs. PO	27/1/1	28/0/1	24/3/2	79/4/4
FBCA vs. SMA	11/12/6	10/13/6	15/7/7	36/32/19

**Table 5:** Results of FBCA with various $p_{1}$ values.

F	BCA	FBCA ($p_{1}=0.2$)	FBCA ($p_{1}=0.5$)	FBCA ($p_{1}=0.8$)
F1	8.63 $\times \;10^{9}$	1.21 $\times \;10^{9}$	2.39 $\times \;10^{8}$	$1.92\times 10^{8}$
F2	8.22 $\times \;10^{5}$	6.76 $\times \;10^{5}$	7.20 $\times \;10^{5}$	$6.63\times 10^{5}$
F3	1.76 $\times \;10^{3}$	1.16 $\times \;10^{3}$	$1.04\times 10^{3}$	1.07 $\times \;10^{3}$
F4	1.63 $\times \;10^{3}$	1.30 $\times \;10^{3}$	$1.29\times 10^{3}$	1.32 $\times \;10^{3}$
F5	$6.33\times 10^{2}$	6.37 $\times \;10^{2}$	6.40 $\times \;10^{2}$	6.48 $\times \;10^{2}$
F6	$2.55\times 10^{3}$	2.67 $\times \;10^{3}$	2.75 $\times \;10^{3}$	3.31 $\times \;10^{3}$
F7	1.83 $\times \;10^{3}$	1.56 $\times \;10^{3}$	$1.53\times 10^{3}$	1.62 $\times \;10^{3}$
F8	$4.69\times 10^{4}$	6.88 $\times \;10^{4}$	7.71 $\times \;10^{4}$	8.82 $\times \;10^{4}$
F9	3.36 $\times \;10^{4}$	3.03 $\times \;10^{4}$	3.05 $\times \;10^{4}$	$2.50\times 10^{4}$
F10	1.94 $\times \;10^{5}$	6.24 $\times \;10^{4}$	5.70 $\times \;10^{4}$	$4.83\times 10^{4}$
F11	5.23 $\times \;10^{8}$	$1.09\times 10^{8}$	9.65 $\times \;10^{7}$	1.38 $\times \;10^{8}$
F12	$1.24\times 10^{4}$	3.18 $\times \;10^{4}$	5.12 $\times \;10^{4}$	1.89 $\times \;10^{5}$
F13	5.34 $\times \;10^{6}$	3.88 $\times \;10^{6}$	$3.03\times 10^{6}$	3.35 $\times \;10^{6}$
F14	1.80 $\times \;10^{4}$	$6.65\times 10^{3}$	2.25 $\times \;10^{4}$	2.66 $\times \;10^{4}$
F15	1.13 $\times \;10^{4}$	7.83 $\times \;10^{3}$	6.44 $\times \;10^{3}$	$6.23\times 10^{3}$
F16	8.20 $\times \;10^{3}$	6.15 $\times \;10^{3}$	$5.05\times 10^{3}$	5.12 $\times \;10^{3}$
F17	1.59 $\times \;10^{7}$	9.53 $\times \;10^{6}$	8.04 $\times \;10^{6}$	$4.62\times 10^{6}$
F18	$7.62\times 10^{3}$	1.16 $\times \;10^{4}$	1.21 $\times \;10^{4}$	4.32 $\times \;10^{4}$
F19	8.28 $\times \;10^{3}$	7.16 $\times \;10^{3}$	7.04 $\times \;10^{3}$	$6.93\times 10^{3}$
F20	3.43 $\times \;10^{3}$	3.12 $\times \;10^{3}$	$3.11\times 10^{3}$	3.17 $\times \;10^{3}$
F21	3.60 $\times \;10^{4}$	3.31 $\times \;10^{4}$	$3.01\times 10^{4}$	3.06 $\times \;10^{4}$
F22	3.64 $\times \;10^{3}$	$3.45\times 10^{3}$	3.55 $\times \;10^{3}$	3.78 $\times \;10^{3}$
F23	4.43 $\times \;10^{3}$	$4.14\times 10^{3}$	4.28 $\times \;10^{3}$	4.80 $\times \;10^{3}$
F24	4.74 $\times \;10^{3}$	3.85 $\times \;10^{3}$	$3.71\times 10^{3}$	3.79 $\times \;10^{3}$
F25	1.80 $\times \;10^{4}$	$1.54\times 10^{4}$	1.77 $\times \;10^{4}$	2.17 $\times \;10^{4}$
F26	3.78 $\times \;10^{3}$	$3.76\times 10^{3}$	3.84 $\times \;10^{3}$	4.06 $\times \;10^{3}$
F27	5.77 $\times \;10^{3}$	4.46 $\times \;10^{3}$	$4.17\times 10^{3}$	4.77 $\times \;10^{3}$
F28	1.02 $\times \;10^{4}$	$6.81\times 10^{3}$	6.87 $\times \;10^{3}$	7.46 $\times \;10^{3}$
F29	8.33 $\times \;10^{5}$	$4.23\times 10^{5}$	8.30 $\times \;10^{5}$	2.52 $\times \;10^{6}$
Average ranking	3.21	2.10	**1.97**	2.72
Total ranking	4	2	1	3

The bold part indicates the optimal result.

**Table 6:** Results of FBCA with variations in the Gaussian item N.

F	BCA	FBCA (N($0,0.05^{2}$))	FBCA (N($0,0.1^{2}$))	FBCA (N($0,0.2^{2}$))
F1	9.34 $\times \;10^{9}$	4.95 $\times \;10^{8}$	3.25 $\times \;10^{8}$	$2.63\times \;10^{8}$
F2	1.22 $\times \;10^{6}$	6.82 $\times \;10^{5}$	$6.36\times \;10^{5}$	6.87 $\times \;10^{5}$
F3	1.72 $\times \;10^{3}$	1.06 $\times \;10^{3}$	1.03 $\times \;10^{3}$	$9.62\times \;10^{2}$
F4	1.62 $\times \;10^{3}$	1.25 $\times \;10^{3}$	1.25 $\times \;10^{3}$	$1.24\times \;10^{3}$
F5	$6.31\times \;10^{2}$	6.39 $\times \;10^{2}$	6.40 $\times \;10^{2}$	6.42 $\times \;10^{2}$
F6	$2.54\times \;10^{3}$	2.77 $\times \;10^{3}$	2.63 $\times \;10^{3}$	2.78 $\times \;10^{3}$
F7	1.84 $\times \;10^{3}$	1.61 $\times \;10^{3}$	$1.56\times \;10^{3}$	1.57 $\times \;10^{3}$
F8	$4.00\times \;10^{4}$	6.84 $\times \;10^{4}$	7.08 $\times \;10^{4}$	6.45 $\times \;10^{4}$
F9	3.37 $\times \;10^{4}$	$2.80\times \;10^{4}$	2.93 $\times \;10^{4}$	2.84 $\times \;10^{4}$
F10	1.97 $\times \;10^{5}$	$5.31\times \;10^{4}$	5.89 $\times \;10^{4}$	5.35 $\times \;10^{4}$
F11	5.26 $\times \;10^{8}$	$9.98\times \;10^{7}$	1.18 $\times \;10^{8}$	1.27 $\times \;10^{8}$
F12	$1.60\times \;10^{4}$	4.10 $\times \;10^{4}$	4.69 $\times \;10^{4}$	6.32 $\times \;10^{4}$
F13	5.30 $\times \;10^{6}$	3.71 $\times \;10^{6}$	2.85 $\times \;10^{6}$	$2.26\times \;10^{6}$
F14	$5.63\times \;10^{3}$	2.07 $\times \;10^{4}$	1.65 $\times \;10^{4}$	1.30 $\times \;10^{4}$
F15	1.16 $\times \;10^{4}$	7.53 $\times \;10^{3}$	$5.70\times \;10^{3}$	6.57 $\times \;10^{3}$
F16	8.38 $\times \;10^{3}$	5.42 $\times \;10^{3}$	$5.41\times \;10^{3}$	5.85 $\times \;10^{3}$
F17	2.33 $\times \;10^{7}$	8.70 $\times \;10^{6}$	$7.47\times \;10^{6}$	7.92 $\times \;10^{6}$
F18	$6.30\times \;10^{3}$	8.28 $\times \;10^{3}$	1.23 $\times \;10^{4}$	1.24 $\times \;10^{4}$
F19	8.27 $\times \;10^{3}$	6.81 $\times \;10^{3}$	$6.79\times \;10^{3}$	7.15 $\times \;10^{3}$
F20	3.41 $\times \;10^{3}$	3.11 $\times \;10^{3}$	$3.10\times \;10^{3}$	3.14 $\times \;10^{3}$
F21	3.61 $\times \;10^{4}$	3.16 $\times \;10^{4}$	$3.02\times \;10^{4}$	3.11 $\times \;10^{4}$
F22	3.60 $\times \;10^{3}$	$3.53\times \;10^{3}$	3.60 $\times \;10^{3}$	3.54 $\times \;10^{3}$
F23	4.45 $\times \;10^{3}$	4.38 $\times \;10^{3}$	4.34 $\times \;10^{3}$	$4.31\times \;10^{3}$
F24	4.64 $\times \;10^{3}$	3.86 $\times \;10^{3}$	3.72 $\times \;10^{3}$	$3.67\times \;10^{3}$
F25	1.74 $\times \;10^{4}$	1.72 $\times \;10^{4}$	1.74 $\times \;10^{4}$	$1.63\times \;10^{4}$
F26	$3.77\times \;10^{3}$	3.83 $\times \;10^{3}$	3.79 $\times \;10^{3}$	3.80 $\times \;10^{3}$
F27	5.98 $\times \;10^{3}$	4.35 $\times \;10^{3}$	$4.01\times \;10^{3}$	4.03 $\times \;10^{3}$
F28	9.16 $\times \;10^{3}$	$6.98\times \;10^{3}$	7.00 $\times \;10^{3}$	7.04 $\times \;10^{3}$
F29	8.00 $\times \;10^{5}$	$5.21\times \;10^{5}$	9.02 $\times \;10^{5}$	1.26 $\times \;10^{6}$
Average ranking	3.14	2.38	**2.14**	2.34
Total ranking	4	3	1	2

The bold part indicates the optimal result.

**Table 7:** Results of FBCA with various spiral mechanisms.

F	BCA	FBCA (${\mathrm{Spiral}}_{1}$)	FBCA (${\mathrm{Spiral}}_{2}$)	FBCA (${\mathrm{Spiral}}_{3}$)	FBCA (${\mathrm{Spiral}}_{4}$)
F1	9.41 $\times \;10^{9}$	3.34 $\times \;10^{8}$	$2.82\times 10^{8}$	2.53 $\times \;10^{9}$	$7.29\times 10^{8}$
F2	1.72 $\times \;10^{6}$	6.79 $\times \;10^{5}$	6.91 $\times \;10^{5}$	6.62 $\times \;10^{5}$	$6.61\times 10^{5}$
F3	1.74 $\times \;10^{3}$	$9.98\times 10^{2}$	1.02 $\times \;10^{3}$	1.43 $\times \;10^{3}$	1.11 $\times \;10^{3}$
F4	1.59 $\times \;10^{3}$	$1.23\times 10^{3}$	1.27 $\times \;10^{3}$	1.39 $\times \;10^{3}$	1.28 $\times \;10^{3}$
F5	$6.32\times 10^{2}$	6.39 $\times \;10^{2}$	6.41 $\times \;10^{2}$	6.38 $\times \;10^{2}$	6.41 $\times \;10^{2}$
F6	$2.50\times 10^{3}$	2.63 $\times \;10^{3}$	2.68 $\times \;10^{3}$	2.61 $\times \;10^{3}$	3.15 $\times \;10^{3}$
F7	1.80 $\times \;10^{3}$	1.59 $\times \;10^{3}$	$1.55\times 10^{3}$	1.69 $\times \;10^{3}$	1.57 $\times \;10^{3}$
F8	$3.86\times 10^{4}$	7.30 $\times \;10^{4}$	7.51 $\times \;10^{4}$	6.76 $\times \;10^{4}$	7.88 $\times \;10^{4}$
F9	3.37 $\times \;10^{4}$	3.00 $\times \;10^{4}$	2.92 $\times \;10^{4}$	3.08 $\times \;10^{4}$	$2.79\times 10^{4}$
F10	2.14 $\times \;10^{5}$	$5.01\times 10^{4}$	5.09 $\times \;10^{4}$	6.90 $\times \;10^{4}$	5.34 $\times \;10^{4}$
F11	4.22 $\times \;10^{8}$	$1.09\times 10^{8}$	1.11 $\times \;10^{8}$	2.11 $\times \;10^{8}$	2.00 $\times \;10^{8}$
F12	$1.47\times 10^{4}$	7.00 $\times \;10^{4}$	1.31 $\times \;10^{5}$	1.22 $\times \;10^{5}$	7.91 $\times \;10^{6}$
F13	4.32 $\times \;10^{6}$	2.93 $\times \;10^{6}$	3.90 $\times \;10^{6}$	$2.75\times 10^{6}$	3.74 $\times \;10^{6}$
F14	$8.42\times 10^{3}$	2.09 $\times \;10^{4}$	1.13 $\times \;10^{4}$	2.12 $\times \;10^{4}$	1.75 $\times \;10^{4}$
F15	1.13 $\times \;10^{4}$	6.01 $\times \;10^{3}$	$5.80\times 10^{3}$	7.56 $\times \;10^{3}$	6.87 $\times \;10^{3}$
F16	8.33 $\times \;10^{3}$	$4.94\times 10^{3}$	5.48 $\times \;10^{3}$	5.60 $\times \;10^{3}$	5.63 $\times \;10^{3}$
F17	2.38 $\times \;10^{7}$	6.50 $\times \;10^{6}$	$5.60\times 10^{6}$	7.75 $\times \;10^{6}$	6.48 $\times \;10^{6}$
F18	$7.42\times 10^{3}$	1.15 $\times \;10^{4}$	1.92 $\times \;10^{4}$	1.05 $\times \;10^{4}$	2.49 $\times \;10^{4}$
F19	8.27 $\times \;10^{3}$	6.80 $\times \;10^{3}$	6.43 $\times \;10^{3}$	6.99 $\times \;10^{3}$	$6.32\times 10^{3}$
F20	3.39 $\times \;10^{3}$	$3.11\times 10^{3}$	3.13 $\times \;10^{3}$	3.20 $\times \;10^{3}$	3.12 $\times \;10^{3}$
F21	3.62 $\times \;10^{4}$	2.99 $\times \;10^{4}$	3.14 $\times \;10^{4}$	3.32 $\times \;10^{4}$	$2.72\times 10^{4}$
F22	3.64 $\times \;10^{3}$	3.58 $\times \;10^{3}$	3.62 $\times \;10^{3}$	$3.51\times 10^{3}$	3.67 $\times \;10^{3}$
F23	4.40 $\times \;10^{3}$	4.32 $\times \;10^{3}$	4.49 $\times \;10^{3}$	$4.20\times 10^{3}$	4.63 $\times \;10^{3}$
F24	4.68 $\times \;10^{3}$	3.75 $\times \;10^{3}$	$3.70\times 10^{3}$	4.10 $\times \;10^{3}$	3.79 $\times \;10^{3}$
F25	1.67 $\times \;10^{4}$	1.69 $\times \;10^{4}$	1.77 $\times \;10^{4}$	$1.54\times 10^{4}$	2.08 $\times \;10^{4}$
F26	3.82 $\times \;10^{3}$	3.82 $\times \;10^{3}$	3.86 $\times \;10^{3}$	$3.79\times 10^{3}$	4.04 $\times \;10^{3}$
F27	5.86 $\times \;10^{3}$	$4.23\times 10^{3}$	4.31 $\times \;10^{3}$	4.68 $\times \;10^{3}$	4.68 $\times \;10^{3}$
F28	9.04 $\times \;10^{3}$	7.23 $\times \;10^{3}$	$7.04\times 10^{3}$	7.19 $\times \;10^{3}$	7.57 $\times \;10^{3}$
F29	$6.45\times 10^{5}$	9.12 $\times \;10^{5}$	7.95 $\times \;10^{5}$	8.41 $\times \;10^{5}$	2.11 $\times \;10^{6}$
Average ranking	3.76	**2.28**	2.59	2.97	3.41
Total ranking	5	1	2	3	4

The bold part indicates the optimal result.

**Table 8:** Results of FBCA with various velocity update mechanisms.

F	BCA	FBCA (${\mathrm{velocity}}_{1}$)	FBCA (${\mathrm{velocity}}_{2}$)
F1	9.11 $\times \;10^{9}$	$2.11\times 10^{8}$	4.05 $\times \;10^{8}$
F2	9.05 $\times \;10^{5}$	6.76 $\times \;10^{5}$	$6.42\times 10^{5}$
F3	1.92 $\times \;10^{3}$	$9.74\times 10^{2}$	1.01 $\times \;10^{3}$
F4	1.62 $\times \;10^{3}$	$1.22\times 10^{3}$	1.23 $\times \;10^{3}$
F5	$6.32\times 10^{2}$	6.41 $\times \;10^{2}$	6.43 $\times \;10^{2}$
F6	$2.58\times 10^{3}$	2.78 $\times \;10^{3}$	2.71 $\times \;10^{3}$
F7	1.89 $\times \;10^{3}$	$1.54\times 10^{3}$	1.60 $\times \;10^{3}$
F8	$4.24\times 10^{4}$	7.03 $\times \;10^{4}$	7.28 $\times \;10^{4}$
F9	3.34 $\times \;10^{4}$	2.79 $\times \;10^{4}$	$2.39\times 10^{4}$
F10	1.95 $\times \;10^{5}$	$5.39\times 10^{4}$	5.86 $\times \;10^{4}$
F11	6.07 $\times \;10^{8}$	$9.92\times 10^{7}$	1.09 $\times \;10^{8}$
F12	2.82 $\times \;10^{5}$	$4.22\times 10^{4}$	5.38 $\times \;10^{4}$
F13	6.83 $\times \;10^{6}$	3.12 $\times \;10^{6}$	$2.36\times 10^{6}$
F14	$7.07\times 10^{3}$	1.50 $\times \;10^{4}$	1.17 $\times \;10^{4}$
F15	1.15 $\times \;10^{4}$	$6.53\times 10^{3}$	7.03 $\times \;10^{3}$
F16	8.24 $\times \;10^{3}$	5.67 $\times \;10^{3}$	$5.04\times 10^{3}$
F17	1.90 $\times \;10^{7}$	6.91 $\times \;10^{6}$	$5.39\times 10^{6}$
F18	$6.01\times 10^{3}$	1.35 $\times \;10^{4}$	1.17 $\times \;10^{4}$
F19	8.30 $\times \;10^{3}$	$6.40\times 10^{3}$	7.07 $\times \;10^{3}$
F20	3.42 $\times \;10^{3}$	$3.09\times 10^{3}$	3.17 $\times \;10^{3}$
F21	3.57 $\times \;10^{4}$	3.03 $\times \;10^{4}$	$2.61\times 10^{4}$
F22	3.65 $\times \;10^{3}$	$3.57\times 10^{3}$	3.59 $\times \;10^{3}$
F23	4.35 $\times \;10^{3}$	$4.29\times 10^{3}$	4.34 $\times \;10^{3}$
F24	4.76 $\times \;10^{3}$	$3.73\times 10^{3}$	3.74 $\times \;10^{3}$
F25	$1.61\times 10^{4}$	1.77 $\times \;10^{4}$	1.75 $\times \;10^{4}$
F26	3.80 $\times \;10^{3}$	3.81 $\times \;10^{3}$	$3.79\times 10^{3}$
F27	5.85 $\times \;10^{3}$	$4.13\times 10^{3}$	4.14 $\times \;10^{3}$
F28	9.33 $\times \;10^{3}$	$6.92\times 10^{3}$	7.28 $\times \;10^{3}$
F29	$9.00\times 10^{5}$	9.07 $\times \;10^{5}$	9.53 $\times \;10^{5}$
Average ranking	2.48	**1.66**	1.86
Total ranking	3	1	2

The bold part indicates the optimal result.

**Table 9:** Results of FBCA versus L-SHADE and other SOTA optimizers.

F	FBCA	BCA	SaDE	L-SHADE	L-SHADE_EpSin
F1	3.29 $\times \;10^{8}$	8.02 $\times \;10^{9}$	9.24 $\times \;10^{9}$	$2.82\times 10^{8}$	5.07 $\times \;10^{10}$
F2	6.90 $\times \;10^{5}$	1.21 $\times \;10^{6}$	$3.54\times 10^{5}$	5.85 $\times \;10^{5}$	3.85 $\times \;10^{5}$
F3	9.97 $\times \;10^{2}$	1.88 $\times \;10^{3}$	2.11 $\times \;10^{3}$	$9.61\times 10^{2}$	6.22 $\times \;10^{3}$
F4	$1.21\times 10^{3}$	1.55 $\times \;10^{3}$	1.37 $\times \;10^{3}$	1.51 $\times \;10^{3}$	1.23 $\times \;10^{3}$
F5	6.40 $\times \;10^{2}$	6.31 $\times \;10^{2}$	6.49 $\times \;10^{2}$	$6.14\times 10^{2}$	6.46 $\times \;10^{2}$
F6	2.66 $\times \;10^{3}$	2.47 $\times \;10^{3}$	2.53 $\times \;10^{3}$	$2.05\times 10^{3}$	2.56 $\times \;10^{3}$
F7	$1.56\times 10^{3}$	1.85 $\times \;10^{3}$	1.69 $\times \;10^{3}$	1.81 $\times \;10^{3}$	1.57 $\times \;10^{3}$
F8	7.07 $\times \;10^{4}$	4.35 $\times \;10^{4}$	5.61 $\times \;10^{4}$	$1.66\times 10^{4}$	2.69 $\times \;10^{4}$
F9	2.73 $\times \;10^{4}$	3.34 $\times \;10^{4}$	3.26 $\times \;10^{4}$	3.20 $\times \;10^{4}$	$2.22\times 10^{4}$
F10	$6.00\times 10^{4}$	2.15 $\times \;10^{5}$	6.63 $\times \;10^{4}$	1.20 $\times \;10^{5}$	7.87 $\times \;10^{4}$
F11	1.09 $\times \;10^{8}$	3.92 $\times \;10^{8}$	5.11 $\times \;10^{8}$	$4.75\times 10^{7}$	4.72 $\times \;10^{9}$
F12	4.09 $\times \;10^{4}$	$1.37\times 10^{4}$	9.59 $\times \;10^{4}$	1.46 $\times \;10^{4}$	8.09 $\times \;10^{7}$
F13	3.04 $\times \;10^{6}$	5.61 $\times \;10^{6}$	$5.40\times 10^{5}$	4.53 $\times \;10^{6}$	3.30 $\times \;10^{6}$
F14	1.24 $\times \;10^{4}$	7.58 $\times \;10^{3}$	2.14 $\times \;10^{5}$	$5.07\times 10^{3}$	3.60 $\times \;10^{5}$
F15	6.89 $\times \;10^{3}$	1.16 $\times \;10^{4}$	6.91 $\times \;10^{3}$	9.73 $\times \;10^{3}$	$6.31\times 10^{3}$
F16	5.21 $\times \;10^{3}$	8.13 $\times \;10^{3}$	6.70 $\times \;10^{3}$	7.18 $\times \;10^{3}$	$5.09\times 10^{3}$
F17	6.75 $\times \;10^{6}$	1.97 $\times \;10^{7}$	$1.52\times 10^{6}$	7.74 $\times \;10^{6}$	4.08 $\times \;10^{6}$
F18	1.25 $\times \;10^{4}$	1.10 $\times \;10^{4}$	4.10 $\times \;10^{5}$	$4.66\times 10^{3}$	7.06 $\times \;10^{6}$
F19	7.01 $\times \;10^{3}$	8.27 $\times \;10^{3}$	7.63 $\times \;10^{3}$	7.49 $\times \;10^{3}$	$5.61\times 10^{3}$
F20	$3.07\times 10^{3}$	3.47 $\times \;10^{3}$	3.30 $\times \;10^{3}$	3.36 $\times \;10^{3}$	3.15 $\times \;10^{3}$
F21	3.09 $\times \;10^{4}$	3.61 $\times \;10^{4}$	3.44 $\times \;10^{4}$	3.42 $\times \;10^{4}$	$2.44\times 10^{4}$
F22	3.61 $\times \;10^{3}$	$3.60\times 10^{3}$	3.69 $\times \;10^{3}$	3.83 $\times \;10^{3}$	3.99 $\times \;10^{3}$
F23	$4.30\times 10^{3}$	4.39 $\times \;10^{3}$	4.48 $\times \;10^{3}$	4.40 $\times \;10^{3}$	5.18 $\times \;10^{3}$
F24	3.68 $\times \;10^{3}$	4.54 $\times \;10^{3}$	4.82 $\times \;10^{3}$	$3.66\times 10^{3}$	7.38 $\times \;10^{3}$
F25	$1.70\times 10^{4}$	1.73 $\times \;10^{4}$	1.73 $\times \;10^{4}$	1.72 $\times \;10^{4}$	2.41 $\times \;10^{4}$
F26	3.83 $\times \;10^{3}$	3.81 $\times \;10^{3}$	3.87 $\times \;10^{3}$	$3.60\times 10^{3}$	4.82 $\times \;10^{3}$
F27	4.27 $\times \;10^{3}$	5.95 $\times \;10^{3}$	5.95 $\times \;10^{3}$	$4.03\times 10^{3}$	1.10 $\times \;10^{4}$
F28	$6.93\times 10^{3}$	9.42 $\times \;10^{3}$	8.30 $\times \;10^{3}$	9.42 $\times \;10^{3}$	9.72 $\times \;10^{3}$
F29	7.57 $\times \;10^{5}$	7.75 $\times \;10^{5}$	5.53 $\times \;10^{6}$	$1.14\times 10^{5}$	1.72 $\times \;10^{8}$
Average ranking	**2.24**	3.51	3.34	2.41	3.48
Total ranking	1	5	3	2	4

The bold part indicates the optimal result.

**Table 10:** Classification problems.

Datasets	Feature Numbers	Training Samples	Test Samples	Number of Classes	MLP Structure	Dimension
XOR	3	8	8	2	3-7-1	36
Iris	4	150	150	3	4-9-3	75
Heart	22	80	80	2	22-45-1	1081

**Table 11:** Function-approximation problems.

Datasets	Training Samples	Test Samples	MLP Structure	Dimension
Sigmoid: $y=\frac{1}{1+e^{\left(-x\right)}}$	61: *x* in [−3:0.1:3]	121: *x* in [−3:0.05:3]	1-15-1	46
Cosine: $y={\left(\cos(x\pi /2)\right)}^{7}$	31: *x* in [1.25:0.05:2.75]	38: *x* in [1.25:0.04:2.75]	1-15-1	46
Sine: y=sin(2x)	126: *x* in [−2π:0.1:2π]	252: *x* in [−2π:0.05:2π]	1-15-1	46

**Table 12:** Comparison optimization results of MLP_XOR problem.

	FBCA	BCA	GA	SMA	HHO	OOA	COA	GLS	HOA	RSA
Mean	$9.589\times 10^{-6}$	2.93 $\times \;10^{-3}$	6.863 $\times \;10^{-2}$	2.003 $\times \;10^{-1}$	8.166 $\times \;10^{-5}$	1.779 $\times \;10^{-1}$	1.663 $\times \;10^{-1}$	3.567 $\times \;10^{-2}$	1.149 $\times \;10^{-1}$	1.555 $\times \;10^{-1}$
Std	1.573 $\times \;10^{-5}$	8.72 $\times \;10^{-3}$	6.513 $\times \;10^{-2}$	4.472 $\times \;10^{-2}$	1.244 $\times \;10^{-4}$	4.815 $\times \;10^{-2}$	6.867 $\times \;10^{-2}$	4.549 $\times \;10^{-2}$	5.087 $\times \;10^{-2}$	3.516 $\times \;10^{-2}$
Accuracy	**100%**	100%	62.5%	12.5%	100%	37.5%	37.5%	100%	25%	12.5%

The bold part indicates the optimal result.

**Table 13:** Comparison optimization results of MLP_Iris problem.

	FBCA	BCA	GA	SMA	HHO	OOA	COA	GLS	HOA	RSA
Mean	$2.672\times 10^{-2}$	2.89 $\times \;10^{-2}$	2.946 $\times \;10^{-1}$	6.596 $\times \;10^{-2}$	6.572 $\times \;10^{-2}$	4.342 $\times \;10^{-1}$	4.221 $\times \;10^{-1}$	1.224 $\times \;10^{-1}$	2.507 $\times \;10^{-1}$	3.044 $\times \;10^{-1}$
Std	6.321 $\times \;10^{-3}$	1.316 $\times \;10^{-2}$	1.878 $\times \;10^{-1}$	4.507 $\times \;10^{-2}$	1.044 $\times \;10^{-1}$	1.009 $\times \;10^{-1}$	7.079 $\times \;10^{-2}$	4.89 $\times \;10^{-2}$	5.174 $\times \;10^{-2}$	4.194 $\times \;10^{-2}$
Accuracy	**88.67%**	86%	25.33%	43.33%	74%	6.67%	14%	54%	5.33%	7.33%

The bold part indicates the optimal result.

**Table 14:** Comparison optimization results of MLP_Heart problem.

	FBCA	BCA	GA	SMA	HHO	OOA	COA	GLS	HOA	RSA
Mean	$9.779\times 10^{-2}$	1.101 $\times \;10^{-1}$	2.826 $\times \;10^{-1}$	1.681 $\times \;10^{-1}$	1.257 $\times \;10^{-1}$	1.76 $\times \;10^{-1}$	1.718 $\times \;10^{-1}$	1.474 $\times \;10^{-1}$	1.242 $\times \;10^{-1}$	1.626 $\times \;10^{-1}$
Std	1.179 $\times \;10^{-2}$	3.974 $\times \;10^{-2}$	3.916 $\times \;10^{-2}$	6.858 $\times \;10^{-3}$	8.339 $\times \;10^{-3}$	6.495 $\times \;10^{-3}$	7.723 $\times \;10^{-3}$	2.258 $\times \;10^{-2}$	1.114 $\times \;10^{-2}$	1.167 $\times \;10^{-2}$
Accuracy	**83.75%**	82.5%	52.5%	73.75%	73.75%	32.5%	36.25%	78.75%	67.5%	48.75%

The bold part indicates the optimal result.

**Table 15:** Comparison optimization results of MLP_Sigmoid problem.

	FBCA	BCA	GA	SMA	HHO	OOA	COA	GLS	HOA	RSA
Mean	$2.466\times 10^{-1}$	2.482 $\times \;10^{-1}$	2.486 $\times \;10^{-1}$	2.468 $\times \;10^{-1}$	2.467 $\times \;10^{-1}$	2.496 $\times \;10^{-1}$	2.486 $\times \;10^{-1}$	2.469 $\times \;10^{-1}$	2.477 $\times \;10^{-1}$	2.471 $\times \;10^{-1}$
Std	1.711 $\times \;10^{-4}$	1.759 $\times \;10^{-3}$	1.909 $\times \;10^{-3}$	2.321 $\times \;10^{-4}$	1.503 $\times \;10^{-4}$	1.807 $\times \;10^{-3}$	1.835 $\times \;10^{-3}$	4.225 $\times \;10^{-4}$	7.978 $\times \;10^{-4}$	3.776 $\times \;10^{-4}$
Error	**17.5564**	18.3290	19.4690	17.8225	17.5827	20.5183	17.7487	18.1118	17.8106	18.1837

The bold part indicates the optimal result.

**Table 16:** Comparison optimization results of MLP_Cosine problem.

	FBCA	BCA	GA	SMA	HHO	OOA	COA	GLS	HOA	RSA
Mean	$1.772\times 10^{-1}$	1.826 $\times \;10^{-1}$	1.98 $\times \;10^{-1}$	1.816 $\times \;10^{-1}$	1.774 $\times \;10^{-1}$	2.756 $\times \;10^{-1}$	2.244 $\times \;10^{-1}$	1.79 $\times \;10^{-1}$	1.85 $\times \;10^{-1}$	2.001 $\times \;10^{-1}$
Std	4.262 $\times \;10^{-6}$	4.262 $\times \;10^{-6}$	4.262 $\times \;10^{-6}$	4.262 $\times \;10^{-6}$	4.262 $\times \;10^{-6}$	4.262 $\times \;10^{-6}$	4.262 $\times \;10^{-6}$	4.262 $\times \;10^{-6}$	4.262 $\times \;10^{-6}$	4.262 $\times \;10^{-6}$
Error	**4.6792**	5.2449	8.9720	4.7839	4.7741	6.0326	7.4608	5.0299	5.3183	6.0737

The bold part indicates the optimal result.

**Table 17:** Comparison optimization results of MLP_Sine problem.

	FBCA	BCA	GA	SMA	HHO	OOA	COA	GLS	HOA	RSA
Mean	$4.453\times 10^{-1}$	4.514 $\times \;10^{-1}$	4.655 $\times \;10^{-1}$	4.523 $\times \;10^{-1}$	4.462 $\times \;10^{-1}$	4.649 $\times \;10^{-1}$	4.523 $\times \;10^{-1}$	4.495 $\times \;10^{-1}$	4.611 $\times \;10^{-1}$	4.677 $\times \;10^{-1}$
Std	8.941 $\times \;10^{-3}$	4.996 $\times \;10^{-3}$	9.217 $\times \;10^{-3}$	9.393 $\times \;10^{-3}$	2.225 $\times \;10^{-3}$	4.332 $\times \;10^{-3}$	1.138 $\times \;10^{-2}$	9.507 $\times \;10^{-3}$	3.623 $\times \;10^{-3}$	7.564 $\times \;10^{-3}$
Error	**146.5873**	147.1405	157.9612	148.8101	147.4074	14.9740	146.9557	148.6581	151.0190	153.4001

The bold part indicates the optimal result.

**Table 18:** Comparison results of FBCA and the gradient-based optimizers.

Datasets	Item	FBCA	BCA	SGD	Adam	RMSprop	Adagrad
MLP_Heart	Mean	9.779 $\times \;10^{-2}$	1.101 $\times \;10^{-1}$	9.212 $\times \;10^{-2}$	5.580 $\times \;10^{-2}$	4.331 $\times \;10^{-2}$	5.821 $\times \;10^{-2}$
	Accuracy	**83.75%**	82.5%	31.25%	71.25%	72.5%	76.25%
MLP_Sine	Mean	4.453 $\times \;10^{-1}$	4.514 $\times \;10^{-1}$	4.983 $\times \;10^{-1}$	4.453 $\times \;10^{-1}$	4.453 $\times \;10^{-1}$	4.896 $\times \;10^{-1}$
	Error	**146.5873**	147.1405	159.7947	148.4509	146.7834	157.5333

The bold part indicates the optimal result.

## Data Availability

The datasets analyzed during the current research can be obtained from the University of California, Irvine (UCI) Machine Learning Repository (https://archive.ics.uci.edu/, accessed on 1 January 2025).

## Appendix A

IEEE CEC 2017 test suite detailed experimental data.

**Table A1:** Comparison of the FA-BCA with other algorithms on IEEE CEC 2017 benchmark functions with D = 30.

F	Item	FBCA	BCA	SCA	GA	RSA	PSO	DBO	BKA	HHO	HOA	COA	CPO	PO	SMA
F1	Std	6.44 $\times \;10^{3}$	5.91 $\times \;10^{3}$	3.47 $\times \;10^{9}$	2.06 $\times \;10^{10}$	7.83 $\times \;10^{9}$	2.37 $\times \;10^{9}$	2.32 $\times \;10^{8}$	6.8 $\times \;10^{9}$	2.39 $\times \;10^{8}$	8.68 $\times \;10^{9}$	5.96 $\times \;10^{9}$	8.43 $\times \;10^{5}$	1.31 $\times \;10^{9}$	6.95 $\times \;10^{4}$
	Mean	1.13 $\times \;10^{4}$	6.64 $\times \;10^{3}$	2.07 $\times \;10^{10}$	5.30 $\times \;10^{10}$	4.80 $\times \;10^{10}$	4.22 $\times \;10^{9}$	2.75 $\times \;10^{8}$	9.35 $\times \;10^{9}$	4.72 $\times \;10^{8}$	3.71 $\times \;10^{10}$	5.70 $\times \;10^{10}$	7.47 $\times \;10^{5}$	3.23 $\times \;10^{9}$	1.33 $\times \;10^{5}$
F2	Std	2.69 $\times \;10^{4}$	3.64 $\times \;10^{4}$	1.70 $\times \;10^{4}$	6.02 $\times \;10^{4}$	5.99 $\times \;10^{3}$	7.69 $\times \;10^{4}$	2.64 $\times \;10^{4}$	1.88 $\times \;10^{4}$	6.81 $\times \;10^{3}$	6.74 $\times \;10^{3}$	5.87 $\times \;10^{3}$	1.10 $\times \;10^{4}$	8.57 $\times \;10^{3}$	1.35 $\times \;10^{4}$
	Mean	7.88 $\times \;10^{4}$	1.40 $\times \;10^{5}$	8.19 $\times \;10^{4}$	2.59 $\times \;10^{5}$	8.10 $\times \;10^{4}$	1.76 $\times \;10^{5}$	8.97 $\times \;10^{4}$	3.74 $\times \;10^{4}$	5.76 $\times \;10^{4}$	7.33 $\times \;10^{4}$	8.49 $\times \;10^{4}$	6.18 $\times \;10^{4}$	6.81 $\times \;10^{4}$	3.42 $\times \;10^{4}$
F3	Std	3.89 $\times \;10^{1}$	2.64 $\times \;10^{1}$	8.15 $\times \;10^{2}$	4.64 $\times \;10^{3}$	3.61 $\times \;10^{3}$	4.42 $\times \;10^{2}$	1.18 $\times \;10^{2}$	7.73 $\times \;10^{2}$	1.02 $\times \;10^{2}$	1.74 $\times \;10^{3}$	2.24 $\times \;10^{3}$	1.67 $\times \;10^{1}$	1.08 $\times \;10^{2}$	2.38 $\times \;10^{1}$
	Mean	4.93 $\times \;10^{2}$	5.06 $\times \;10^{2}$	2.91 $\times \;10^{3}$	9.24 $\times \;10^{3}$	1.09 $\times \;10^{4}$	1.24 $\times \;10^{3}$	6.44 $\times \;10^{2}$	1.27 $\times \;10^{3}$	7.23 $\times \;10^{2}$	7.95 $\times \;10^{3}$	1.57 $\times \;10^{4}$	5.24 $\times \;10^{2}$	7.51 $\times \;10^{2}$	5.12 $\times \;10^{2}$
F4	Std	3.23 $\times \;10^{1}$	7.64 $\times \;10^{1}$	2.73 $\times \;10^{1}$	7.15 $\times \;10^{1}$	2.85 $\times \;10^{1}$	4.11 $\times \;10^{1}$	5.37 $\times \;10^{1}$	4.88 $\times \;10^{1}$	3.78 $\times \;10^{1}$	3.15 $\times \;10^{1}$	4.00 $\times \;10^{1}$	1.58 $\times \;10^{1}$	3.79 $\times \;10^{1}$	3.94 $\times \;10^{1}$
	Mean	6.31 $\times \;10^{2}$	6.56 $\times \;10^{2}$	8.29 $\times \;10^{2}$	9.94 $\times \;10^{2}$	9.22 $\times \;10^{2}$	8.07 $\times \;10^{2}$	7.73 $\times \;10^{2}$	7.52 $\times \;10^{2}$	7.75 $\times \;10^{2}$	7.99 $\times \;10^{2}$	9.10 $\times \;10^{2}$	6.91 $\times \;10^{2}$	7.88 $\times \;10^{2}$	6.43 $\times \;10^{2}$
F5	Std	9.77 $\times \;10^{0}$	8.97 $\times \;10^{0}$	5.03 $\times \;10^{0}$	1.40 $\times \;10^{1}$	4.68 $\times \;10^{0}$	1.55 $\times \;10^{1}$	1.23 $\times \;10^{1}$	7.04 $\times \;10^{0}$	6.25 $\times \;10^{0}$	7.89 $\times \;10^{0}$	6.08 $\times \;10^{0}$	7.25 $\times \;10^{0}$	7.62 $\times \;10^{0}$	9.20 $\times \;10^{0}$
	Mean	6.18 $\times \;10^{2}$	6.01 $\times \;10^{2}$	6.65 $\times \;10^{2}$	7.20 $\times \;10^{2}$	6.92 $\times \;10^{2}$	6.58 $\times \;10^{2}$	6.49 $\times \;10^{2}$	6.60 $\times \;10^{2}$	6.67 $\times \;10^{2}$	6.66 $\times \;10^{2}$	6.88 $\times \;10^{2}$	6.02 $\times \;10^{2}$	6.69 $\times \;10^{2}$	6.18 $\times \;10^{2}$
F6	Std	1.71 $\times \;10^{2}$	7.99 $\times \;10^{1}$	6.91 $\times \;10^{1}$	3.17 $\times \;10^{2}$	3.99 $\times \;10^{1}$	6.58 $\times \;10^{1}$	7.36 $\times \;10^{1}$	8.16 $\times \;10^{1}$	5.76 $\times \;10^{1}$	5.32 $\times \;10^{1}$	5.29 $\times \;10^{1}$	2.02 $\times \;10^{1}$	8.33 $\times \;10^{1}$	4.88 $\times \;10^{1}$
	Mean	1.02 $\times \;10^{3}$	9.49 $\times \;10^{2}$	1.24 $\times \;10^{3}$	2.07 $\times \;10^{3}$	1.38 $\times \;10^{3}$	1.14 $\times \;10^{3}$	1.01 $\times \;10^{3}$	1.23 $\times \;10^{3}$	1.30 $\times \;10^{3}$	1.26 $\times \;10^{3}$	1.42 $\times \;10^{3}$	9.39 $\times \;10^{2}$	1.23 $\times \;10^{3}$	8.92 $\times \;10^{2}$
F7	Std	5.31 $\times \;10^{1}$	6.03 $\times \;10^{1}$	2.15 $\times \;10^{1}$	8.58 $\times \;10^{1}$	1.85 $\times \;10^{1}$	4.10 $\times \;10^{1}$	5.77 $\times \;10^{1}$	4.94 $\times \;10^{1}$	2.63 $\times \;10^{1}$	2.63 $\times \;10^{1}$	2.36 $\times \;10^{1}$	1.44 $\times \;10^{1}$	3.80 $\times \;10^{1}$	3.86 $\times \;10^{1}$
	Mean	9.32 $\times \;10^{2}$	9.82 $\times \;10^{2}$	1.09 $\times \;10^{3}$	1.24 $\times \;10^{3}$	1.14 $\times \;10^{3}$	1.07 $\times \;10^{3}$	1.03 $\times \;10^{3}$	9.87 $\times \;10^{2}$	9.89 $\times \;10^{2}$	1.06 $\times \;10^{3}$	1.15 $\times \;10^{3}$	9.87 $\times \;10^{2}$	1.03 $\times \;10^{3}$	9.39 $\times \;10^{2}$
F8	Std	3.50 $\times \;10^{3}$	9.05 $\times \;10^{2}$	1.75 $\times \;10^{3}$	2.73 $\times \;10^{3}$	1.01 $\times \;10^{3}$	3.02 $\times \;10^{3}$	1.72 $\times \;10^{3}$	1.41 $\times \;10^{3}$	1.32 $\times \;10^{3}$	1.00 $\times \;10^{3}$	1.44 $\times \;10^{3}$	4.27 $\times \;10^{2}$	1.47 $\times \;10^{3}$	1.31 $\times \;10^{3}$
	Mean	4.59 $\times \;10^{3}$	1.53 $\times \;10^{3}$	9.14 $\times \;10^{3}$	1.05 $\times \;10^{4}$	1.14 $\times \;10^{4}$	7.75 $\times \;10^{3}$	6.19 $\times \;10^{3}$	5.73 $\times \;10^{3}$	8.69 $\times \;10^{3}$	6.74 $\times \;10^{3}$	1.11 $\times \;10^{4}$	1.35 $\times \;10^{3}$	7.64 $\times \;10^{3}$	4.57 $\times \;10^{3}$
F9	Std	1.31 $\times \;10^{3}$	1.13 $\times \;10^{3}$	3.30 $\times \;10^{2}$	6.14 $\times \;10^{2}$	4.49 $\times \;10^{2}$	6.63 $\times \;10^{2}$	1.03 $\times \;10^{3}$	1.28 $\times \;10^{3}$	8.04 $\times \;10^{2}$	6.67 $\times \;10^{2}$	4.53 $\times \;10^{2}$	2.33 $\times \;10^{2}$	7.52 $\times \;10^{2}$	6.18 $\times \;10^{2}$
	Mean	4.52 $\times \;10^{3}$	8.83 $\times \;10^{3}$	8.86 $\times \;10^{3}$	8.52 $\times \;10^{3}$	8.49 $\times \;10^{3}$	7.67 $\times \;10^{3}$	6.42 $\times \;10^{3}$	5.86 $\times \;10^{3}$	6.05 $\times \;10^{3}$	7.56 $\times \;10^{3}$	8.89 $\times \;10^{3}$	7.63 $\times \;10^{3}$	7.10 $\times \;10^{3}$	4.65 $\times \;10^{3}$
F10	Std	4.05 $\times \;10^{1}$	1.04 $\times \;10^{2}$	1.42 $\times \;10^{3}$	1.29 $\times \;10^{4}$	2.53 $\times \;10^{3}$	3.08 $\times \;10^{3}$	1.28 $\times \;10^{3}$	1.21 $\times \;10^{3}$	2.83 $\times \;10^{2}$	1.47 $\times \;10^{3}$	2.21 $\times \;10^{3}$	2.78 $\times \;10^{1}$	6.46 $\times \;10^{2}$	5.58 $\times \;10^{1}$
	Mean	1.21 $\times \;10^{3}$	1.26 $\times \;10^{3}$	4.11 $\times \;10^{3}$	2.44 $\times \;10^{4}$	9.06 $\times \;10^{3}$	4.41 $\times \;10^{3}$	1.97 $\times \;10^{3}$	1.87 $\times \;10^{3}$	1.63 $\times \;10^{3}$	5.99 $\times \;10^{3}$	9.17 $\times \;10^{3}$	1.28 $\times \;10^{3}$	2.57 $\times \;10^{3}$	1.29 $\times \;10^{3}$
F11	Std	8.96 $\times \;10^{5}$	1.25 $\times \;10^{6}$	7.78 $\times \;10^{8}$	4.71 $\times \;10^{9}$	3.25 $\times \;10^{9}$	6.09 $\times \;10^{8}$	1.16 $\times \;10^{8}$	1.42 $\times \;10^{9}$	9.04 $\times \;10^{7}$	1.94 $\times \;10^{9}$	3.95 $\times \;10^{9}$	8.74 $\times \;10^{5}$	2.76 $\times \;10^{8}$	3.98 $\times \;10^{6}$
	Mean	1.05 $\times \;10^{6}$	1.29 $\times \;10^{6}$	2.64 $\times \;10^{9}$	7.15 $\times \;10^{9}$	1.49 $\times \;10^{10}$	5.58 $\times \;10^{8}$	8.64 $\times \;10^{7}$	3.64 $\times \;10^{8}$	8.74 $\times \;10^{7}$	7.03 $\times \;10^{9}$	1.32 $\times \;10^{10}$	1.32 $\times \;10^{6}$	3.22 $\times \;10^{8}$	5.21 $\times \;10^{6}$
F12	Std	2.05 $\times \;10^{4}$	3.14 $\times \;10^{4}$	4.04 $\times \;10^{8}$	4.79 $\times \;10^{9}$	7.28 $\times \;10^{9}$	1.94 $\times \;10^{9}$	1.19 $\times \;10^{7}$	4.20 $\times \;10^{8}$	1.18 $\times \;10^{6}$	1.27 $\times \;10^{9}$	4.48 $\times \;10^{9}$	1.23 $\times \;10^{4}$	2.66 $\times \;10^{7}$	5.73 $\times \;10^{4}$
	Mean	1.57 $\times \;10^{4}$	1.92 $\times \;10^{4}$	1.11 $\times \;10^{9}$	4.69 $\times \;10^{9}$	1.26 $\times \;10^{10}$	8.49 $\times \;10^{8}$	4.70 $\times \;10^{6}$	1.41 $\times \;10^{8}$	1.20 $\times \;10^{6}$	2.27 $\times \;10^{9}$	1.03 $\times \;10^{10}$	2.27 $\times \;10^{4}$	1.14 $\times \;10^{7}$	7.87 $\times \;10^{4}$
F13	Std	2.04 $\times \;10^{5}$	3.89 $\times \;10^{5}$	1.03 $\times \;10^{6}$	1.10 $\times \;10^{7}$	6.65 $\times \;10^{6}$	3.04 $\times \;10^{6}$	7.61 $\times \;10^{5}$	1.33 $\times \;10^{5}$	1.82 $\times \;10^{6}$	8.72 $\times \;10^{5}$	2.85 $\times \;10^{6}$	8.48 $\times \;10^{2}$	6.30 $\times \;10^{5}$	1.64 $\times \;10^{5}$
	Mean	1.70 $\times \;10^{5}$	2.95 $\times \;10^{5}$	1.17 $\times \;10^{6}$	7.17 $\times \;10^{6}$	7.99 $\times \;10^{6}$	1.55 $\times \;10^{6}$	4.78 $\times \;10^{5}$	3.72 $\times \;10^{4}$	1.82 $\times \;10^{6}$	1.42 $\times \;10^{6}$	3.68 $\times \;10^{6}$	2.20 $\times \;10^{3}$	6.89 $\times \;10^{5}$	2.01 $\times \;10^{5}$
F14	Std	1.06 $\times \;10^{4}$	8.57 $\times \;10^{3}$	4.13 $\times \;10^{7}$	4.57 $\times \;10^{8}$	1.07 $\times \;10^{8}$	4.60 $\times \;10^{8}$	6.10 $\times \;10^{4}$	1.73 $\times \;10^{5}$	7.60 $\times \;10^{4}$	1.30 $\times \;10^{8}$	5.49 $\times \;10^{8}$	2.75 $\times \;10^{3}$	1.42 $\times \;10^{6}$	1.58 $\times \;10^{4}$
	Mean	1.28 $\times \;10^{4}$	8.97 $\times \;10^{3}$	5.91 $\times \;10^{7}$	3.77 $\times \;10^{8}$	5.48 $\times \;10^{8}$	1.23 $\times \;10^{8}$	8.34 $\times \;10^{4}$	9.08 $\times \;10^{4}$	1.33 $\times \;10^{5}$	1.35 $\times \;10^{8}$	6.68 $\times \;10^{8}$	4.65 $\times \;10^{3}$	9.81 $\times \;10^{5}$	2.93 $\times \;10^{4}$
F15	Std	3.68 $\times \;10^{2}$	6.71 $\times \;10^{2}$	2.19 $\times \;10^{2}$	7.08 $\times \;10^{2}$	6.62 $\times \;10^{2}$	5.32 $\times \;10^{2}$	4.92 $\times \;10^{2}$	4.53 $\times \;10^{2}$	4.25 $\times \;10^{2}$	6.54 $\times \;10^{2}$	1.06 $\times \;10^{3}$	2.17 $\times \;10^{2}$	2.96 $\times \;10^{2}$	2.66 $\times \;10^{2}$
	Mean	2.65 $\times \;10^{3}$	3.21 $\times \;10^{3}$	4.13 $\times \;10^{3}$	4.58 $\times \;10^{3}$	5.39 $\times \;10^{3}$	3.93 $\times \;10^{3}$	3.47 $\times \;10^{3}$	3.13 $\times \;10^{3}$	3.82 $\times \;10^{3}$	4.76 $\times \;10^{3}$	6.28 $\times \;10^{3}$	3.10 $\times \;10^{3}$	3.76 $\times \;10^{3}$	2.68 $\times \;10^{3}$
F16	Std	2.85 $\times \;10^{2}$	2.53 $\times \;10^{2}$	1.88 $\times \;10^{2}$	3.27 $\times \;10^{2}$	5.30 $\times \;10^{3}$	2.94 $\times \;10^{2}$	2.48 $\times \;10^{2}$	2.98 $\times \;10^{2}$	3.10 $\times \;10^{2}$	5.07 $\times \;10^{2}$	3.22 $\times \;10^{3}$	1.32 $\times \;10^{2}$	2.69 $\times \;10^{2}$	2.43 $\times \;10^{2}$
	Mean	2.28 $\times \;10^{3}$	2.04 $\times \;10^{3}$	2.77 $\times \;10^{3}$	3.15 $\times \;10^{3}$	7.29 $\times \;10^{3}$	2.62 $\times \;10^{3}$	2.66 $\times \;10^{3}$	2.44 $\times \;10^{3}$	2.63 $\times \;10^{3}$	3.06 $\times \;10^{3}$	5.50 $\times \;10^{3}$	2.04 $\times \;10^{3}$	2.68 $\times \;10^{3}$	2.40 $\times \;10^{3}$
F17	Std	1.63 $\times \;10^{6}$	3.87 $\times \;10^{6}$	5.88 $\times \;10^{6}$	6.92 $\times \;10^{7}$	2.53 $\times \;10^{7}$	3.54 $\times \;10^{7}$	6.54 $\times \;10^{6}$	1.94 $\times \;10^{5}$	4.35 $\times \;10^{6}$	1.49 $\times \;10^{7}$	4.72 $\times \;10^{7}$	7.34 $\times \;10^{4}$	9.27 $\times \;10^{6}$	3.52 $\times \;10^{6}$
	Mean	1.50 $\times \;10^{6}$	3.46 $\times \;10^{6}$	1.17 $\times \;10^{7}$	4.56 $\times \;10^{7}$	3.74 $\times \;10^{7}$	1.53 $\times \;10^{7}$	3.90 $\times \;10^{6}$	1.98 $\times \;10^{5}$	3.90 $\times \;10^{6}$	1.53 $\times \;10^{7}$	5.31 $\times \;10^{7}$	1.44 $\times \;10^{5}$	7.66 $\times \;10^{6}$	2.82 $\times \;10^{6}$
F18	Std	1.65 $\times \;10^{4}$	1.41 $\times \;10^{4}$	5.44 $\times \;10^{7}$	2.63 $\times \;10^{8}$	3.44 $\times \;10^{8}$	5.00 $\times \;10^{7}$	2.46 $\times \;10^{6}$	2.17 $\times \;10^{7}$	1.21 $\times \;10^{6}$	4.64 $\times \;10^{7}$	4.96 $\times \;10^{8}$	5.46 $\times \;10^{3}$	4.94 $\times \;10^{6}$	2.23 $\times \;10^{4}$
	Mean	1.64 $\times \;10^{4}$	1.16 $\times \;10^{4}$	9.55 $\times \;10^{7}$	2.62 $\times \;10^{8}$	6.87 $\times \;10^{8}$	2.89 $\times \;10^{7}$	1.58 $\times \;10^{6}$	4.62 $\times \;10^{6}$	1.47 $\times \;10^{6}$	4.10 $\times \;10^{7}$	7.67 $\times \;10^{8}$	6.09 $\times \;10^{3}$	6.42 $\times \;10^{6}$	2.72 $\times \;10^{4}$
F19	Std	2.33 $\times \;10^{2}$	3.72 $\times \;10^{2}$	1.52 $\times \;10^{2}$	2.47 $\times \;10^{2}$	1.34 $\times \;10^{2}$	1.66 $\times \;10^{2}$	2.31 $\times \;10^{2}$	1.91 $\times \;10^{2}$	2.21 $\times \;10^{2}$	1.65 $\times \;10^{2}$	2.20 $\times \;10^{2}$	1.45 $\times \;10^{2}$	1.94 $\times \;10^{2}$	1.84 $\times \;10^{2}$
	Mean	2.54 $\times \;10^{3}$	2.62 $\times \;10^{3}$	2.98 $\times \;10^{3}$	3.25 $\times \;10^{3}$	3.10 $\times \;10^{3}$	2.85 $\times \;10^{3}$	2.80 $\times \;10^{3}$	2.65 $\times \;10^{3}$	2.83 $\times \;10^{3}$	2.70 $\times \;10^{3}$	3.02 $\times \;10^{3}$	2.50 $\times \;10^{3}$	2.72 $\times \;10^{3}$	2.60 $\times \;10^{3}$
F20	Std	4.36 $\times \;10^{1}$	7.50 $\times \;10^{1}$	1.80 $\times \;10^{1}$	7.05 $\times \;10^{1}$	4.56 $\times \;10^{1}$	5.14 $\times \;10^{1}$	5.85 $\times \;10^{1}$	5.60 $\times \;10^{1}$	5.76 $\times \;10^{1}$	4.03 $\times \;10^{1}$	4.34 $\times \;10^{1}$	1.73 $\times \;10^{1}$	7.01 $\times \;10^{1}$	4.03 $\times \;10^{1}$
	Mean	2.42 $\times \;10^{3}$	2.47 $\times \;10^{3}$	2.61 $\times \;10^{3}$	2.84 $\times \;10^{3}$	2.73 $\times \;10^{3}$	2.60 $\times \;10^{3}$	2.56 $\times \;10^{3}$	2.56 $\times \;10^{3}$	2.60 $\times \;10^{3}$	2.60 $\times \;10^{3}$	2.76 $\times \;10^{3}$	2.48 $\times \;10^{3}$	2.55 $\times \;10^{3}$	2.42 $\times \;10^{3}$
F21	Std	2.46 $\times \;10^{3}$	3.97 $\times \;10^{3}$	2.02 $\times \;10^{3}$	1.31 $\times \;10^{3}$	9.86 $\times \;10^{2}$	2.84 $\times \;10^{3}$	2.51 $\times \;10^{3}$	1.55 $\times \;10^{3}$	1.47 $\times \;10^{3}$	1.30 $\times \;10^{3}$	6.22 $\times \;10^{2}$	3.79 $\times \;10^{0}$	2.35 $\times \;10^{3}$	1.53 $\times \;10^{3}$
	Mean	5.56 $\times \;10^{3}$	5.75 $\times \;10^{3}$	9.32 $\times \;10^{3}$	9.80 $\times \;10^{3}$	8.90 $\times \;10^{3}$	7.62 $\times \;10^{3}$	4.93 $\times \;10^{3}$	6.70 $\times \;10^{3}$	7.13 $\times \;10^{3}$	7.68 $\times \;10^{3}$	9.66 $\times \;10^{3}$	2.31 $\times \;10^{3}$	4.55 $\times \;10^{3}$	5.81 $\times \;10^{3}$
F22	Std	8.14 $\times \;10^{1}$	8.33 $\times \;10^{1}$	3.82 $\times \;10^{1}$	1.78 $\times \;10^{2}$	6.94 $\times \;10^{1}$	1.82 $\times \;10^{2}$	8.68 $\times \;10^{1}$	1.11 $\times \;10^{2}$	1.47 $\times \;10^{2}$	1.58 $\times \;10^{2}$	1.59 $\times \;10^{2}$	1.67 $\times \;10^{1}$	7.36 $\times \;10^{1}$	3.76 $\times \;10^{1}$
	Mean	2.85 $\times \;10^{3}$	2.80 $\times \;10^{3}$	3.08 $\times \;10^{3}$	3.65 $\times \;10^{3}$	3.33 $\times \;10^{3}$	3.30 $\times \;10^{3}$	3.03 $\times \;10^{3}$	3.12 $\times \;10^{3}$	3.24 $\times \;10^{3}$	3.48 $\times \;10^{3}$	3.66 $\times \;10^{3}$	2.85 $\times \;10^{3}$	3.06 $\times \;10^{3}$	2.77 $\times \;10^{3}$
F23	Std	8.13 $\times \;10^{1}$	8.82 $\times \;10^{1}$	3.51 $\times \;10^{1}$	2.62 $\times \;10^{2}$	7.87 $\times \;10^{1}$	1.93 $\times \;10^{2}$	6.78 $\times \;10^{1}$	1.77 $\times \;10^{2}$	1.60 $\times \;10^{2}$	1.76 $\times \;10^{2}$	1.77 $\times \;10^{2}$	2.15 $\times \;10^{1}$	8.33 $\times \;10^{1}$	4.06 $\times \;10^{1}$
	Mean	3.02 $\times \;10^{3}$	3.01 $\times \;10^{3}$	3.25 $\times \;10^{3}$	3.93 $\times \;10^{3}$	3.47 $\times \;10^{3}$	3.65 $\times \;10^{3}$	3.19 $\times \;10^{3}$	3.35 $\times \;10^{3}$	3.55 $\times \;10^{3}$	3.83 $\times \;10^{3}$	3.82 $\times \;10^{3}$	3.02 $\times \;10^{3}$	3.20 $\times \;10^{3}$	2.95 $\times \;10^{3}$
F24	Std	2.18 $\times \;10^{1}$	1.53 $\times \;10^{1}$	2.26 $\times \;10^{2}$	1.20 $\times \;10^{3}$	7.07 $\times \;10^{2}$	9.83 $\times \;10^{1}$	5.34 $\times \;10^{1}$	3.13 $\times \;10^{2}$	3.62 $\times \;10^{1}$	2.04 $\times \;10^{2}$	4.70 $\times \;10^{2}$	1.76 $\times \;10^{1}$	7.78 $\times \;10^{1}$	1.38 $\times \;10^{1}$
	Mean	2.90 $\times \;10^{3}$	2.90 $\times \;10^{3}$	3.60 $\times \;10^{3}$	6.18 $\times \;10^{3}$	4.84 $\times \;10^{3}$	3.27 $\times \;10^{3}$	2.99 $\times \;10^{3}$	3.14 $\times \;10^{3}$	3.02 $\times \;10^{3}$	3.94 $\times \;10^{3}$	5.18 $\times \;10^{3}$	2.92 $\times \;10^{3}$	3.11 $\times \;10^{3}$	2.90 $\times \;10^{3}$
F25	Std	1.25 $\times \;10^{3}$	8.60 $\times \;10^{2}$	5.22 $\times \;10^{2}$	1.08 $\times \;10^{3}$	9.74 $\times \;10^{2}$	1.35 $\times \;10^{3}$	6.58 $\times \;10^{2}$	1.63 $\times \;10^{3}$	1.37 $\times \;10^{3}$	9.09 $\times \;10^{2}$	7.97 $\times \;10^{2}$	9.97 $\times \;10^{2}$	1.38 $\times \;10^{3}$	4.61 $\times \;10^{2}$
	Mean	6.15 $\times \;10^{3}$	4.99 $\times \;10^{3}$	7.79 $\times \;10^{3}$	1.08 $\times \;10^{4}$	1.05 $\times \;10^{4}$	7.30 $\times \;10^{3}$	6.86 $\times \;10^{3}$	7.89 $\times \;10^{3}$	7.84 $\times \;10^{3}$	9.51 $\times \;10^{3}$	1.18 $\times \;10^{4}$	5.36 $\times \;10^{3}$	7.57 $\times \;10^{3}$	5.06 $\times \;10^{3}$
F26	Std	2.79 $\times \;10^{1}$	1.79 $\times \;10^{1}$	9.42 $\times \;10^{1}$	5.80 $\times \;10^{2}$	3.79 $\times \;10^{2}$	2.72 $\times \;10^{2}$	4.52 $\times \;10^{1}$	8.93 $\times \;10^{1}$	3.29 $\times \;10^{2}$	2.55 $\times \;10^{2}$	4.67 $\times \;10^{2}$	1.42 $\times \;10^{1}$	9.24 $\times \;10^{1}$	2.06 $\times \;10^{1}$
	Mean	3.28 $\times \;10^{3}$	3.23 $\times \;10^{3}$	3.58 $\times \;10^{3}$	4.49 $\times \;10^{3}$	3.95 $\times \;10^{3}$	3.65 $\times \;10^{3}$	3.32 $\times \;10^{3}$	3.39 $\times \;10^{3}$	3.66 $\times \;10^{3}$	4.25 $\times \;10^{3}$	4.51 $\times \;10^{3}$	3.28 $\times \;10^{3}$	3.41 $\times \;10^{3}$	3.23 $\times \;10^{3}$
F27	Std	4.65 $\times \;10^{1}$	2.13 $\times \;10^{1}$	4.52 $\times \;10^{2}$	1.39 $\times \;10^{3}$	7.00 $\times \;10^{2}$	1.18 $\times \;10^{3}$	7.50 $\times \;10^{2}$	7.72 $\times \;10^{2}$	9.13 $\times \;10^{1}$	6.74 $\times \;10^{2}$	6.58 $\times \;10^{2}$	2.85 $\times \;10^{1}$	9.08 $\times \;10^{1}$	4.43 $\times \;10^{1}$
	Mean	2.75 $\times \;10^{2}$	2.47 $\times \;10^{2}$	4.47 $\times \;10^{3}$	7.75 $\times \;10^{3}$	6.37 $\times \;10^{3}$	4.06 $\times \;10^{3}$	3.71 $\times \;10^{3}$	3.82 $\times \;10^{3}$	3.49 $\times \;10^{3}$	5.68 $\times \;10^{3}$	7.57 $\times \;10^{3}$	1.69 $\times \;10^{2}$	3.57 $\times \;10^{3}$	2.11 $\times \;10^{2}$
F28	Std	3.90 $\times \;10^{3}$	3.77 $\times \;10^{3}$	3.71 $\times \;10^{2}$	1.40 $\times \;10^{3}$	2.35 $\times \;10^{3}$	5.70 $\times \;10^{2}$	4.03 $\times \;10^{2}$	6.91 $\times \;10^{2}$	3.76 $\times \;10^{2}$	7.75 $\times \;10^{2}$	2.00 $\times \;10^{3}$	4.05 $\times \;10^{3}$	4.79 $\times \;10^{2}$	4.03 $\times \;10^{3}$
	Mean	4.07 $\times \;10^{3}$	3.79 $\times \;10^{3}$	5.29 $\times \;10^{3}$	6.68 $\times \;10^{3}$	7.28 $\times \;10^{3}$	4.81 $\times \;10^{3}$	4.46 $\times \;10^{3}$	4.90 $\times \;10^{3}$	4.97 $\times \;10^{3}$	6.13 $\times \;10^{3}$	8.59 $\times \;10^{3}$	4.00 $\times \;10^{3}$	5.01 $\times \;10^{3}$	4.03 $\times \;10^{3}$
F29	Std	9.59 $\times \;10^{3}$	5.73 $\times \;10^{3}$	8.34 $\times \;10^{7}$	3.68 $\times \;10^{8}$	1.14 $\times \;10^{9}$	5.13 $\times \;10^{7}$	5.02 $\times \;10^{6}$	4.98 $\times \;10^{7}$	9.39 $\times \;10^{6}$	4.74 $\times \;10^{8}$	9.10 $\times \;10^{8}$	7.81 $\times \;10^{4}$	4.22 $\times \;10^{7}$	8.46 $\times \;10^{4}$
	Mean	1.70 $\times \;10^{4}$	1.33 $\times \;10^{4}$	2.03 $\times \;10^{8}$	2.98 $\times \;10^{8}$	3.00 $\times \;10^{9}$	2.99 $\times \;10^{7}$	2.90 $\times \;10^{6}$	1.90 $\times \;10^{7}$	1.13 $\times \;10^{7}$	5.87 $\times \;10^{8}$	1.52 $\times \;10^{9}$	1.35 $\times \;10^{5}$	6.12 $\times \;10^{7}$	1.17 $\times \;10^{5}$

**Table A2:** Comparison of the FA-BCA with other algorithms on IEEE CEC 2017 benchmark functions with D = 50.

F	Item	FBCA	BCA	SCA	GA	RSA	PSO	DBO	BKA	HHO	HOA	COA	CPO	PO	SMA
F1	Std	2.73 $\times \;10^{5}$	8.68 $\times \;10^{6}$	8.35 $\times \;10^{9}$	2.54 $\times \;10^{10}$	7.82 $\times \;10^{9}$	5.19 $\times \;10^{9}$	1.95 $\times \;10^{10}$	2.11 $\times \;10^{10}$	1.81 $\times \;10^{9}$	8.39 $\times \;10^{9}$	6.1 $\times \;10^{9}$	1.43 $\times \;10^{8}$	4.08 $\times \;10^{9}$	2.26 $\times \;10^{6}$
	Mean	4.95 $\times \;10^{5}$	1.06 $\times \;10^{7}$	6.58 $\times \;10^{10}$	1.69 $\times \;10^{11}$	10.00 $\times \;10^{10}$	1.90 $\times \;10^{10}$	1.02 $\times \;10^{10}$	4.44 $\times \;10^{10}$	5.25 $\times \;10^{9}$	8.54 $\times \;10^{10}$	1.15 $\times \;10^{11}$	1.88 $\times \;10^{8}$	1.58 $\times \;10^{10}$	6.55 $\times \;10^{6}$
F2	Std	6.26 $\times \;10^{4}$	7.47 $\times \;10^{4}$	3.19 $\times \;10^{4}$	9.81 $\times \;10^{4}$	1.57 $\times \;10^{4}$	1.27 $\times \;10^{5}$	6.75 $\times \;10^{4}$	2.84 $\times \;10^{4}$	1.96 $\times \;10^{4}$	1.39 $\times \;10^{4}$	2.07 $\times \;10^{4}$	1.99 $\times \;10^{4}$	2.94 $\times \;10^{4}$	6.25 $\times \;10^{4}$
	Mean	2.34 $\times \;10^{5}$	3.26 $\times \;10^{5}$	2.20 $\times \;10^{5}$	4.68 $\times \;10^{5}$	1.77 $\times \;10^{5}$	3.96 $\times \;10^{5}$	2.69 $\times \;10^{5}$	9.58 $\times \;10^{4}$	1.72 $\times \;10^{5}$	1.59 $\times \;10^{5}$	2.01 $\times \;10^{5}$	1.78 $\times \;10^{5}$	1.94 $\times \;10^{5}$	1.82 $\times \;10^{5}$
F3	Std	6.55 $\times \;10^{1}$	5.72 $\times \;10^{1}$	3.50 $\times \;10^{3}$	1.57 $\times \;10^{4}$	5.72 $\times \;10^{3}$	2.43 $\times \;10^{3}$	7.37 $\times \;10^{2}$	6.84 $\times \;10^{3}$	6.01 $\times \;10^{2}$	5.01 $\times \;10^{3}$	5.17 $\times \;10^{3}$	4.94 $\times \;10^{1}$	9.82 $\times \;10^{2}$	6.74 $\times \;10^{1}$
	Mean	6.08 $\times \;10^{2}$	6.29 $\times \;10^{2}$	1.45 $\times \;10^{4}$	4.46 $\times \;10^{4}$	2.92 $\times \;10^{4}$	3.60 $\times \;10^{3}$	1.43 $\times \;10^{3}$	7.39 $\times \;10^{3}$	2.00 $\times \;10^{3}$	2.38 $\times \;10^{4}$	3.81 $\times \;10^{4}$	7.01 $\times \;10^{2}$	2.67 $\times \;10^{3}$	6.31 $\times \;10^{2}$
F4	Std	8.03 $\times \;10^{1}$	1.57 $\times \;10^{2}$	3.34 $\times \;10^{1}$	9.83 $\times \;10^{1}$	3.08 $\times \;10^{1}$	5.03 $\times \;10^{1}$	1.05 $\times \;10^{2}$	7.48 $\times \;10^{1}$	3.30 $\times \;10^{1}$	4.20 $\times \;10^{1}$	3.11 $\times \;10^{1}$	2.17 $\times \;10^{1}$	4.94 $\times \;10^{1}$	5.01 $\times \;10^{1}$
	Mean	7.88 $\times \;10^{2}$	8.48 $\times \;10^{2}$	1.14 $\times \;10^{3}$	1.51 $\times \;10^{3}$	1.17 $\times \;10^{3}$	1.11 $\times \;10^{3}$	9.88 $\times \;10^{2}$	9.07 $\times \;10^{2}$	9.43 $\times \;10^{2}$	1.08 $\times \;10^{3}$	1.19 $\times \;10^{3}$	9.29 $\times \;10^{2}$	1.02 $\times \;10^{3}$	8.16 $\times \;10^{2}$
F5	Std	6.69 $\times \;10^{0}$	2.75 $\times \;10^{0}$	6.21 $\times \;10^{0}$	1.35 $\times \;10^{1}$	4.90 $\times \;10^{0}$	1.55 $\times \;10^{1}$	1.22 $\times \;10^{1}$	9.83 $\times \;10^{0}$	5.00 $\times \;10^{0}$	6.97 $\times \;10^{0}$	2.35 $\times \;10^{0}$	3.19 $\times \;10^{0}$	7.41 $\times \;10^{0}$	1.18 $\times \;10^{1}$
	Mean	6.26 $\times \;10^{2}$	6.07 $\times \;10^{2}$	6.84 $\times \;10^{2}$	7.39 $\times \;10^{2}$	7.04 $\times \;10^{2}$	6.79 $\times \;10^{2}$	6.66 $\times \;10^{2}$	6.71 $\times \;10^{2}$	6.80 $\times \;10^{2}$	6.85 $\times \;10^{2}$	7.04 $\times \;10^{2}$	6.11 $\times \;10^{2}$	6.83 $\times \;10^{2}$	6.47 $\times \;10^{2}$
F6	Std	1.78 $\times \;10^{2}$	9.88 $\times \;10^{1}$	1.07 $\times \;10^{2}$	6.29 $\times \;10^{2}$	5.82 $\times \;10^{1}$	1.04 $\times \;10^{2}$	1.47 $\times \;10^{2}$	9.43 $\times \;10^{1}$	9.91 $\times \;10^{1}$	7.00 $\times \;10^{1}$	4.47 $\times \;10^{1}$	4.36 $\times \;10^{1}$	1.03 $\times \;10^{2}$	1.01 $\times \;10^{2}$
	Mean	1.35 $\times \;10^{3}$	1.28 $\times \;10^{3}$	1.91 $\times \;10^{3}$	4.50 $\times \;10^{3}$	1.96 $\times \;10^{3}$	1.73 $\times \;10^{3}$	1.41 $\times \;10^{3}$	1.76 $\times \;10^{3}$	1.88 $\times \;10^{3}$	1.85 $\times \;10^{3}$	2.06 $\times \;10^{3}$	1.26 $\times \;10^{3}$	1.79 $\times \;10^{3}$	1.17 $\times \;10^{3}$
F7	Std	6.34 $\times \;10^{1}$	1.34 $\times \;10^{2}$	3.22 $\times \;10^{1}$	1.01 $\times \;10^{2}$	2.15 $\times \;10^{1}$	6.37 $\times \;10^{1}$	9.63 $\times \;10^{1}$	1.09 $\times \;10^{2}$	3.82 $\times \;10^{1}$	4.27 $\times \;10^{1}$	2.97 $\times \;10^{1}$	2.25 $\times \;10^{1}$	5.95 $\times \;10^{1}$	5.27 $\times \;10^{1}$
	Mean	1.08 $\times \;10^{3}$	1.20 $\times \;10^{3}$	1.44 $\times \;10^{3}$	1.79 $\times \;10^{3}$	1.51 $\times \;10^{3}$	1.41 $\times \;10^{3}$	1.31 $\times \;10^{3}$	1.26 $\times \;10^{3}$	1.24 $\times \;10^{3}$	1.43 $\times \;10^{3}$	1.50 $\times \;10^{3}$	1.22 $\times \;10^{3}$	1.34 $\times \;10^{3}$	1.10 $\times \;10^{3}$
F8	Std	1.01 $\times \;10^{4}$	3.92 $\times \;10^{3}$	4.25 $\times \;10^{3}$	6.13 $\times \;10^{3}$	2.18 $\times \;10^{3}$	8.53 $\times \;10^{3}$	7.80 $\times \;10^{3}$	4.59 $\times \;10^{3}$	2.69 $\times \;10^{3}$	3.55 $\times \;10^{3}$	2.75 $\times \;10^{3}$	2.57 $\times \;10^{3}$	4.89 $\times \;10^{3}$	3.46 $\times \;10^{3}$
	Mean	1.99 $\times \;10^{4}$	6.73 $\times \;10^{3}$	3.38 $\times \;10^{4}$	4.84 $\times \;10^{4}$	3.91 $\times \;10^{4}$	2.93 $\times \;10^{4}$	2.96 $\times \;10^{4}$	1.74 $\times \;10^{4}$	3.23 $\times \;10^{4}$	2.82 $\times \;10^{4}$	3.77 $\times \;10^{4}$	7.83 $\times \;10^{3}$	2.74 $\times \;10^{4}$	1.70 $\times \;10^{4}$
F9	Std	3.40 $\times \;10^{3}$	9.01 $\times \;10^{2}$	3.83 $\times \;10^{2}$	9.06 $\times \;10^{2}$	4.83 $\times \;10^{2}$	1.00 $\times \;10^{3}$	2.18 $\times \;10^{3}$	1.61 $\times \;10^{3}$	1.16 $\times \;10^{3}$	7.75 $\times \;10^{2}$	3.76 $\times \;10^{2}$	5.37 $\times \;10^{2}$	1.02 $\times \;10^{3}$	1.01 $\times \;10^{3}$
	Mean	8.88 $\times \;10^{3}$	1.57 $\times \;10^{4}$	1.54 $\times \;10^{4}$	1.52 $\times \;10^{4}$	1.52 $\times \;10^{4}$	1.42 $\times \;10^{4}$	1.17 $\times \;10^{4}$	9.47 $\times \;10^{3}$	1.06 $\times \;10^{4}$	1.31 $\times \;10^{4}$	1.55 $\times \;10^{4}$	1.36 $\times \;10^{4}$	1.24 $\times \;10^{4}$	8.30 $\times \;10^{3}$
F10	Std	1.59 $\times \;10^{3}$	1.12 $\times \;10^{3}$	3.11 $\times \;10^{3}$	2.10 $\times \;10^{4}$	2.25 $\times \;10^{3}$	1.26 $\times \;10^{4}$	3.09 $\times \;10^{3}$	4.58 $\times \;10^{3}$	7.43 $\times \;10^{2}$	2.27 $\times \;10^{3}$	2.44 $\times \;10^{3}$	2.77 $\times \;10^{2}$	2.60 $\times \;10^{3}$	7.39 $\times \;10^{1}$
	Mean	2.06 $\times \;10^{3}$	2.31 $\times \;10^{3}$	1.26 $\times \;10^{4}$	6.26 $\times \;10^{4}$	2.18 $\times \;10^{4}$	1.93 $\times \;10^{4}$	4.19 $\times \;10^{3}$	5.63 $\times \;10^{3}$	3.10 $\times \;10^{3}$	1.89 $\times \;10^{4}$	2.67 $\times \;10^{4}$	1.84 $\times \;10^{3}$	7.94 $\times \;10^{3}$	1.44 $\times \;10^{3}$
F11	Std	7.59 $\times \;10^{6}$	5.38 $\times \;10^{6}$	6.77 $\times \;10^{9}$	2.59 $\times \;10^{10}$	1.95 $\times \;10^{10}$	7.50 $\times \;10^{9}$	5.78 $\times \;10^{8}$	1.56 $\times \;10^{10}$	6.82 $\times \;10^{8}$	1.09 $\times \;10^{10}$	1.36 $\times \;10^{10}$	1.05 $\times \;10^{7}$	1.19 $\times \;10^{9}$	1.92 $\times \;10^{7}$
	Mean	1.29 $\times \;10^{7}$	8.83 $\times \;10^{6}$	2.24 $\times \;10^{10}$	6.73 $\times \;10^{10}$	7.46 $\times \;10^{10}$	9.63 $\times \;10^{9}$	8.85 $\times \;10^{8}$	1.25 $\times \;10^{10}$	1.03 $\times \;10^{9}$	5.24 $\times \;10^{10}$	9.30 $\times \;10^{10}$	2.01 $\times \;10^{7}$	2.54 $\times \;10^{9}$	3.84 $\times \;10^{7}$
F12	Std	8.06 $\times \;10^{3}$	7.78 $\times \;10^{3}$	3.15 $\times \;10^{9}$	1.56 $\times \;10^{10}$	1.50 $\times \;10^{10}$	6.04 $\times \;10^{9}$	1.37 $\times \;10^{8}$	5.49 $\times \;10^{9}$	6.32 $\times \;10^{7}$	8.06 $\times \;10^{9}$	1.29 $\times \;10^{10}$	3.46 $\times \;10^{4}$	2.13 $\times \;10^{8}$	8.08 $\times \;10^{4}$
	Mean	1.44 $\times \;10^{4}$	9.44 $\times \;10^{3}$	7.19 $\times \;10^{9}$	2.91 $\times \;10^{10}$	4.80 $\times \;10^{10}$	5.83 $\times \;10^{9}$	9.34 $\times \;10^{7}$	1.71 $\times \;10^{9}$	4.08 $\times \;10^{7}$	2.24 $\times \;10^{10}$	5.38 $\times \;10^{10}$	2.58 $\times \;10^{4}$	3.47 $\times \;10^{8}$	1.32 $\times \;10^{5}$
F13	Std	8.22 $\times \;10^{5}$	6.68 $\times \;10^{5}$	5.86 $\times \;10^{6}$	1.30 $\times \;10^{8}$	5.40 $\times \;10^{7}$	4.76 $\times \;10^{7}$	3.85 $\times \;10^{6}$	1.04 $\times \;10^{5}$	7.33 $\times \;10^{6}$	2.99 $\times \;10^{7}$	9.57 $\times \;10^{7}$	1.66 $\times \;10^{5}$	2.87 $\times \;10^{6}$	7.45 $\times \;10^{5}$
	Mean	6.52 $\times \;10^{5}$	7.46 $\times \;10^{5}$	8.64 $\times \;10^{6}$	9.82 $\times \;10^{7}$	7.07 $\times \;10^{7}$	2.13 $\times \;10^{7}$	3.62 $\times \;10^{6}$	1.35 $\times \;10^{5}$	6.22 $\times \;10^{6}$	4.69 $\times \;10^{7}$	1.18 $\times \;10^{8}$	1.31 $\times \;10^{5}$	4.94 $\times \;10^{6}$	9.28 $\times \;10^{5}$
F14	Std	8.19 $\times \;10^{3}$	6.77 $\times \;10^{3}$	4.72 $\times \;10^{8}$	4.41 $\times \;10^{9}$	2.46 $\times \;10^{9}$	8.04 $\times \;10^{7}$	7.61 $\times \;10^{7}$	8.93 $\times \;10^{8}$	5.69 $\times \;10^{6}$	2.35 $\times \;10^{9}$	4.17 $\times \;10^{9}$	1.22 $\times \;10^{4}$	2.39 $\times \;10^{7}$	1.94 $\times \;10^{4}$
	Mean	1.08 $\times \;10^{4}$	8.76 $\times \;10^{3}$	1.15 $\times \;10^{9}$	7.82 $\times \;10^{9}$	5.96 $\times \;10^{9}$	7.06 $\times \;10^{7}$	2.03 $\times \;10^{7}$	2.17 $\times \;10^{8}$	3.32 $\times \;10^{6}$	4.43 $\times \;10^{9}$	1.03 $\times \;10^{10}$	1.44 $\times \;10^{4}$	1.83 $\times \;10^{7}$	4.98 $\times \;10^{4}$
F15	Std	7.32 $\times \;10^{2}$	1.13 $\times \;10^{3}$	3.58 $\times \;10^{2}$	1.29 $\times \;10^{3}$	1.58 $\times \;10^{3}$	9.60 $\times \;10^{2}$	6.64 $\times \;10^{2}$	1.10 $\times \;10^{3}$	8.06 $\times \;10^{2}$	9.78 $\times \;10^{2}$	1.39 $\times \;10^{3}$	2.69 $\times \;10^{2}$	7.42 $\times \;10^{2}$	4.05 $\times \;10^{2}$
	Mean	3.68 $\times \;10^{3}$	4.86 $\times \;10^{3}$	6.26 $\times \;10^{3}$	7.85 $\times \;10^{3}$	8.33 $\times \;10^{3}$	5.84 $\times \;10^{3}$	4.93 $\times \;10^{3}$	4.46 $\times \;10^{3}$	4.95 $\times \;10^{3}$	7.08 $\times \;10^{3}$	1.06 $\times \;10^{4}$	4.53 $\times \;10^{3}$	5.56 $\times \;10^{3}$	3.70 $\times \;10^{3}$
F16	Std	3.43 $\times \;10^{2}$	4.92 $\times \;10^{2}$	3.13 $\times \;10^{2}$	4.79 $\times \;10^{4}$	4.73 $\times \;10^{3}$	4.46 $\times \;10^{2}$	4.82 $\times \;10^{2}$	7.39 $\times \;10^{2}$	2.98 $\times \;10^{2}$	5.67 $\times \;10^{2}$	6.02 $\times \;10^{3}$	2.36 $\times \;10^{2}$	4.22 $\times \;10^{2}$	2.92 $\times \;10^{2}$
	Mean	3.17 $\times \;10^{3}$	4.17 $\times \;10^{3}$	5.05 $\times \;10^{3}$	2.32 $\times \;10^{4}$	1.34 $\times \;10^{4}$	4.58 $\times \;10^{3}$	4.40 $\times \;10^{3}$	3.82 $\times \;10^{3}$	3.90 $\times \;10^{3}$	4.84 $\times \;10^{3}$	1.16 $\times \;10^{4}$	3.47 $\times \;10^{3}$	4.22 $\times \;10^{3}$	3.35 $\times \;10^{3}$
F17	Std	2.30 $\times \;10^{6}$	1.32 $\times \;10^{7}$	3.07 $\times \;10^{7}$	1.48 $\times \;10^{8}$	8.23 $\times \;10^{7}$	3.99 $\times \;10^{7}$	1.61 $\times \;10^{7}$	1.14 $\times \;10^{7}$	1.40 $\times \;10^{7}$	4.56 $\times \;10^{7}$	1.00 $\times \;10^{8}$	9.88 $\times \;10^{5}$	2.63 $\times \;10^{7}$	5.40 $\times \;10^{6}$
	Mean	3.61 $\times \;10^{6}$	1.09 $\times \;10^{7}$	6.09 $\times \;10^{7}$	1.59 $\times \;10^{8}$	1.91 $\times \;10^{8}$	2.66 $\times \;10^{7}$	1.37 $\times \;10^{7}$	5.01 $\times \;10^{6}$	1.13 $\times \;10^{7}$	1.00 $\times \;10^{8}$	2.02 $\times \;10^{8}$	1.7 $\times \;10^{6}$	3.66 $\times \;10^{7}$	7.09 $\times \;10^{6}$
F18	Std	1.45 $\times \;10^{4}$	1.30 $\times \;10^{4}$	3.91 $\times \;10^{8}$	2.21 $\times \;10^{9}$	1.44 $\times \;10^{9}$	3.37 $\times \;10^{8}$	1.28 $\times \;10^{7}$	8.63 $\times \;10^{7}$	2.50 $\times \;10^{6}$	7.18 $\times \;10^{8}$	1.51 $\times \;10^{9}$	7.71 $\times \;10^{3}$	1.60 $\times \;10^{7}$	1.56 $\times \;10^{4}$
	Mean	2.60 $\times \;10^{4}$	1.62 $\times \;10^{4}$	7.85 $\times \;10^{8}$	3.44 $\times \;10^{9}$	4.68 $\times \;10^{9}$	1.33 $\times \;10^{8}$	9.62 $\times \;10^{6}$	1.69 $\times \;10^{7}$	2.27 $\times \;10^{6}$	1.00 $\times \;10^{9}$	3.19 $\times \;10^{9}$	2.21 $\times \;10^{4}$	2.21 $\times \;10^{7}$	2.30 $\times \;10^{4}$
F19	Std	5.27 $\times \;10^{2}$	5.22 $\times \;10^{2}$	2.21 $\times \;10^{2}$	3.47 $\times \;10^{2}$	1.69 $\times \;10^{2}$	3.12 $\times \;10^{2}$	3.76 $\times \;10^{2}$	3.40 $\times \;10^{2}$	2.74 $\times \;10^{2}$	2.85 $\times \;10^{2}$	1.85 $\times \;10^{2}$	2.34 $\times \;10^{2}$	3.76 $\times \;10^{2}$	2.24 $\times \;10^{2}$
	Mean	3.28 $\times \;10^{3}$	4.21 $\times \;10^{3}$	4.36 $\times \;10^{3}$	4.79 $\times \;10^{3}$	4.25 $\times \;10^{3}$	4.14 $\times \;10^{3}$	3.82 $\times \;10^{3}$	3.29 $\times \;10^{3}$	3.65 $\times \;10^{3}$	3.75 $\times \;10^{3}$	4.15 $\times \;10^{3}$	3.58 $\times \;10^{3}$	3.70 $\times \;10^{3}$	3.32 $\times \;10^{3}$
F20	Std	7.19 $\times \;10^{1}$	1.49 $\times \;10^{2}$	3.84 $\times \;10^{1}$	1.31 $\times \;10^{2}$	7.90 $\times \;10^{1}$	7.72 $\times \;10^{1}$	9.81 $\times \;10^{1}$	1.18 $\times \;10^{2}$	9.74 $\times \;10^{1}$	6.23 $\times \;10^{1}$	8.97 $\times \;10^{1}$	2.67 $\times \;10^{1}$	7.90 $\times \;10^{1}$	6.36 $\times \;10^{1}$
	Mean	2.56 $\times \;10^{3}$	2.65 $\times \;10^{3}$	2.96 $\times \;10^{3}$	3.38 $\times \;10^{3}$	3.12 $\times \;10^{3}$	2.93 $\times \;10^{3}$	2.85 $\times \;10^{3}$	2.88 $\times \;10^{3}$	2.98 $\times \;10^{3}$	2.98 $\times \;10^{3}$	3.31 $\times \;10^{3}$	2.70 $\times \;10^{3}$	2.90 $\times \;10^{3}$	2.58 $\times \;10^{3}$
F21	Std	3.32 $\times \;10^{3}$	2.88 $\times \;10^{3}$	4.53 $\times \;10^{2}$	7.90 $\times \;10^{2}$	5.24 $\times \;10^{2}$	2.17 $\times \;10^{3}$	2.27 $\times \;10^{3}$	1.22 $\times \;10^{3}$	1.09 $\times \;10^{3}$	8.03 $\times \;10^{2}$	6.09 $\times \;10^{2}$	5.62 $\times \;10^{3}$	9.99 $\times \;10^{2}$	8.72 $\times \;10^{2}$
	Mean	1.17 $\times \;10^{4}$	1.67 $\times \;10^{4}$	1.73 $\times \;10^{4}$	1.75 $\times \;10^{4}$	1.74 $\times \;10^{4}$	1.51 $\times \;10^{4}$	1.29 $\times \;10^{4}$	1.11 $\times \;10^{4}$	1.25 $\times \;10^{4}$	1.52 $\times \;10^{4}$	1.69 $\times \;10^{4}$	1.19 $\times \;10^{4}$	1.42 $\times \;10^{4}$	9.77 $\times \;10^{3}$
F22	Std	1.23 $\times \;10^{2}$	1.68 $\times \;10^{2}$	9.17 $\times \;10^{1}$	3.20 $\times \;10^{2}$	1.90 $\times \;10^{2}$	3.54 $\times \;10^{2}$	1.58 $\times \;10^{2}$	1.67 $\times \;10^{2}$	2.32 $\times \;10^{2}$	2.67 $\times \;10^{2}$	2.23 $\times \;10^{2}$	3.02 $\times \;10^{1}$	2.03 $\times \;10^{2}$	6.20 $\times \;10^{1}$
	Mean	3.13 $\times \;10^{3}$	3.09 $\times \;10^{3}$	3.73 $\times \;10^{3}$	4.65 $\times \;10^{3}$	4.08 $\times \;10^{3}$	4.14 $\times \;10^{3}$	3.61 $\times \;10^{3}$	3.78 $\times \;10^{3}$	4.04 $\times \;10^{3}$	4.52 $\times \;10^{3}$	4.60 $\times \;10^{3}$	3.18 $\times \;10^{3}$	3.67 $\times \;10^{3}$	3.03 $\times \;10^{3}$
F23	Std	1.22 $\times \;10^{2}$	1.21 $\times \;10^{2}$	9.37 $\times \;10^{1}$	3.67 $\times \;10^{2}$	6.44 $\times \;10^{2}$	3.01 $\times \;10^{2}$	1.56 $\times \;10^{2}$	2.26 $\times \;10^{2}$	2.47 $\times \;10^{2}$	2.61 $\times \;10^{2}$	2.27 $\times \;10^{2}$	3.12 $\times \;10^{1}$	1.17 $\times \;10^{2}$	1.03 $\times \;10^{2}$
	Mean	3.31 $\times \;10^{3}$	3.36 $\times \;10^{3}$	3.88 $\times \;10^{3}$	5.16 $\times \;10^{3}$	4.42 $\times \;10^{3}$	4.63 $\times \;10^{3}$	3.71 $\times \;10^{3}$	3.90 $\times \;10^{3}$	4.47 $\times \;10^{3}$	5.00 $\times \;10^{3}$	4.88 $\times \;10^{3}$	3.35 $\times \;10^{3}$	3.80 $\times \;10^{3}$	3.19 $\times \;10^{3}$
F24	Std	3.38 $\times \;10^{1}$	3.63 $\times \;10^{1}$	1.20 $\times \;10^{3}$	7.21 $\times \;10^{3}$	1.42 $\times \;10^{3}$	1.00 $\times \;10^{3}$	1.70 $\times \;10^{3}$	2.40 $\times \;10^{3}$	2.45 $\times \;10^{2}$	9.93 $\times \;10^{2}$	1.07 $\times \;10^{3}$	4.87 $\times \;10^{1}$	4.58 $\times \;10^{2}$	4.22 $\times \;10^{1}$
	Mean	3.08 $\times \;10^{3}$	3.11 $\times \;10^{3}$	9.68 $\times \;10^{3}$	2.99 $\times \;10^{4}$	1.34 $\times \;10^{4}$	5.85 $\times \;10^{3}$	3.91 $\times \;10^{3}$	6.13 $\times \;10^{3}$	3.83 $\times \;10^{3}$	1.19 $\times \;10^{4}$	1.56 $\times \;10^{4}$	3.23 $\times \;10^{3}$	4.51 $\times \;10^{3}$	3.10 $\times \;10^{3}$
F25	Std	1.65 $\times \;10^{3}$	1.61 $\times \;10^{3}$	7.94 $\times \;10^{2}$	2.33 $\times \;10^{3}$	7.26 $\times \;10^{2}$	2.19 $\times \;10^{3}$	1.60 $\times \;10^{3}$	2.20 $\times \;10^{3}$	1.40 $\times \;10^{3}$	7.39 $\times \;10^{2}$	6.25 $\times \;10^{2}$	1.74 $\times \;10^{3}$	2.34 $\times \;10^{3}$	2.28 $\times \;10^{3}$
	Mean	8.06 $\times \;10^{3}$	7.33 $\times \;10^{3}$	1.41 $\times \;10^{4}$	2.13 $\times \;10^{4}$	1.65 $\times \;10^{4}$	1.27 $\times \;10^{4}$	1.13 $\times \;10^{4}$	1.25 $\times \;10^{4}$	1.19 $\times \;10^{4}$	1.55 $\times \;10^{4}$	1.76 $\times \;10^{4}$	7.99 $\times \;10^{3}$	1.14 $\times \;10^{4}$	4.87 $\times \;10^{3}$
F26	Std	1.39 $\times \;10^{2}$	8.58 $\times \;10^{1}$	2.52 $\times \;10^{2}$	7.19 $\times \;10^{2}$	1.13 $\times \;10^{3}$	8.53 $\times \;10^{2}$	3.16 $\times \;10^{2}$	5.00 $\times \;10^{2}$	5.65 $\times \;10^{2}$	5.97 $\times \;10^{2}$	8.51 $\times \;10^{2}$	7.25 $\times \;10^{1}$	2.90 $\times \;10^{2}$	8.15 $\times \;10^{1}$
	Mean	3.65 $\times \;10^{3}$	3.47 $\times \;10^{3}$	4.97 $\times \;10^{3}$	6.98 $\times \;10^{3}$	6.14 $\times \;10^{3}$	5.12 $\times \;10^{3}$	4.04 $\times \;10^{3}$	4.35 $\times \;10^{3}$	5.21 $\times \;10^{3}$	6.98 $\times \;10^{3}$	7.14 $\times \;10^{3}$	3.74 $\times \;10^{3}$	4.25 $\times \;10^{3}$	3.55 $\times \;10^{3}$
F27	Std	4.18 $\times \;10^{1}$	8.34 $\times \;10^{1}$	1.10 $\times \;10^{3}$	3.23 $\times \;10^{3}$	1.28 $\times \;10^{3}$	1.60 $\times \;10^{3}$	2.38 $\times \;10^{3}$	2.26 $\times \;10^{3}$	3.99 $\times \;10^{2}$	8.84 $\times \;10^{2}$	1.63 $\times \;10^{3}$	1.24 $\times \;10^{2}$	4.19 $\times \;10^{2}$	4.88 $\times \;10^{1}$
	Mean	3.39 $\times \;10^{3}$	3.45 $\times \;10^{3}$	8.69 $\times \;10^{3}$	1.60 $\times \;10^{4}$	1.17 $\times \;10^{4}$	6.50 $\times \;10^{3}$	6.32 $\times \;10^{3}$	6.82 $\times \;10^{3}$	4.81 $\times \;10^{3}$	1.07 $\times \;10^{4}$	1.37 $\times \;10^{4}$	3.70 $\times \;10^{3}$	5.32 $\times \;10^{3}$	3.38 $\times \;10^{3}$
F28	Std	3.67 $\times \;10^{2}$	8.21 $\times \;10^{2}$	9.39 $\times \;10^{2}$	6.29 $\times \;10^{4}$	8.47 $\times \;10^{4}$	9.26 $\times \;10^{3}$	8.47 $\times \;10^{2}$	5.28 $\times \;10^{3}$	8.38 $\times \;10^{2}$	2.20 $\times \;10^{4}$	1.24 $\times \;10^{5}$	2.50 $\times \;10^{2}$	1.69 $\times \;10^{3}$	4.67 $\times \;10^{2}$
	Mean	4.52 $\times \;10^{3}$	4.65 $\times \;10^{3}$	8.86 $\times \;10^{3}$	3.31 $\times \;10^{4}$	6.00 $\times \;10^{4}$	9.51 $\times \;10^{3}$	6.45 $\times \;10^{3}$	8.27 $\times \;10^{3}$	6.99 $\times \;10^{3}$	2.26 $\times \;10^{4}$	1.33 $\times \;10^{5}$	5.16 $\times \;10^{3}$	8.16 $\times \;10^{3}$	4.88 $\times \;10^{3}$
F29	Std	4.56 $\times \;10^{5}$	4.39 $\times \;10^{5}$	5.00 $\times \;10^{8}$	2.99 $\times \;10^{9}$	2.40 $\times \;10^{9}$	6.39 $\times \;10^{8}$	4.60 $\times \;10^{7}$	6.15 $\times \;10^{7}$	3.83 $\times \;10^{7}$	1.21 $\times \;10^{9}$	2.92 $\times \;10^{9}$	3.10 $\times \;10^{6}$	1.27 $\times \;10^{8}$	4.43 $\times \;10^{6}$
	Mean	1.75 $\times \;10^{6}$	1.2 $\times \;10^{6}$	1.42 $\times \;10^{9}$	5.64 $\times \;10^{9}$	7.92 $\times \;10^{9}$	5.30 $\times \;10^{8}$	5.18 $\times \;10^{7}$	7.25 $\times \;10^{7}$	1.34 $\times \;10^{8}$	3.02 $\times \;10^{9}$	8.81 $\times \;10^{9}$	9.53 $\times \;10^{6}$	3.12 $\times \;10^{8}$	1.28 $\times \;10^{7}$

**Table A3:** Comparison of the FA-BCA with other algorithms on IEEE CEC 2017 benchmark functions with D = 100.

F	Item	FBCA	BCA	SCA	GA	RSA	PSO	DBO	BKA	HHO	HOA	COA	CPO	PO	SMA
F1	Std	1.87 $\times \;10^{8}$	7.9 $\times \;10^{9}$	1.31 $\times \;10^{10}$	7.1 $\times \;10^{10}$	6.9 $\times \;10^{9}$	1.88 $\times \;10^{10}$	7.29 $\times \;10^{10}$	4.57 $\times \;10^{10}$	7.91 $\times \;10^{9}$	1.27 $\times \;10^{10}$	1.21 $\times \;10^{10}$	3.26 $\times \;10^{9}$	1.09 $\times \;10^{10}$	9.06 $\times \;10^{7}$
	Mean	2.00 $\times \;10^{8}$	1.08 $\times \;10^{10}$	2.19 $\times \;10^{11}$	5.55 $\times \;10^{11}$	2.46 $\times \;10^{11}$	1.16 $\times \;10^{11}$	8.25 $\times \;10^{10}$	1.53 $\times \;10^{11}$	5.15 $\times \;10^{10}$	2.25 $\times \;10^{11}$	2.72 $\times \;10^{11}$	1.40 $\times \;10^{10}$	8.96 $\times \;10^{10}$	4.70 $\times \;10^{8}$
F2	Std	9.09 $\times \;10^{4}$	2.65 $\times \;10^{5}$	9.83 $\times \;10^{4}$	1.47 $\times \;10^{5}$	1.82 $\times \;10^{4}$	2.72 $\times \;10^{5}$	2.23 $\times \;10^{5}$	4.75 $\times \;10^{4}$	2.03 $\times \;10^{5}$	1.07 $\times \;10^{4}$	1.37 $\times \;10^{4}$	6.25 $\times \;10^{4}$	6.67 $\times \;10^{4}$	3.19 $\times \;10^{5}$
	Mean	6.97 $\times \;10^{5}$	8.59 $\times \;10^{5}$	6.20 $\times \;10^{5}$	9.21 $\times \;10^{5}$	3.48 $\times \;10^{5}$	1.02 $\times \;10^{6}$	6.16 $\times \;10^{5}$	2.72 $\times \;10^{5}$	4.12 $\times \;10^{5}$	3.28 $\times \;10^{5}$	3.55 $\times \;10^{5}$	4.60 $\times \;10^{5}$	3.91 $\times \;10^{5}$	7.78 $\times \;10^{5}$
F3	Std	1.01 $\times \;10^{2}$	4.73 $\times \;10^{2}$	8.51 $\times \;10^{3}$	4.82 $\times \;10^{4}$	1.31 $\times \;10^{4}$	5.16 $\times \;10^{3}$	1.46 $\times \;10^{4}$	1.48 $\times \;10^{4}$	1.98 $\times \;10^{3}$	1.07 $\times \;10^{4}$	1.15 $\times \;10^{4}$	4.95 $\times \;10^{2}$	2.15 $\times \;10^{3}$	1.03 $\times \;10^{2}$
	Mean	1.01 $\times \;10^{3}$	1.85 $\times \;10^{3}$	5.16 $\times \;10^{4}$	2.01 $\times \;10^{5}$	8.57 $\times \;10^{4}$	1.86 $\times \;10^{4}$	1.63 $\times \;10^{4}$	2.43 $\times \;10^{4}$	9.53 $\times \;10^{3}$	6.93 $\times \;10^{4}$	1.10 $\times \;10^{5}$	2.58 $\times \;10^{3}$	1.25 $\times \;10^{4}$	1.02 $\times \;10^{3}$
F4	Std	1.86 $\times \;10^{2}$	2.46 $\times \;10^{2}$	5.83 $\times \;10^{1}$	1.54 $\times \;10^{2}$	4.03 $\times \;10^{1}$	9.36 $\times \;10^{1}$	2.18 $\times \;10^{2}$	1.56 $\times \;10^{2}$	6.31 $\times \;10^{1}$	6.33 $\times \;10^{1}$	4.40 $\times \;10^{1}$	6.34 $\times \;10^{1}$	6.78 $\times \;10^{1}$	1.06 $\times \;10^{2}$
	Mean	1.26 $\times \;10^{3}$	1.57 $\times \;10^{3}$	2.04 $\times \;10^{3}$	2.92 $\times \;10^{3}$	2.06 $\times \;10^{3}$	2.02 $\times \;10^{3}$	1.71 $\times \;10^{3}$	1.55 $\times \;10^{3}$	1.67 $\times \;10^{3}$	1.92 $\times \;10^{3}$	2.13 $\times \;10^{3}$	1.64 $\times \;10^{3}$	1.81 $\times \;10^{3}$	1.38 $\times \;10^{3}$
F5	Std	6.18 $\times \;10^{0}$	1.18 $\times \;10^{1}$	4.61 $\times \;10^{0}$	6.83 $\times \;10^{0}$	4.13 $\times \;10^{0}$	9.24 $\times \;10^{0}$	1.32 $\times \;10^{1}$	6.33 $\times \;10^{0}$	4.23 $\times \;10^{0}$	5.47 $\times \;10^{0}$	4.20 $\times \;10^{0}$	5.87 $\times \;10^{0}$	4.68 $\times \;10^{0}$	5.81 $\times \;10^{0}$
	Mean	6.40 $\times \;10^{2}$	6.33 $\times \;10^{2}$	7.05 $\times \;10^{2}$	7.61 $\times \;10^{2}$	7.13 $\times \;10^{2}$	7.03 $\times \;10^{2}$	6.79 $\times \;10^{2}$	6.78 $\times \;10^{2}$	6.90 $\times \;10^{2}$	6.97 $\times \;10^{2}$	7.13 $\times \;10^{2}$	6.41 $\times \;10^{2}$	6.96 $\times \;10^{2}$	6.65 $\times \;10^{2}$
F6	Std	3.34 $\times \;10^{2}$	2.11 $\times \;10^{2}$	3.06 $\times \;10^{2}$	9.98 $\times \;10^{2}$	8.14 $\times \;10^{1}$	2.06 $\times \;10^{2}$	2.64 $\times \;10^{2}$	1.90 $\times \;10^{2}$	1.06 $\times \;10^{2}$	1.25 $\times \;10^{2}$	7.80 $\times \;10^{1}$	1.16 $\times \;10^{2}$	1.26 $\times \;10^{2}$	2.69 $\times \;10^{2}$
	Mean	2.63 $\times \;10^{3}$	2.54 $\times \;10^{3}$	4.09 $\times \;10^{3}$	1.17 $\times \;10^{4}$	3.84 $\times \;10^{3}$	3.68 $\times \;10^{3}$	2.98 $\times \;10^{3}$	3.42 $\times \;10^{3}$	3.76 $\times \;10^{3}$	3.69 $\times \;10^{3}$	4.04 $\times \;10^{3}$	2.35 $\times \;10^{3}$	3.66 $\times \;10^{3}$	2.37 $\times \;10^{3}$
F7	Std	1.86 $\times \;10^{2}$	1.82 $\times \;10^{2}$	7.82 $\times \;10^{1}$	1.79 $\times \;10^{2}$	4.08 $\times \;10^{1}$	1.14 $\times \;10^{2}$	2.21 $\times \;10^{2}$	1.19 $\times \;10^{2}$	5.78 $\times \;10^{1}$	8.18 $\times \;10^{1}$	5.16 $\times \;10^{1}$	5.98 $\times \;10^{1}$	7.18 $\times \;10^{1}$	1.08 $\times \;10^{2}$
	Mean	1.53 $\times \;10^{3}$	1.94 $\times \;10^{3}$	2.41 $\times \;10^{3}$	3.33 $\times \;10^{3}$	2.54 $\times \;10^{3}$	2.40 $\times \;10^{3}$	2.15 $\times \;10^{3}$	2.00 $\times \;10^{3}$	2.12 $\times \;10^{3}$	2.36 $\times \;10^{3}$	2.59 $\times \;10^{3}$	1.98 $\times \;10^{3}$	2.27 $\times \;10^{3}$	1.68 $\times \;10^{3}$
F8	Std	1.79 $\times \;10^{4}$	1.49 $\times \;10^{4}$	1.09 $\times \;10^{4}$	2.55 $\times \;10^{4}$	3.31 $\times \;10^{3}$	1.28 $\times \;10^{4}$	1.19 $\times \;10^{4}$	1.07 $\times \;10^{4}$	4.99 $\times \;10^{3}$	6.22 $\times \;10^{3}$	3.98 $\times \;10^{3}$	6.60 $\times \;10^{3}$	5.54 $\times \;10^{3}$	3.51 $\times \;10^{3}$
	Mean	7.05 $\times \;10^{4}$	4.42 $\times \;10^{4}$	9.48 $\times \;10^{4}$	1.54 $\times \;10^{5}$	8.27 $\times \;10^{4}$	9.02 $\times \;10^{4}$	7.99 $\times \;10^{4}$	3.85 $\times \;10^{4}$	6.93 $\times \;10^{4}$	6.80 $\times \;10^{4}$	7.87 $\times \;10^{4}$	4.87 $\times \;10^{4}$	6.45 $\times \;10^{4}$	3.78 $\times \;10^{4}$
F9	Std	2.64 $\times \;10^{3}$	6.73 $\times \;10^{2}$	6.39 $\times \;10^{2}$	8.89 $\times \;10^{2}$	9.94 $\times \;10^{2}$	1.30 $\times \;10^{3}$	4.65 $\times \;10^{3}$	1.44 $\times \;10^{3}$	1.78 $\times \;10^{3}$	1.35 $\times \;10^{3}$	5.20 $\times \;10^{2}$	6.21 $\times \;10^{2}$	1.68 $\times \;10^{3}$	1.13 $\times \;10^{3}$
	Mean	3.12 $\times \;10^{4}$	3.38 $\times \;10^{4}$	3.30 $\times \;10^{4}$	3.40 $\times \;10^{4}$	3.21 $\times \;10^{4}$	3.15 $\times \;10^{4}$	2.93 $\times \;10^{4}$	1.98 $\times \;10^{4}$	2.45 $\times \;10^{4}$	2.93 $\times \;10^{4}$	3.29 $\times \;10^{4}$	3.07 $\times \;10^{4}$	2.73 $\times \;10^{4}$	1.90 $\times \;10^{4}$
F10	Std	1.68 $\times \;10^{4}$	3.85 $\times \;10^{4}$	2.66 $\times \;10^{4}$	1.58 $\times \;10^{5}$	3.97 $\times \;10^{4}$	4.92 $\times \;10^{5}$	5.37 $\times \;10^{4}$	3.46 $\times \;10^{4}$	3.88 $\times \;10^{4}$	3.60 $\times \;10^{4}$	5.00 $\times \;10^{4}$	1.56 $\times \;10^{4}$	2.16 $\times \;10^{4}$	8.64 $\times \;10^{3}$
	Mean	5.05 $\times \;10^{4}$	1.93 $\times \;10^{5}$	1.75 $\times \;10^{5}$	5.36 $\times \;10^{5}$	2.13 $\times \;10^{5}$	5.29 $\times \;10^{5}$	2.18 $\times \;10^{5}$	7.35 $\times \;10^{4}$	1.52 $\times \;10^{5}$	1.86 $\times \;10^{5}$	2.66 $\times \;10^{5}$	9.05 $\times \;10^{4}$	1.62 $\times \;10^{5}$	2.39 $\times \;10^{4}$
F11	Std	4.98 $\times \;10^{7}$	4.91 $\times \;10^{8}$	1.27 $\times \;10^{10}$	6.92 $\times \;10^{10}$	1.62 $\times \;10^{10}$	1.20 $\times \;10^{10}$	2.75 $\times \;10^{9}$	3.56 $\times \;10^{10}$	3.72 $\times \;10^{9}$	1.74 $\times \;10^{10}$	1.49 $\times \;10^{10}$	3.02 $\times \;10^{8}$	3.94 $\times \;10^{9}$	2.31 $\times \;10^{8}$
	Mean	1.02 $\times \;10^{8}$	5.75 $\times \;10^{8}$	9.45 $\times \;10^{10}$	2.91 $\times \;10^{11}$	1.85 $\times \;10^{11}$	3.00 $\times \;10^{10}$	7.94 $\times \;10^{9}$	6.44 $\times \;10^{10}$	1.15 $\times \;10^{10}$	1.52 $\times \;10^{11}$	2.06 $\times \;10^{11}$	8.36 $\times \;10^{8}$	1.82 $\times \;10^{10}$	4.46 $\times \;10^{8}$
F12	Std	1.13 $\times \;10^{5}$	7.19 $\times \;10^{3}$	2.87 $\times \;10^{9}$	1.39 $\times \;10^{10}$	5.43 $\times \;10^{9}$	2.13 $\times \;10^{9}$	2.48 $\times \;10^{8}$	6.26 $\times \;10^{9}$	1.42 $\times \;10^{8}$	3.91 $\times \;10^{9}$	5.92 $\times \;10^{9}$	1.01 $\times \;10^{5}$	1.15 $\times \;10^{9}$	5.31 $\times \;10^{6}$
	Mean	5.69 $\times \;10^{4}$	1.38 $\times \;10^{4}$	1.77 $\times \;10^{10}$	6.86 $\times \;10^{10}$	4.60 $\times \;10^{10}$	3.37 $\times \;10^{9}$	3.42 $\times \;10^{8}$	6.98 $\times \;10^{9}$	2.73 $\times \;10^{8}$	3.12 $\times \;10^{10}$	4.76 $\times \;10^{10}$	1.85 $\times \;10^{5}$	1.76 $\times \;10^{9}$	1.71 $\times \;10^{6}$
F13	Std	1.41 $\times \;10^{6}$	6.59 $\times \;10^{6}$	2.20 $\times \;10^{7}$	1.66 $\times \;10^{8}$	4.40 $\times \;10^{7}$	2.28 $\times \;10^{7}$	9.15 $\times \;10^{6}$	6.41 $\times \;10^{6}$	3.83 $\times \;10^{6}$	2.21 $\times \;10^{7}$	5.02 $\times \;10^{7}$	1.77 $\times \;10^{6}$	6.73 $\times \;10^{6}$	3.54 $\times \;10^{6}$
	Mean	3.02 $\times \;10^{6}$	7.05 $\times \;10^{6}$	5.71 $\times \;10^{7}$	2.29 $\times \;10^{8}$	8.72 $\times \;10^{7}$	2.98 $\times \;10^{7}$	1.54 $\times \;10^{7}$	3.18 $\times \;10^{6}$	1.16 $\times \;10^{7}$	4.31 $\times \;10^{7}$	1.14 $\times \;10^{8}$	4.39 $\times \;10^{6}$	1.72 $\times \;10^{7}$	5.40 $\times \;10^{6}$
F14	Std	1.95 $\times \;10^{4}$	6.59 $\times \;10^{3}$	1.50 $\times \;10^{9}$	1.09 $\times \;10^{10}$	4.40 $\times \;10^{9}$	1.79 $\times \;10^{9}$	9.64 $\times \;10^{7}$	3.29 $\times \;10^{9}$	1.35 $\times \;10^{7}$	3.60 $\times \;10^{9}$	4.48 $\times \;10^{9}$	3.88 $\times \;10^{3}$	2.20 $\times \;10^{8}$	2.41 $\times \;10^{6}$
	Mean	1.65 $\times \;10^{4}$	7.37 $\times \;10^{3}$	5.62 $\times \;10^{9}$	3.01 $\times \;10^{10}$	2.22 $\times \;10^{10}$	1.65 $\times \;10^{9}$	6.39 $\times \;10^{7}$	1.28 $\times \;10^{9}$	1.86 $\times \;10^{7}$	1.76 $\times \;10^{10}$	2.63 $\times \;10^{10}$	1.01 $\times \;10^{4}$	2.50 $\times \;10^{8}$	9.25 $\times \;10^{5}$
F15	Std	1.83 $\times \;10^{3}$	1.46 $\times \;10^{3}$	1.12 $\times \;10^{3}$	5.51 $\times \;10^{3}$	2.64 $\times \;10^{3}$	1.25 $\times \;10^{3}$	1.55 $\times \;10^{3}$	3.67 $\times \;10^{3}$	1.29 $\times \;10^{3}$	1.97 $\times \;10^{3}$	2.31 $\times \;10^{3}$	5.54 $\times \;10^{2}$	1.64 $\times \;10^{3}$	5.85 $\times \;10^{2}$
	Mean	6.16 $\times \;10^{3}$	1.13 $\times \;10^{4}$	1.51 $\times \;10^{4}$	3.00 $\times \;10^{4}$	2.20 $\times \;10^{4}$	1.23 $\times \;10^{4}$	9.41 $\times \;10^{3}$	1.13 $\times \;10^{4}$	1.07 $\times \;10^{4}$	1.76 $\times \;10^{4}$	2.51 $\times \;10^{4}$	1.04 $\times \;10^{4}$	1.33 $\times \;10^{4}$	6.50 $\times \;10^{3}$
F16	Std	6.63 $\times \;10^{2}$	5.52 $\times \;10^{2}$	4.27 $\times \;10^{4}$	1.25 $\times \;10^{7}$	8.43 $\times \;10^{6}$	6.52 $\times \;10^{4}$	1.29 $\times \;10^{3}$	1.45 $\times \;10^{6}$	2.40 $\times \;10^{3}$	2.20 $\times \;10^{6}$	1.54 $\times \;10^{7}$	4.29 $\times \;10^{2}$	1.15 $\times \;10^{4}$	7.25 $\times \;10^{2}$
	Mean	4.77 $\times \;10^{3}$	8.46 $\times \;10^{3}$	5.76 $\times \;10^{4}$	7.37 $\times \;10^{6}$	8.79 $\times \;10^{6}$	4.32 $\times \;10^{4}$	9.63 $\times \;10^{3}$	3.90 $\times \;10^{5}$	8.85 $\times \;10^{3}$	2.26 $\times \;10^{6}$	1.63 $\times \;10^{7}$	7.02 $\times \;10^{3}$	1.50 $\times \;10^{4}$	5.65 $\times \;10^{3}$
F17	Std	3.79 $\times \;10^{6}$	1.27 $\times \;10^{7}$	6.34 $\times \;10^{7}$	3.49 $\times \;10^{8}$	9.00 $\times \;10^{7}$	3.11 $\times \;10^{7}$	1.33 $\times \;10^{7}$	1.61 $\times \;10^{7}$	4.27 $\times \;10^{6}$	3.14 $\times \;10^{7}$	1.54 $\times \;10^{8}$	2.45 $\times \;10^{6}$	9.85 $\times \;10^{6}$	4.15 $\times \;10^{6}$
	Mean	6.74 $\times \;10^{6}$	1.74 $\times \;10^{7}$	1.34 $\times \;10^{8}$	4.72 $\times \;10^{8}$	1.74 $\times \;10^{8}$	5.06 $\times \;10^{7}$	2.56 $\times \;10^{7}$	6.58 $\times \;10^{6}$	8.87 $\times \;10^{6}$	7.15 $\times \;10^{7}$	3.24 $\times \;10^{8}$	5.45 $\times \;10^{6}$	2.22 $\times \;10^{7}$	1.00 $\times \;10^{7}$
F18	Std	7.09 $\times \;10^{3}$	6.12 $\times \;10^{3}$	1.49 $\times \;10^{9}$	9.60 $\times \;10^{9}$	4.56 $\times \;10^{9}$	4.99 $\times \;10^{8}$	1.65 $\times \;10^{8}$	4.50 $\times \;10^{9}$	5.13 $\times \;10^{7}$	3.29 $\times \;10^{9}$	5.20 $\times \;10^{9}$	9.26 $\times \;10^{3}$	2.23 $\times \;10^{8}$	4.39 $\times \;10^{5}$
	Mean	1.12 $\times \;10^{4}$	7.31 $\times \;10^{3}$	5.33 $\times \;10^{9}$	3.02 $\times \;10^{10}$	2.50 $\times \;10^{10}$	1.18 $\times \;10^{9}$	1.37 $\times \;10^{8}$	2.27 $\times \;10^{9}$	4.91 $\times \;10^{7}$	1.53 $\times \;10^{10}$	2.64 $\times \;10^{10}$	1.19 $\times \;10^{4}$	2.77 $\times \;10^{8}$	6.90 $\times \;10^{5}$
F19	Std	1.37 $\times \;10^{3}$	3.20 $\times \;10^{2}$	3.30 $\times \;10^{2}$	3.29 $\times \;10^{2}$	2.07 $\times \;10^{2}$	3.56 $\times \;10^{2}$	7.95 $\times \;10^{2}$	9.30 $\times \;10^{2}$	4.99 $\times \;10^{2}$	5.12 $\times \;10^{2}$	2.40 $\times \;10^{2}$	2.80 $\times \;10^{2}$	4.98 $\times \;10^{2}$	6.70 $\times \;10^{2}$
	Mean	6.33 $\times \;10^{3}$	8.31 $\times \;10^{3}$	8.11 $\times \;10^{3}$	8.83 $\times \;10^{3}$	7.90 $\times \;10^{3}$	7.91 $\times \;10^{3}$	7.21 $\times \;10^{3}$	6.06 $\times \;10^{3}$	6.25 $\times \;10^{3}$	6.95 $\times \;10^{3}$	8.00 $\times \;10^{3}$	7.36 $\times \;10^{3}$	6.70 $\times \;10^{3}$	5.80 $\times \;10^{3}$
F20	Std	2.15 $\times \;10^{2}$	2.36 $\times \;10^{2}$	1.01 $\times \;10^{2}$	2.43 $\times \;10^{2}$	2.28 $\times \;10^{2}$	2.31 $\times \;10^{2}$	2.12 $\times \;10^{2}$	2.96 $\times \;10^{2}$	2.44 $\times \;10^{2}$	1.82 $\times \;10^{2}$	2.44 $\times \;10^{2}$	4.70 $\times \;10^{1}$	1.27 $\times \;10^{2}$	1.46 $\times \;10^{2}$
	Mean	3.10 $\times \;10^{3}$	3.42 $\times \;10^{3}$	4.18 $\times \;10^{3}$	5.34 $\times \;10^{3}$	4.61 $\times \;10^{3}$	4.29 $\times \;10^{3}$	4.07 $\times \;10^{3}$	4.15 $\times \;10^{3}$	4.44 $\times \;10^{3}$	4.51 $\times \;10^{3}$	5.05 $\times \;10^{3}$	3.40 $\times \;10^{3}$	4.20 $\times \;10^{3}$	3.21 $\times \;10^{3}$
F21	Std	5.64 $\times \;10^{3}$	8.54 $\times \;10^{2}$	7.43 $\times \;10^{2}$	1.47 $\times \;10^{3}$	8.58 $\times \;10^{2}$	1.81 $\times \;10^{3}$	4.62 $\times \;10^{3}$	2.73 $\times \;10^{3}$	1.93 $\times \;10^{3}$	8.77 $\times \;10^{2}$	7.06 $\times \;10^{2}$	7.02 $\times \;10^{2}$	1.49 $\times \;10^{3}$	1.21 $\times \;10^{3}$
	Mean	2.99 $\times \;10^{4}$	3.61 $\times \;10^{4}$	3.52 $\times \;10^{4}$	3.65 $\times \;10^{4}$	3.48 $\times \;10^{4}$	3.27 $\times \;10^{4}$	3.04 $\times \;10^{4}$	2.39 $\times \;10^{4}$	2.73 $\times \;10^{4}$	3.22 $\times \;10^{4}$	3.54 $\times \;10^{4}$	3.31 $\times \;10^{4}$	3.04 $\times \;10^{4}$	2.08 $\times \;10^{4}$
F22	Std	1.25 $\times \;10^{2}$	2.00 $\times \;10^{2}$	1.43 $\times \;10^{2}$	6.14 $\times \;10^{2}$	1.80 $\times \;10^{2}$	4.56 $\times \;10^{2}$	2.41 $\times \;10^{2}$	3.10 $\times \;10^{2}$	5.48 $\times \;10^{2}$	4.85 $\times \;10^{2}$	3.16 $\times \;10^{2}$	5.22 $\times \;10^{1}$	2.18 $\times \;10^{2}$	1.25 $\times \;10^{2}$
	Mean	3.58 $\times \;10^{3}$	3.59 $\times \;10^{3}$	5.26 $\times \;10^{3}$	8.03 $\times \;10^{3}$	5.72 $\times \;10^{3}$	6.32 $\times \;10^{3}$	4.82 $\times \;10^{3}$	5.24 $\times \;10^{3}$	5.89 $\times \;10^{3}$	7.33 $\times \;10^{3}$	6.74 $\times \;10^{3}$	4.00 $\times \;10^{3}$	5.09 $\times \;10^{3}$	3.59 $\times \;10^{3}$
F23	Std	2.35 $\times \;10^{2}$	2.62 $\times \;10^{2}$	2.63 $\times \;10^{2}$	1.54 $\times \;10^{3}$	2.65 $\times \;10^{3}$	9.98 $\times \;10^{2}$	5.28 $\times \;10^{2}$	8.76 $\times \;10^{2}$	7.31 $\times \;10^{2}$	7.44 $\times \;10^{2}$	8.58 $\times \;10^{2}$	6.81 $\times \;10^{1}$	3.85 $\times \;10^{2}$	1.73 $\times \;10^{2}$
	Mean	4.36 $\times \;10^{3}$	4.34 $\times \;10^{3}$	7.43 $\times \;10^{3}$	1.31 $\times \;10^{4}$	9.65 $\times \;10^{3}$	1.02 $\times \;10^{4}$	6.19 $\times \;10^{3}$	7.00 $\times \;10^{3}$	8.47 $\times \;10^{3}$	1.20 $\times \;10^{4}$	1.04 $\times \;10^{4}$	4.56 $\times \;10^{3}$	6.60 $\times \;10^{3}$	4.26 $\times \;10^{3}$
F24	Std	1.44 $\times \;10^{2}$	2.89 $\times \;10^{2}$	2.94 $\times \;10^{3}$	1.46 $\times \;10^{4}$	2.35 $\times \;10^{3}$	2.27 $\times \;10^{3}$	5.56 $\times \;10^{3}$	4.47 $\times \;10^{3}$	5.11 $\times \;10^{2}$	2.08 $\times \;10^{3}$	1.22 $\times \;10^{3}$	3.87 $\times \;10^{2}$	9.58 $\times \;10^{2}$	7.28 $\times \;10^{1}$
	Mean	3.70 $\times \;10^{3}$	4.46 $\times \;10^{3}$	2.26 $\times \;10^{4}$	9.62 $\times \;10^{4}$	2.59 $\times \;10^{4}$	1.44 $\times \;10^{4}$	9.79 $\times \;10^{3}$	1.34 $\times \;10^{4}$	6.81 $\times \;10^{3}$	2.41 $\times \;10^{4}$	3.03 $\times \;10^{4}$	5.01 $\times \;10^{3}$	9.01 $\times \;10^{3}$	3.70 $\times \;10^{3}$
F25	Std	2.19 $\times \;10^{3}$	2.47 $\times \;10^{3}$	3.38 $\times \;10^{3}$	9.66 $\times \;10^{3}$	2.20 $\times \;10^{3}$	1.52 $\times \;10^{4}$	3.97 $\times \;10^{3}$	7.23 $\times \;10^{3}$	2.65 $\times \;10^{3}$	3.14 $\times \;10^{3}$	1.85 $\times \;10^{3}$	1.49 $\times \;10^{3}$	3.52 $\times \;10^{3}$	2.64 $\times \;10^{3}$
	Mean	1.59 $\times \;10^{4}$	1.63 $\times \;10^{4}$	4.22 $\times \;10^{4}$	7.74 $\times \;10^{4}$	5.02 $\times \;10^{4}$	3.85 $\times \;10^{4}$	2.69 $\times \;10^{4}$	3.67 $\times \;10^{4}$	3.23 $\times \;10^{4}$	4.80 $\times \;10^{4}$	5.34 $\times \;10^{4}$	2.11 $\times \;10^{4}$	3.35 $\times \;10^{4}$	1.60 $\times \;10^{4}$
F26	Std	8.15 $\times \;10^{1}$	1.41 $\times \;10^{2}$	5.68 $\times \;10^{2}$	1.79 $\times \;10^{3}$	2.31 $\times \;10^{3}$	1.92 $\times \;10^{3}$	4.64 $\times \;10^{2}$	1.48 $\times \;10^{3}$	1.13 $\times \;10^{3}$	1.13 $\times \;10^{3}$	1.57 $\times \;10^{3}$	9.73 $\times \;10^{1}$	4.87 $\times \;10^{2}$	1.05 $\times \;10^{2}$
	Mean	3.78 $\times \;10^{3}$	3.79 $\times \;10^{3}$	8.70 $\times \;10^{3}$	1.53 $\times \;10^{4}$	1.16 $\times \;10^{4}$	7.87 $\times \;10^{3}$	4.62 $\times \;10^{3}$	5.95 $\times \;10^{3}$	7.02 $\times \;10^{3}$	1.32 $\times \;10^{4}$	1.50 $\times \;10^{4}$	4.23 $\times \;10^{3}$	5.31 $\times \;10^{3}$	3.85 $\times \;10^{3}$
F27	Std	3.48 $\times \;10^{2}$	1.27 $\times \;10^{3}$	3.65 $\times \;10^{3}$	8.84 $\times \;10^{3}$	2.32 $\times \;10^{3}$	2.40 $\times \;10^{3}$	7.08 $\times \;10^{3}$	6.83 $\times \;10^{3}$	8.50 $\times \;10^{2}$	2.19 $\times \;10^{3}$	1.62 $\times \;10^{3}$	4.81 $\times \;10^{2}$	1.63 $\times \;10^{3}$	7.13 $\times \;10^{1}$
	Mean	4.07 $\times \;10^{3}$	6.29 $\times \;10^{3}$	2.76 $\times \;10^{4}$	6.31 $\times \;10^{4}$	2.89 $\times \;10^{4}$	1.82 $\times \;10^{4}$	1.70 $\times \;10^{4}$	2.00 $\times \;10^{4}$	9.49 $\times \;10^{3}$	3.13 $\times \;10^{4}$	3.02 $\times \;10^{4}$	5.98 $\times \;10^{3}$	1.19 $\times \;10^{4}$	3.73 $\times \;10^{3}$
F28	Std	6.90 $\times \;10^{2}$	1.91 $\times \;10^{3}$	1.10 $\times \;10^{4}$	1.46 $\times \;10^{6}$	4.84 $\times \;10^{5}$	2.16 $\times \;10^{3}$	2.30 $\times \;10^{3}$	9.79 $\times \;10^{4}$	1.83 $\times \;10^{3}$	1.48 $\times \;10^{5}$	5.99 $\times \;10^{5}$	4.70 $\times \;10^{2}$	2.95 $\times \;10^{3}$	5.35 $\times \;10^{2}$
	Mean	6.98 $\times \;10^{3}$	9.31 $\times \;10^{3}$	3.20 $\times \;10^{4}$	1.26 $\times \;10^{6}$	6.66 $\times \;10^{5}$	1.61 $\times \;10^{4}$	1.23 $\times \;10^{4}$	5.46 $\times \;10^{4}$	1.30 $\times \;10^{4}$	1.67 $\times \;10^{5}$	8.15 $\times \;10^{5}$	1.03 $\times \;10^{4}$	1.80 $\times \;10^{4}$	8.28 $\times \;10^{3}$
F29	Std	5.40 $\times \;10^{5}$	5.33 $\times \;10^{5}$	2.37 $\times \;10^{9}$	1.32 $\times \;10^{10}$	4.79 $\times \;10^{9}$	3.63 $\times \;10^{9}$	1.23 $\times \;10^{8}$	6.08 $\times \;10^{9}$	3.69 $\times \;10^{8}$	6.38 $\times \;10^{9}$	6.77 $\times \;10^{9}$	3.42 $\times \;10^{6}$	1.12 $\times \;10^{9}$	8.65 $\times \;10^{6}$
	Mean	8.27 $\times \;10^{5}$	7.05 $\times \;10^{5}$	1.21 $\times \;10^{10}$	5.49 $\times \;10^{10}$	4.26 $\times \;10^{10}$	4.03 $\times \;10^{9}$	2.68 $\times \;10^{8}$	5.75 $\times \;10^{9}$	8.27 $\times \;10^{8}$	2.76 $\times \;10^{10}$	4.21 $\times \;10^{10}$	7.67 $\times \;10^{6}$	2.13 $\times \;10^{9}$	1.64 $\times \;10^{7}$
